# *In Crystallo* Hydrogenation of Nitrous Oxide Using a Rhodium(II) Hydride Complex

**DOI:** 10.1021/acs.inorgchem.6c04272

**Published:** 2026-09-11

**Authors:** Thomas M. Hood, Sophie H. Dewick, Jie Liu, Emma Richards, Adrian B. Chaplin

**Affiliations:** † Department of Chemistry, 2707 University of Warwick, Gibbet Hill Road, Coventry, CV4 7AL, U.K.; ‡ Department of Physics, University of Warwick, Gibbet Hill Road, Coventry, CV4 7AL, U.K.; § School of Chemistry, Cardiff University, Translational Research Hub, Cardiff, CF24 4HQ, U.K.

## Abstract

The stoichiometric and catalytic hydrogenation of nitrous oxide mediated by gas/solid reactions of rhodium­(II) pincer complexes [Rh­(PNP-*t*Bu)­X]^+^ (X = CH_3_, H, OH; PNP-*t*Bu = 2,6-(*t*Bu_2_PCH_2_)_2_C_5_H_3_N) is reported. A reaction sequence involving Rh­(II)–CH_3_ → Rh­(II)–H → Rh­(II)–OH → Rh­(II)–H bond interconversions has been structurally mapped out by single-crystal X-ray diffraction and verified by EPR spectroscopy. These rhodium­(II) metalloradicals are sufficiently robust to catalyze the hydrogenation of nitrous oxide at room temperature with modest productivity (8.3 apparent turnovers). The relative rates of the stoichiometric transformations suggest that Rh–O bond hydrogenolysis is turnover-limiting.

## Graphical abstract

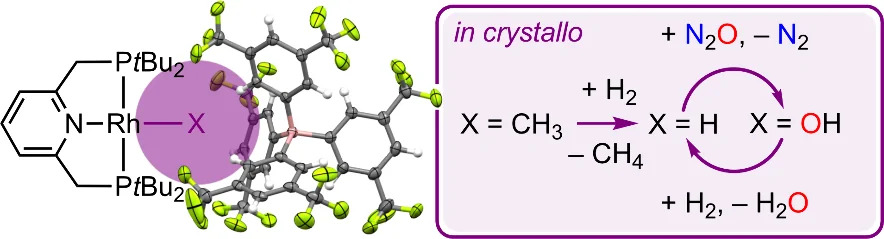

Nitrous oxide (N_2_O) is a potent greenhouse gas and ozone depleting substance.^1^ Within the nitrogen cycle, N_2_O is consumed by denitrifying microorganisms through 2e^–^/2H^+^ reduction to N_2_ and H_2_O, catalyzed by the copper-containing enzyme nitrous oxide reductase.^2^ Hydrogenation is an alternative way of achieving this outcome in a lab setting but typically requires elevated temperatures and precious metal-based heterogeneous catalysts.^3^ While O atom insertion into M–H bonds is an established N_2_O activation manifold,^4^ few transition metal complexes are known to catalyze the homogeneous hydrogenation of N_2_O.^5–8^ Hydrogenolysis by addition of H_2_ across M–O bonds is not well-documented, and onward reactivity of metal hydroxides, such as those resulting from activation of N_2_O, is generally induced by protonolysis (Figure 1A).^5,6,9^

**Figure 1:**
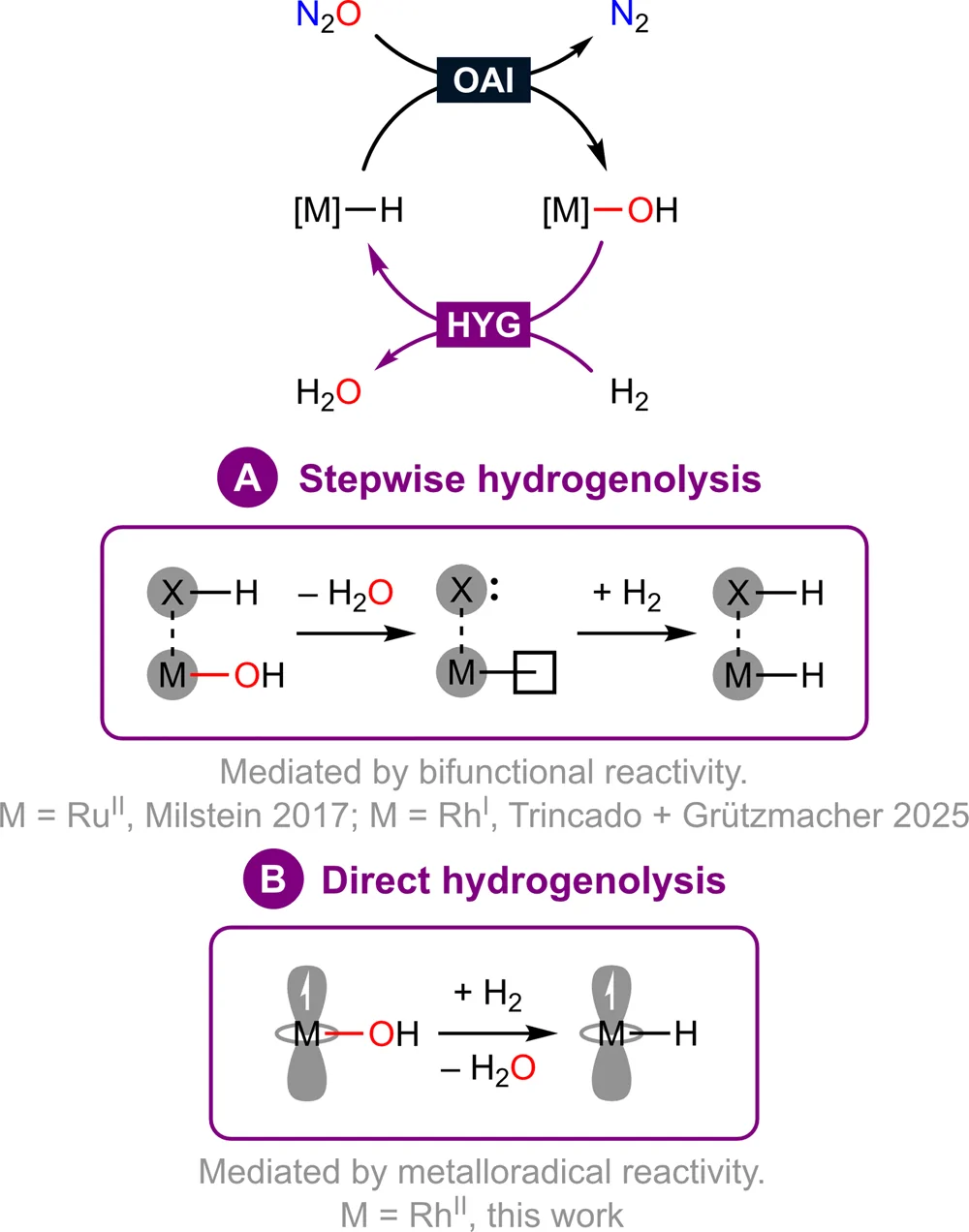
Transition metal catalyzed hydrogenation of N_2_O proceeding by O atom insertion (OAI) into a M–H bond followed by (A) stepwise or (B) direct hydrogenolysis (HYG) of the resulting M–OH bond.

Inspired by the work of Campos et al. on the organometallic chemistry of iridium­(II) pincer complexes^10,11^ and building upon developments in our laboratories,^7,12,13^ we set about examining the hydrogenation of N_2_O using the novel rhodium­(II) hydride complex [Rh­(PNP-*t*Bu)­H]­[BAr^F^_4_] **1** (PNP-*t*Bu = 2,6-(*t*Bu_2_PCH_2_)_2_C_5_H_3_N, Ar^F^ = 3,5-(CF_3_)_2_C_6_H_3_), hypothesizing that reaction of H_2_ with the open-shell hydroxide derivative [Rh­(PNP-*t*Bu)­(OH)]­[BAr^F^_4_] **2** would be promoted by metalloradical reactivity (Figure 1B). The closest literature precedent for **1** is a square-pyramidal rhodium­(II) hydride prepared in solution by one-electron oxidation of [Rh­(PPh_3_)_3_(CO)­H].^14^ Attempts to access **1** in a similar manner, however, invariably afforded diamagnetic [Rh­(PNP-*t*Bu)­(H_2_)]­[BAr^F^_4_] **3** as the common organometallic product. Recognizing the propensity of the open-shell hydride for intermolecular decomposition in solution, we turned to solid-state molecular organometallic methods to study **1** and assess our hypothesis through gas/solid reactions.^15,16^ We herein report on the *in crystallo* hydrogenation of N_2_O and characterization of the metalloradical intermediates involved in this transformation by single-crystal X-ray diffraction and EPR spectroscopy.

To synthesize **1** in the solid state, a strategy involving treatment of crystalline samples of novel rhodium­(II) methyl complex [Rh­(PNP-*t*Bu)­(CH_3_)]­[BAr^F^_4_] **4** with H_2_ was devised. The required rhodium­(II) precursor was subsequently obtained as dark purple microcrystalline powder following one-electron oxidation of the known rhodium­(I) congener (*E*_1/2_ = −0.64 V vs Fc^+^/Fc, Fc = ferrocene) with Fc­[BAr^F^_4_] in 1,2-difluorobenzene (DFB, Figure 2A).^12,17^ Complex **4** is stable in 2-methyltetrahydrofuran (MeTHF) solution at room temperature, and only ca. 10% decomposition was observed in DFB over 24 h, enabling single crystals of **4** suitable for analysis by X-ray diffraction to be reproducibly obtained by recrystallization from DFB/hexane at room temperature (monoclinic, *P*2_1_/*c*). The resulting solid-state structure is notable for adoption of a square planar metal geometry and a Rh1–C4 distance of 2.094(3) Å. Each [Rh­(PNP-*t*Bu)­(CH_3_)]^+^ cation is surrounded by an octahedral arrangement of [BAr^F^_4_]^−^ anions, and the methyl ligand is notably projected into a cleft of the proximal counterion, which demarcates a cavity about this coordination site that is well-suited to gas/solid reactivity (Figure 2B,C).^15^ Subsequent analysis of the bulk microcrystalline sample by powder X-ray diffraction confirmed exclusive adoption of this morphology. In DFB/MeTHF solution, the presence of a single paramagnetic species of *C*_2v_ symmetry is evident by ^1^H NMR spectroscopy. All the proton environments can be located between δ –70 and +40, and an effective magnetic moment of 1.64 μ_B_ was determined in MeTHF using Evans’ method,^18^ consistent with the expected d^7^-metal configuration. No ^31^P resonance could be located between δ –600 and +600. Assignment as a metal-centered radical was subsequently corroborated by analysis of a frozen MeTHF glass solution of **4** by EPR spectroscopy at 115 K, with **4** observed alongside the corresponding square pyramidal solvent adduct **4·**MeTHF in a 1:1 ratio (Figure 3A). Complex **4** displays rhombic symmetry and a high degree of *g*-anisotropy (*g*_1_ = 3.801, *g*_2_ = 1.851, *g*_3_ = 1.521; *A*_1_ = 298 MHz, *A*_2_ = 191 MHz, *A*_3_ = 181 MHz from coupling to ^103^Rh), characteristic of authentically square planar rhodium­(II) complexes,^19^ while **4·**MeTHF exhibits diagnostically reduced *g*-anisotropy (*g*_1_ = 2.496, *g*_2_ = 2.336, *g*_3_ = 1.971; *A*_1_ = 121 MHz, *A*_2_ = 131 MHz, *A*_3_ = 128 MHz from coupling to ^103^Rh).^12^

**Figure 2:**
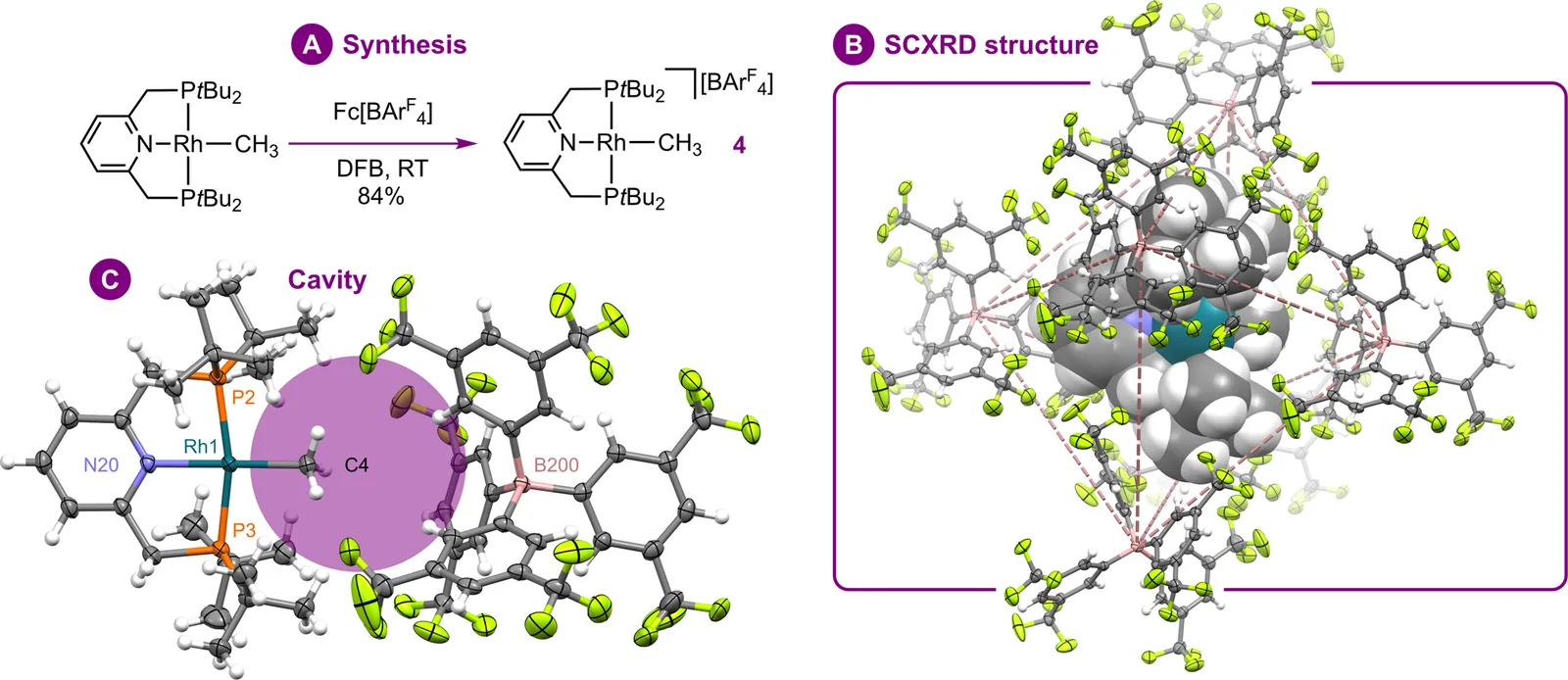
(A) Synthesis and (B, C) structural characterization of rhodium­(II) methyl complex **4**. Insert (B) illustrates packing of the cation (rendered in space fill) within an octahedral arrangement of anions. Insert (C) illustrates positioning of the methyl ligand within the cleft of the proximal [BAr^F^_4_]^−^ counterion. Thermal ellipsoids at 50% probability and minor disordered components omitted for clarity (3 × CF_3_ groups). Selected metrics: Rh1–P2, 2.3160(5) Å; Rh1–P3, 2.3148(5) Å; Rh1–N20, 2.151(2) Å; Rh1–C4, 2.085(2) Å; Rh1–B200, 8.072(2) Å; P2–Rh1–P3, 165.61(2)°; N20–Rh1–C4, 177.73(9)°.

**Figure 3:**
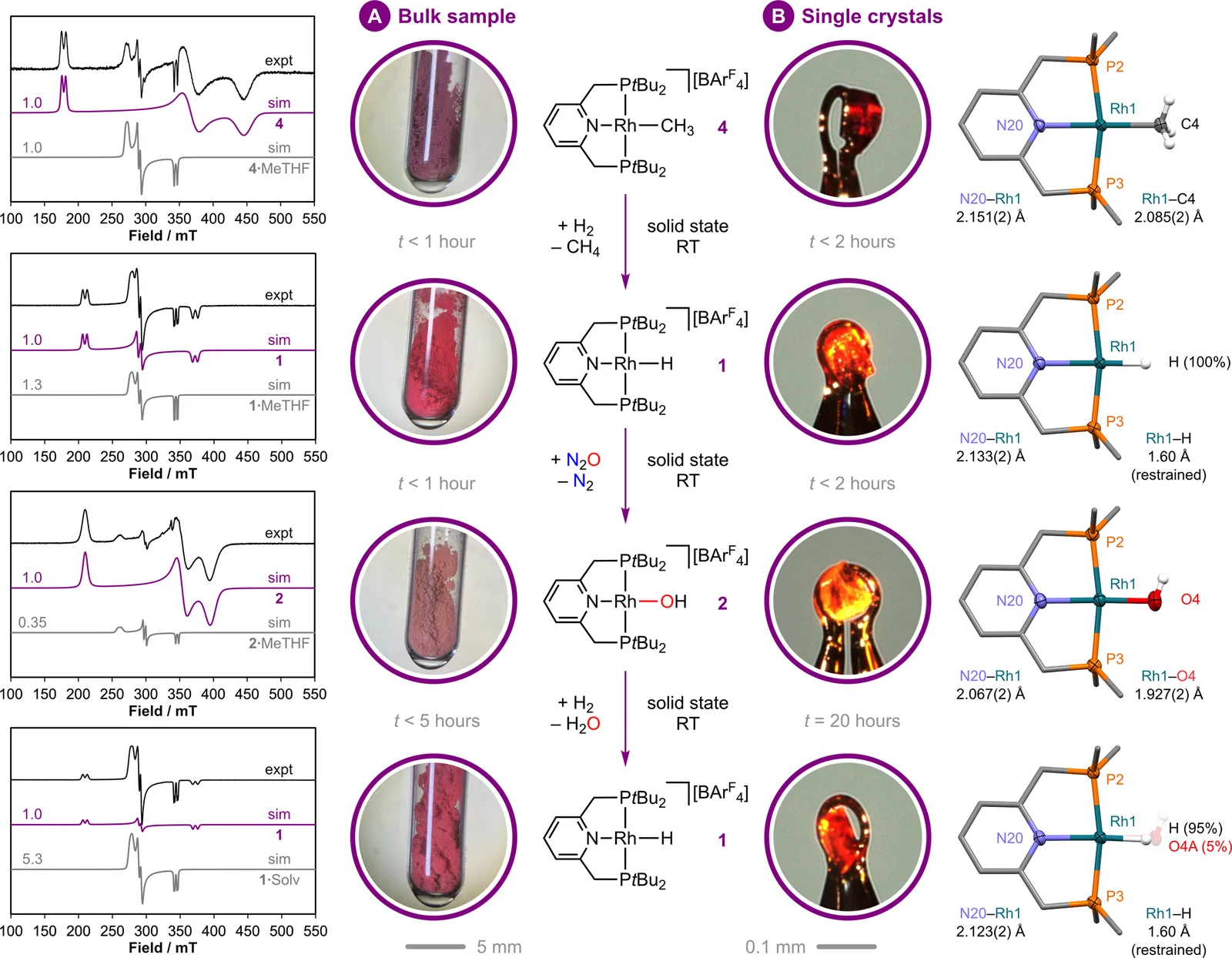
Gas/solid transformations of rhodium­(II) complexes **1**, **2**, and **4** using (A) bulk microcrystalline and (B) single crystal samples, with supporting EPR and crystallographic data. CW X-band EPR spectra were recorded at 115 K using frozen MeTHF glass solutions prepared by dissolution of the sample in thawing MeTHF. Simulated spectra of square planar rhodium­(II) complexes in purple and corresponding solvent adducts in gray (Solv = H_2_O/MeTHF mixture). Truncated solid-state structures shown with selected thermal ellipsoids at 50% probability; counterion and most H atoms omitted for clarity. Additional metrics: **1**, Rh1–P2, 2.2898(6) Å; Rh1–P3, 2.2910(6) Å; P2–Rh1–P3, 168.07(2)°; N20–Rh1–H, 178.98(6)°; **2**, Rh1–P2, 2.3113(7) Å; Rh1–P3, 2.3069(7) Å; P2–Rh1–P3, 168.43(2)°; N20–Rh1–O4, 177.85(9)°; **2** + H_2_ (20 h), Rh1–P2, 2.2920(6) Å; Rh1–P3, 2.2909(6) Å; P2–Rh1–P3, 168.16(2)°; N20–Rh1–H/O4A, 177.2(13)/179.0(11)°.

Treatment of bulk microcrystalline **4** with H_2_ (1 atm) at room temperature resulted in a rapid color change from dark purple to pastel pink, with concomitant liberation of methane verified by analysis of the head space by GC-TCD within 1 h (Figure 3). Conversion into the target rhodium­(II) hydride **1** is supported by appearance of a metal-hydride band in the ATR IR spectrum at 1941 cm^–1^, and repeating the reaction using an ensemble of single crystals of **4** enabled structural elucidation of the product by X-ray diffraction following a single-crystal to single-crystal transformation (1 atm H_2_, RT for 2 h). Statistically significant contractions of the Rh1–P2 (2.2898(6) vs 2.3160(5) Å), Rh1–P3 (2.2910(6) vs 2.3148(5) Å), and Rh1–N20 bonds (2.134(2) vs 2.151(2) Å) are measured relative to **4**, and the hydride ligand in **1** was located from the Fourier difference map and refined with residual electron density at this coordination site <0.3 eÅ^3^ (Figure S35). Decomposition into a diamagnetic mixture of **3** (δ 82.4, ^1^ *J*_RhH_ = 120 Hz) and [Rh­(PNP-*t*Bu)­(MeTHF)]­[BAr^F^_4_] **5** (δ 65.1, ^1^ *J*_RhH_ = 146 Hz; verified by independent synthesis) was observed upon dissolution of **1** in MeTHF at room temperature, with an initial rate and isotope labeling study consistent with bimetallic reductive elimination of H_2_ ^10^ and concurrent, but slower, H atom extraction from the solvent (>95% decomposition within 90 min, 10 mM initial concentration). Formation of **1** was nevertheless verified in frozen MeTHF glass solution by EPR spectroscopy at 115 K through preparation of the sample in thawing MeTHF, as gauged by significant perturbations to the **g**-tensor and the extent of hyperfine coupling (*g*_1_ = 3.246, *g*_2_ = 2.351, *g*_3_ = 1.825; *A*_1_ = 291 MHz, *A*_2_ = 195 MHz, *A*_3_ = 187 MHz from coupling to ^103^Rh; Figure 3A). Installation of the hydride ligands is notably supported by spectroscopic resolution of ^1^H coupling of 40 MHz along the *g*_3_ component of the respective solvent adduct **1·**MeTHF, which is formed with a **1**:**1·**MeTHF ratio of 1.0:1.3 in this case (*g*_1_ = 2.446, *g*_2_ = 2.336, *g*_3_ = 1.975; *A*_1_ = 133 MHz, *A*_2_ = 139 MHz, *A*_3_ = 126 MHz from coupling to ^103^Rh).

The reaction between **1** and N_2_O (1 atm) was thereafter assessed using the samples prepared from **4** as described above. Rapid formation of a light peach microcrystalline powder was observed alongside disappearance of the metal-hydride band in the ATR IR spectrum and generation of N_2_ in the headspace within 1 h (Figure 3). Selective formation of a new square planar rhodium­(II) species is evident from EPR analysis of the bulk microcrystalline sample in frozen MeTHF glass solution at 115 K, and assignment as rhodium­(II) hydroxide **2** was verified *in crystallo* by single-crystal X-ray diffraction (1 atm N_2_O, RT for 2 h). The solid-state structure is notable for a Rh1–O4 bond length of 1.927(3) Å and a reduction in the *trans* Rh1–N20 bond length (2.067(2) vs 2.133(2) Å). Complex **2** is significantly less stable than **1** in solution, decomposing in <5 min upon dissolution in MeTHF at room temperature, but only EPR signatures for **2** (*g*_1_ = 3.231, *g*_2_ = 1.921, *g*_3_ = 1.716; coupling to ^103^Rh not resolved) and **2·**MeTHF (*g*_1_ = 2.601, *g*_2_ = 2.276, *g*_3_ = 1.961; *A*_1_ = 182 MHz, *A*_2_ = 126 MHz, *A*_3_ = 119 MHz from coupling to ^103^Rh) in a 1:0.35 ratio were observed when the sample was prepared in thawing MeTHF.

The stepwise hydrogenation of N_2_O was subsequently realized by treatment of **2** with H_2_ (1 atm) in the solid state at room temperature, although reformation of **1** and loss of H_2_O was slower than the preceding two gas/solid reactions. For the bulk microcrystalline sample, analysis by EPR spectroscopy (115 K, frozen MeTHF glasses) indicated that between 1 to 5 h is required for complete conversion of **2** into **1**. Generation of 1 equiv of water was corroborated by ^1^H NMR spectroscopy following quenching the metalloradical with CO and dissolving the reaction mixture in THF.^20^ Rhodium­(I) carbonyl complex [Rh­(PNP-*t*Bu)­(CO)]­[BAr^F^_4_] **6** was produced and, consistent with Rh–O bond hydrogenolysis by direct reaction with H_2_, when the reaction of **2** was repeated with D_2_, no H/D scrambling was observed in the methylene bridges of pincer ligand backbone (nor other sites).^5^ The rate of hydrogenolysis is appreciably slower when performed using single crystals of **2** (ca. 95% conversion after 20 h) and attributed to a reduction in surface area. Such effects are well-documented in solid-state molecular organometallic chemistry.^15^ Retention of crystallinity and ejection of water from the lattice following three single-crystal to single-crystal transformations is nevertheless quite remarkable!^21^

Having verified the stoichiometric hydrogenation of N_2_O *in crystallo*, the catalytic hydrogenation of N_2_O harnessing this gas/solid reactivity was assessed on a small scale using microcrystalline **4** as the precatalyst and a 1:1 mixture of N_2_O and H_2_ (2 atm) at room temperature for 48 h.^21^ Liberation of CH_4_ and reduction of N_2_O to N_2_ were observed in the headspace, and an apparent turnover number of 8.3 was quantified by GC-TCD using methane as the internal standard (averaged over duplicate runs). Following quenching with CO, the reaction mixture was analyzed by ^31^P NMR spectroscopy for phosphine oxide to signpost the formation of rhodium nanoparticles.^7^ However, consistent with the N_2_O hydrogenation being catalyzed by the rhodium­(II)-based molecular material, **6** was the only phosphorus-containing species observed.

In summary, solid-state organometallic methods have been used to access a highly reactive rhodium­(II) hydride complex and assess its application in the hydrogenation of N_2_O. Through successive gas/solid transformations a reaction sequence involving Rh­(II)–CH_3_ → Rh­(II)–H → Rh­(II)–OH → Rh­(II)–H bond interconversions has been structurally mapped out by single-crystal X-ray diffraction, with the identity of the metalloradicals involved verified by EPR spectroscopy. Hydrogenolysis of the rhodium­(II) hydroxide proceeds more slowly than reaction of the rhodium­(II) hydride with nitrous oxide, and an isotope labeling study is consistent with direct reaction with H_2_ rather than an alternative bifunctional mechanism. The rhodium metalloradicals are sufficiently robust to promote the catalytic hydrogenation of N_2_O at room temperature, with an apparent turnover >8 and turnover limiting Rh–O bond hydrogenolysis inferred from relative rate of the stoichiometric transformations. While the reduction of N_2_O using H_2_ is not a practical remediation method,^6,22^ this transformation is of fundamental importance for learning how to design efficient catalysts for the abatement and repurposing of N_2_O. This work highlights the potential of site-isolated late transition-metal metalloradicals to engage in productive catalytic reactivity.

## Safety Statement

**Caution!** Mixtures of N_2_O and H_2_ are potentially explosive and should be handled with extreme care. This risk is compounded by the potential to form explosive mixtures of O_2_ and H_2_ in the presence of a metal catalyst. Hydrogenation reactions were performed on the smallest practical scale under relatively mild conditions behind a blast shield as described in the Supporting Information.

## Supplementary Material



## Notes

Full experimental details and selected spectroscopic and crystallographic data^23^ (PDF)

## References

[1] a Calvin, K. ; Dasgupta, D. ; Krinner, G. ; Mukherji, A. ; Thorne, P. W. ; Trisos, C. ; Romero, J. ; Aldunce, P. ; Barrett, K. ; Blanco, G. ; Cheung, W. W. L. ; Connors, S. ; Denton, F. ; Diongue-Niang, A. ; Dodman, D. ; Garschagen, M. ; Geden, O. ; Hayward, B. ; Jones, C. ; Jotzo, F. ; Krug, T. ; Lasco, R. ; Lee, Y.-Y. ; Masson-Delmotte, V. ; Meinshausen, M. ; Mintenbeck, K. ; Mokssit, A. ; Otto, F. E. L. ; Pathak, M. ; Pirani, A. ; Poloczanska, E. ; Pörtner, H.-O. ; Revi, A. ; Roberts, D. C. ; Roy, J. ; Ruane, A. C. ; Skea, J. ; Shukla, P. R. ; Slade, R. ; Slangen, A. ; Sokona, Y. ; Sörensson, A. A. ; Tignor, M. ; Vuuren, D. van. ; Wei, Y.-M. ; Winkler, H. ; Zhai, P. ; Zommers, Z. ; Hourcade, J.-C. ; Johnson, F. X. ; Pachauri, S. ; Simpson, N. P. ; Singh, C. ; Thomas, A. ; Totin, E. ; Alegría, A. ; Armour, K. ; Bednar-Friedl, B. ; Blok, K. ; Cissé, G. ; Dentener, F. ; Eriksen, S. ; Fischer, E. ; Garner, G. ; Guivarch, C. ; Haasnoot, M. ; Hansen, G. ; Hauser, M. ; Hawkins, E. ; Hermans, T. ; Kopp, R. ; Leprince-Ringuet, N. ; Lewis, J. ; Ley, D. ; Ludden, C. ; Niamir, L. ; Nicholls, Z. ; Some, S. ; Szopa, S. ; Trewin, B. ; Wijst, K.-I. v. d. ; Winter, G. ; Witting, M. ; Birt, A. ; Ha, M. Climate Change 2023: Synthesis Report. Contribution of Working Groups I, II and III to the Sixth Assessment Report of the Intergovernmental Panel on Climate Change (IPCC); Core Writing Team, Lee, H. , Romero, J. , Eds.; IPCC: Geneva, Switzerland, 2023.

[2] Pauleta S. R., Carepo M. S. P., Moura I. (2019). Source and Reduction of Nitrous Oxide. Coord. Chem. Rev..

[3] Cant N. W., Chambers D. C., Liu I. O. Y. (2011). The
Reduction
of ^15^N^14^NO by CO and by H_2_ over Rh/SiO_2_: A Test of a Mechanistic Proposal. J. Catal..

[4] Bermejo J., Ortega-Lepe I., Santos L. L., Rendón N., López-Serrano J., Álvarez E., Suárez A. (2024). Nitrous Oxide
Activation by Picoline-Derived Ni–CNP Hydrides. Chem. Commun..

[5] Zeng R., Feller M., Ben-David Y., Milstein D. (2017). Hydrogenation and Hydrosilylation
of Nitrous Oxide Homogeneously Catalyzed by a Metal Complex. J. Am. Chem. Soc..

[6] Nappen S. T., Gamboa-Carballo J. J., Tschanen E., Ricatto F., Wörle M. D., Thomas A., Trincado M., Grützmacher H. (2025). Water Serving
as Cocatalyst for the Highly Efficient Homogeneously Catalyzed Conversion
of N_2_O/H_2_ Mixtures with Optimized Rhodium NHC
Complexes. Angew. Chem., Int. Ed..

[7] Dewick S. H., Hood T. M., Han Y., Huband S., Chaplin A. B. (2025). Rhodium-Catalysed
Hydrogenation of Nitrous Oxide. Catal. Sci.
Technol..

[8] Ortega-Lepe I., Sánchez P., Santos L. L., Lara P., Rendón N., López-Serrano J., Salazar-Pereda V., Álvarez E., Paneque M., Suárez A. (2022). Catalytic
Nitrous Oxide Reduction with H_2_ Mediated by Pincer Ir Complexes. Inorg. Chem..

[9] Ardon Y., Malakar S., Keller T. M., Gau M. R., Goldberg K. I. (2025). Acid Catalyst
Is Required for Hydrogenolysis of a Late
Metal Hydroxo Complex. J. Am. Chem. Soc..

[10] Hidalgo N., Moreno J. J., García-Rubio I., Campos J. (2022). Enhanced Dihydrogen
Activation by Mononuclear Iridium­(II) Compounds: A Mechanistic Study. Angew. Chem., Int. Ed..

[11] Pita-Milleiro A., Hidalgo N., Moreno J. J., Fernández I., Campos J. (2025). An Open-Shell Ir­(II)/Ir­(IV) Redox Couple Outperforms
an Ir­(I)/Ir­(III) Pair in Olefin Isomerization. Nat. Chem..

[12] Hood T. M., Dewick S. H., John A., Tidey J. P., Macpherson J. V., Bjornsson R., Richards E., Krämer T., Chaplin A. B. (2026). Solvent-Dependent Carbon-to-Metal Hydrogen Atom Transfer
Reactivity of a Square Planar Rhodium­(II) Alkynyl Complex. Organometallics.

[13] Hood T. M., Ward A. C. C., Krämer T., Chaplin A. B. (2026). Shedding Light on the Copper-Catalysed Diboron(4) Reduction
of Nitrous Oxide. Catal. Sci. Technol..

[14] Menglet D., Bond A. M., Coutinho K., Dickson R. S., Lazarev G. G., Olsen S. A., Pilbrow J. R. (1998). EPR, Electronic Spectra, and Electron
Transfer Properties of the 17 Electron Carbonylhydrotris­(Triphenylphosphine)­Rhodium­(II)
Cation. J. Am. Chem. Soc..

[15] Altus K. M., Furfari S. K., Goodall J. C., Gyton M. R., Heaton J. H., Johnson C. L., McKay A. I., Møller M. S., Pike S. D., Weller A. S. (2026). Solid-State Molecular
Organometallic
Chemistry (SMOM): A User Guide to in Crystallo Single-Crystal to Single-Crystal
Transformations Using Solid/Gas Methods. Dalton
Trans..

[16] Reid K. A., Powers D. C. (2021). Crystallo Organometallic
Chemistry. Chem. Commun..

[17] Hanson S. K., Heinekey D. M., Goldberg K. I. (2008). C–H
Bond Activation by Rhodium­(I) Phenoxide and Acetate Complexes: Mechanism
of H–D Exchange between Arenes and Water. Organometallics.

[18] Evans D. F. (1959). The Determination
of the Paramagnetic Susceptibility of Substances in Solution by Nuclear
Magnetic Resonance. J. Chem. Soc..

[19] Böttcher H., Klüfers P., Mies T., Mayer P. (2018). Synthesis and Molecular Structure
of *trans*-[RhCl_2_(*t*Bu_2_PPh)_2_]: A Rare Example of a Mononuclear Rhodium­(II)
Complex. Z. für Anorg. Allg. Chem..

[20] Feller M., Ben-Ari E., Gupta T., Shimon L. J. W., Leitus G., Diskin-Posner Y., Weiner L., Milstein D. (2007). Mononuclear Rh­(II)
PNP-Type Complexes. Structure and Reactivity. Inorg. Chem..

[21] Altus K. M., Shi Y., Probst P., Heaton J. H., Gyton M. R., Lari L., Buchmeiser M. R., Dyer P. W., Weller A. S. (2025). Room Temperature
Ethene to Propene (ETP) Tandem Catalysis Using Single Crystalline
Solid-State Molecular Pre-Catalysts. Angew.
Chem., Int. Ed..

[22] Wang L.-Q., Li T., Ma H.-H. (2021). Explosion
Behaviors of Hydrogen-Nitrous Oxide Mixtures at Reduced Initial Pressures. Process Saf. Environ. Prot..

[23] f Pregosin, P. S. NMR in Organometallic Chemistry; Wiley, 2012; pp 251–254;.

