# Supplementary material for: In Crystallo Hydrogenation of Nitrous Oxide Using a Rhodium(II) Hydride Complex
Source: Inorg Chem. 2026 Sep 11;65(38):22233–8. doi: 10.1021/acs.inorgchem.6c04272 (PMC13625954; doi:10.1021/acs.inorgchem.6c04272)
Supplement: Supplementary file 1 [file ic6c04272_si_001.pdf]

Supporting information for:

In crystallo hydrogenation of nitrous oxide using a rhodium(II) hydride complex

Thomas M. Hood,[a] Sophie H. Dewick,[a] Jie Liu,[b] Emma Richards,*[,c] and Adrian B. Chaplin*[,a]

[a] *Department of Chemistry, University of Warwick, Gibbet Hill Road, Coventry, CV4 7AL, UK;*
E-mail: a.b.chaplin@warwick.ac.uk.

[b] *Department of Physics, University of Warwick, Gibbet Hill Road, Coventry, CV4 7AL, UK.*

[c] *School of Chemistry, Cardiff University, Translational Research Hub, Cardiff CF24 4HQ, UK;*
E-mail: richardse10@cardiff.ac.uk.

Table of contents

1 General methods S2
2 Oxidation of [Rh(PNP-*t*Bu)H] with Fc[BAr$^F_4$] in solution S3
3 Preparation and characterisation of [Rh(PNP-*t*Bu)(CH_3)][BAr$^F_4$] 4 ..... S6
3.1 Solution stability and characterisation by NMR spectroscopy S7
3.2 Characterisation by EPR spectroscopy S11
3.3 Characterisation by SCXRD S11
3.4 Characterisation by PXRD S12
4 Preparation and characterisation of [Rh(PNP-*t*Bu)H][BAr$^F_4$] 1 ..... S13
4.1 Solution stability and characterisation by NMR spectroscopy S14
4.2 Characterisation by EPR spectroscopy S19
4.3 Characterisation by SCXRD S19
5 Preparation and characterisation of [Rh(PNP-*t*Bu)(OH)][BAr$^F_4$] 2 ..... S21
5.1 Solution stability and characterisation by NMR spectroscopy S22
5.2 Characterisation by EPR spectroscopy S24
5.3 Characterisation by SCXRD S24
6 Solid-state reaction of [Rh(PNP-*t*Bu)(OH)][BAr$^F_4$] 2 with H_2/D_2 ..... S26
6.1 Monitored by ATR-IR spectroscopy and GC-TCD S26
6.2 Monitored by NMR spectroscopy S27
6.3 Monitored by EPR spectroscopy S29
6.4 Monitored by SCXRD S31
7 Catalysis S34
8 Crystallographic parameters S37
9 EPR spectroscopic parameters S38
10 References S38

1 General methods

All manipulations were performed under argon (N4.8, 1 atm) using Schlenk and glove box (< 0.5 ppm H_2O and O_2) techniques, unless otherwise stated. Room temperature (RT) is approximately 293 K. Glassware was oven dried at 150 °C overnight and flame-dried under vacuum prior to use. Molecular sieves were activated by heating at 300 °C *in vacuo* overnight. Anhydrous tetrahydrofuran (THF) and 2-methyltetrahydrofuran (MeTHF) were vacuum distilled from sodium/benzophenone, freeze−pump−thaw degassed, and stored over 3 Å molecular sieves. Anhydrous hexane was degassed by sparging with argon and stored over 3 Å molecular sieves. 1,2-Difluorobenzene was purchased from Alfa Aesar (batch number 10245306) pre-dried over Al_2O_3, distilled from calcium hydride, freeze−pump−thaw degassed, and dried over 3 Å molecular sieves twice.[1] CD_2Cl_2 was freeze−pump−thaw degassed and dried over 3 Å molecular sieves. [Rh(PNP-*t*Bu)H],[2] Fc[BAr^F_4],[3] [Rh(PNP-*t*Bu)(CH_3)],[4] [Rh(COD)$_2$][BAr^F_4],[5] PNP-*t*Bu[6] and [Rh(PNP-*t*Bu)L][BAr^F_4] (L = H_2, CO, H_2O)[7] were prepared according to published procedures. Dihydrogen (N4.0) was dried by passage through a stainless-steel column of activated 3 Å molecular sieves. Chemically pure grade CO (N3.0) and research grade N_2O (N4.7) were purchased from BOC and used as supplied. NMR spectra were recorded on Bruker spectrometers under argon at 298 K unless otherwise stated. Chemical shifts are quoted in ppm and coupling constants in Hz. Virtual coupling constants are reported as the separation between the first and third lines.[8] NMR spectra in proteo solvents were recorded using an internal capillary of C_6D_6. EPR measurements were recorded on a Bruker EMX spectrometer utilizing an ER 072 magnet/ER 081 power supply combination (maximum field 0.65 T), an ER4102ST resonator, operating at 100 kHz field modulation, 0.3 mT modulation depth and 10 mW microwave power. Simulations of all EPR spectra were performed using garlic or pepper functions within the Easyspin toolbox.[9] ATR-IR spectra were recorded under dinitrogen using a Bruker ALPHA FT-IR spectrometer in a glove box. Single crystal X-ray diffraction (SCXRD) and powder X-ray diffraction (PXRD) data were collected on a Rigaku Oxford Diffraction XtaLAB Synergy (Dualflex) diffractometer equipped with a HyPix-Arc 100 hybrid pixel array detector using Cu Kα radiation (λ = 1.54184 Å) and an Oxford Cryosystems Cobra Plus cryostat operated at 100 K. CrysAlisPro was used for data collection, data reduction and the application of absorption corrections.[10] Single crystal structures were solved using SHELXT and refined using SHELXL, through the Olex2 graphical interface.[11,12] Further details are discussed below and documented in the CIFs of the structures, which have been deposited with the Cambridge Crystallographic Data Centre under CCDC 2564403–2564408. PXRD data were refined using the Rietveld method employing Pearson VII shape functions, as implemented in the EXPO2014 program.[13,14,15] Analysis of samples by gas chromatography (GC) was carried out anaerobically using an Agilent technologies 7820A instrument fitted with a thermal conductivity detector (TCD) with a He makeup flow (250 °C, 5 mLmin^{-1} He flow) and manual injector, connected to a 3-way Swagelok valve. The sample was passed through an Agilent HP-PLOT Molesieve column (19095P-MS6) held at 50 °C for 5 min then ramped to 200 °C over 10 min. Under the conditions employed, calibration with pure gases gave retention times of 1.4 min (H_2, inverse peak), 2.0 min (N_2), 2.45 min (CH_4) and 11.0 min (N_2O). Microanalysis was performed were performed in duplicate at Elemental Microanalysis Ltd.

2 Oxidation of [Rh(PNP-*t*Bu)H] with Fc[BAr^F$_4$] in solution

A mixture of [Rh(PNP-*t*Bu)H] (2.5 mg, 5.01 µmol) and Fc[BAr^F$_4$] (5.3 mg, 5.05 µmol) was dissolved in DFB, THF or MeTHF (0.5 mL) within a J. Young valve NMR tube and the ensuing reaction was monitored by ^{1}H and ^{31}P{^{1}H} NMR spectroscopy at RT. Consumption of the rhodium(I) hydride was observed within 5 min alongside formation of [Rh(PNP-*t*Bu)H][BAr^F$_4$] **1** and significant diamagnetic decomposition products.

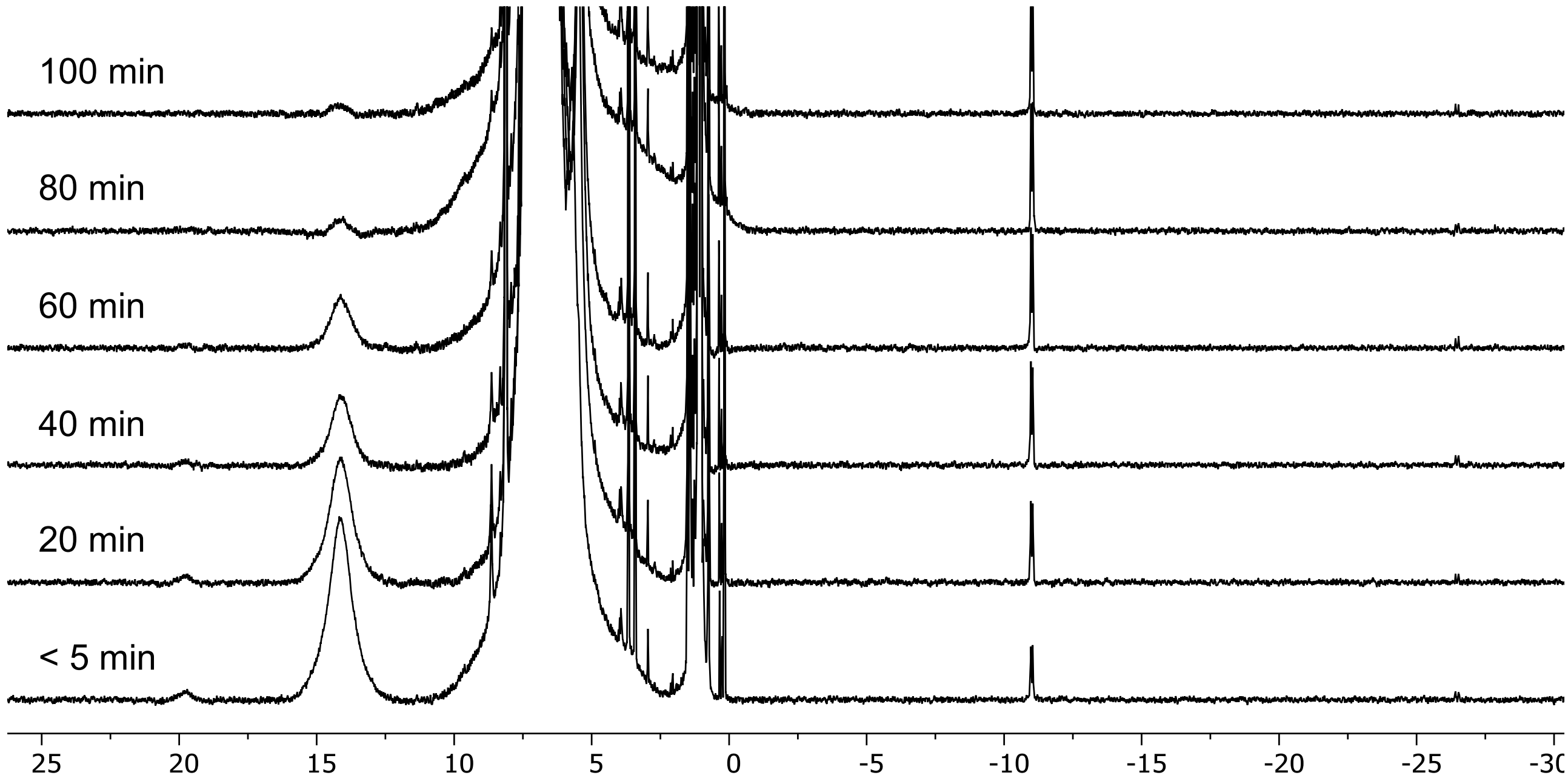

Figure S1. ^{1}H NMR spectra recorded following oxidation of [Rh(PNP-*t*Bu)H] with Fc[BAr^F$_4$] in DFB at RT (400 MHz). > 95% decomposition of **1** within 100 min.

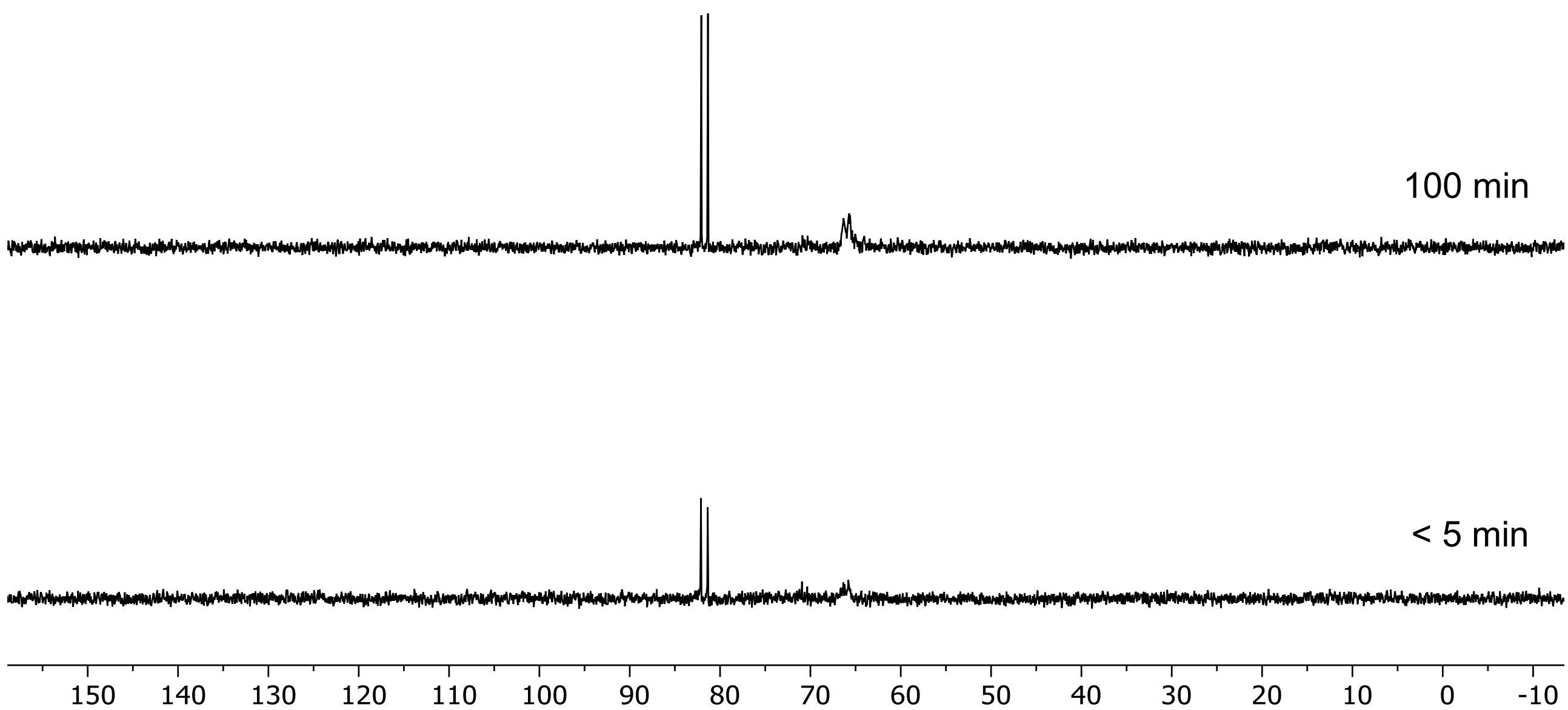

Figure S2. ^{31}P{^{1}H} NMR spectra recorded following oxidation of [Rh(PNP-*t*Bu)H] with Fc[BAr^F$_4$] in DFB at RT (162 MHz). After 100 min a 1:0.9 mixture of [Rh(PNP-*t*Bu)(H_2)][BAr^F$_4$] **3** (δ_{31P} 81.7, $^1J_{RhP}$ = 120 Hz; δ_{1H} –11.0, $^1J_{RhH}$ = 26 Hz, $^2J_{PH}$ = 6 Hz) and [Rh(PNP-*t*Bu)H(2,3-$F_2C_6H_3$)][BAr^F$_4$] (δ_{31P} 66.0, $^1J_{RhP}$ = 99 Hz; δ_{1H} –26.5, $^1J_{RhH}$ = 41 Hz) was obtained.

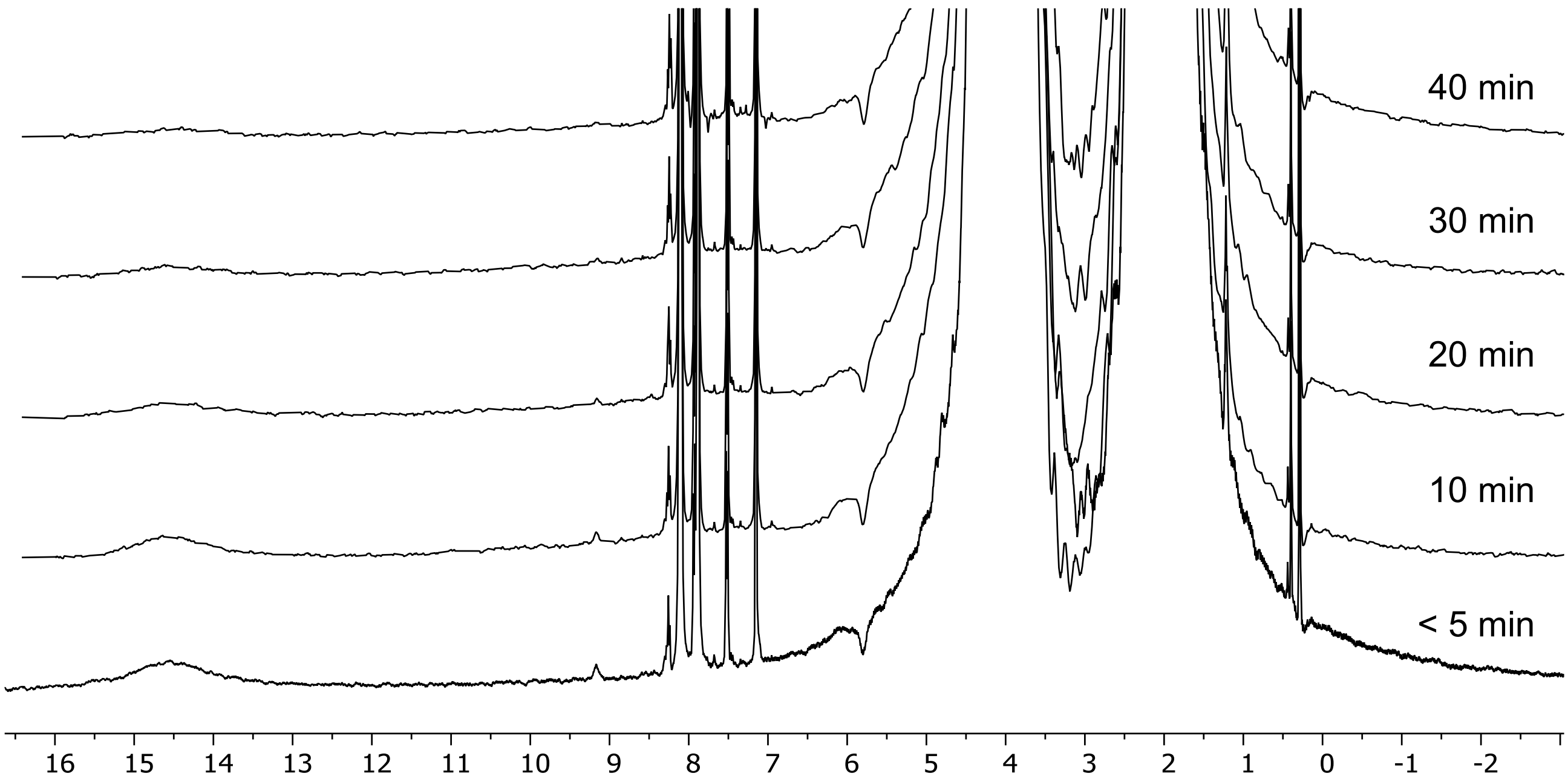

Figure S3. ^{1}H NMR spectra recorded following oxidation of [Rh(PNP-*t*Bu)H] with Fc[BAr$^{F}_{4}$] in THF at RT (400 MHz). > 95% decomposition of **1** within 30 min.

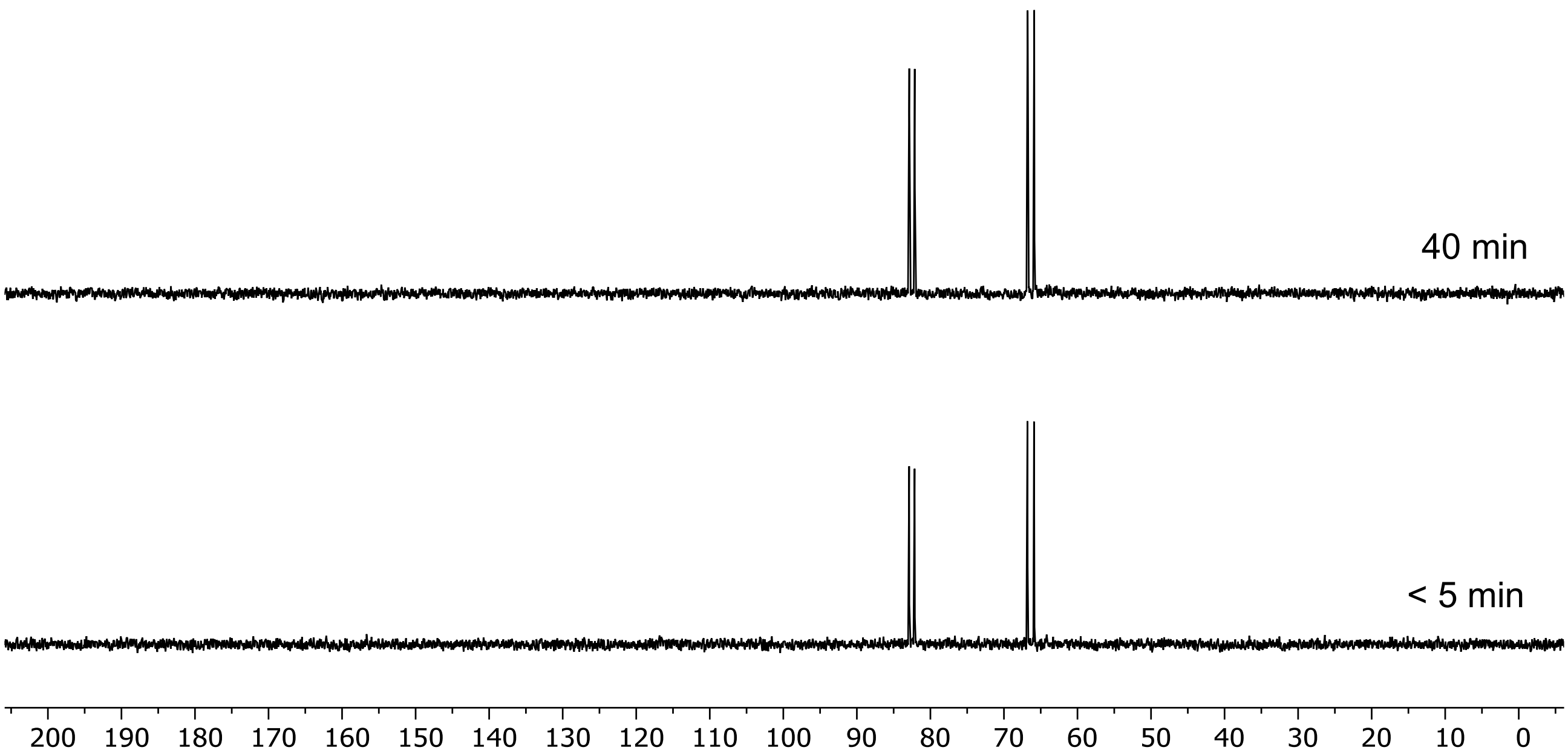

Figure S4. $^{31}P\{^{1}H\}$ NMR spectra recorded following oxidation of [Rh(PNP-*t*Bu)H] with Fc[BAr$^{F}_{4}$] in THF at RT (162 MHz). After 40 min a 1:1.4 mixture of [Rh(PNP-*t*Bu)(H_2)][BAr$^{F}_{4}$] **3** (δ_{31P} 82.5, $^{1}J_{RhP}$ = 120 Hz; δ_{1H} –10.5, $^{1}J_{RhH}$ = 29 Hz, $^{2}J_{PH}$ = 6 Hz) and [Rh(PNP-*t*Bu)(THF)][BAr$^{F}_{4}$] (δ_{31P} 66.4, $^{1}J_{RhP}$ = 145 Hz) was obtained. Polymerisation of THF was evident upon removal of the sample from the spectrometer.

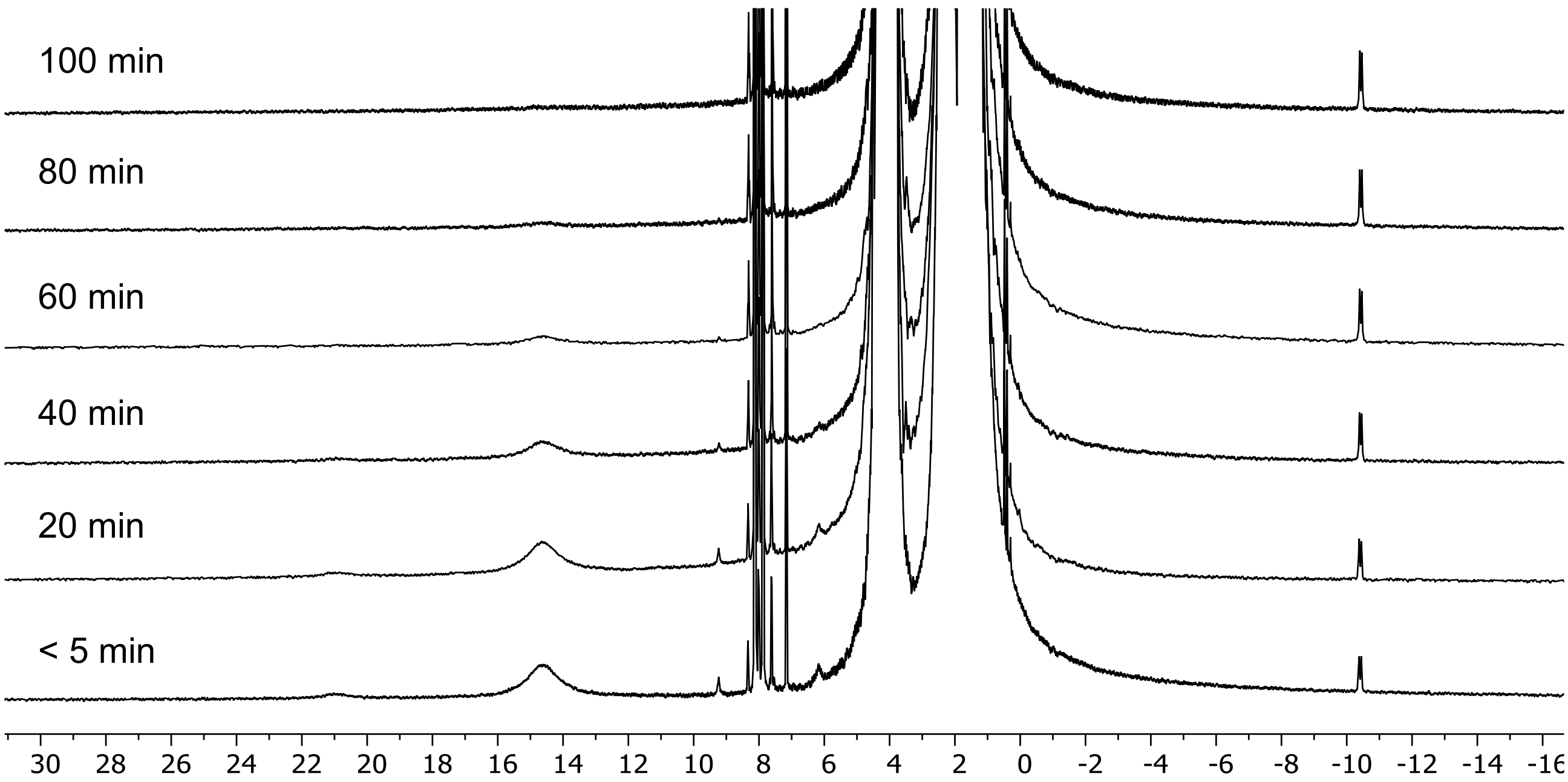

Figure S5. ^{1}H NMR spectra recorded following oxidation of [Rh(PNP-*t*Bu)H] with Fc[BAr$^{F}{}_{4}$] in MeTHF at RT (400 MHz). > 95% decomposition of **1** within 60 min.

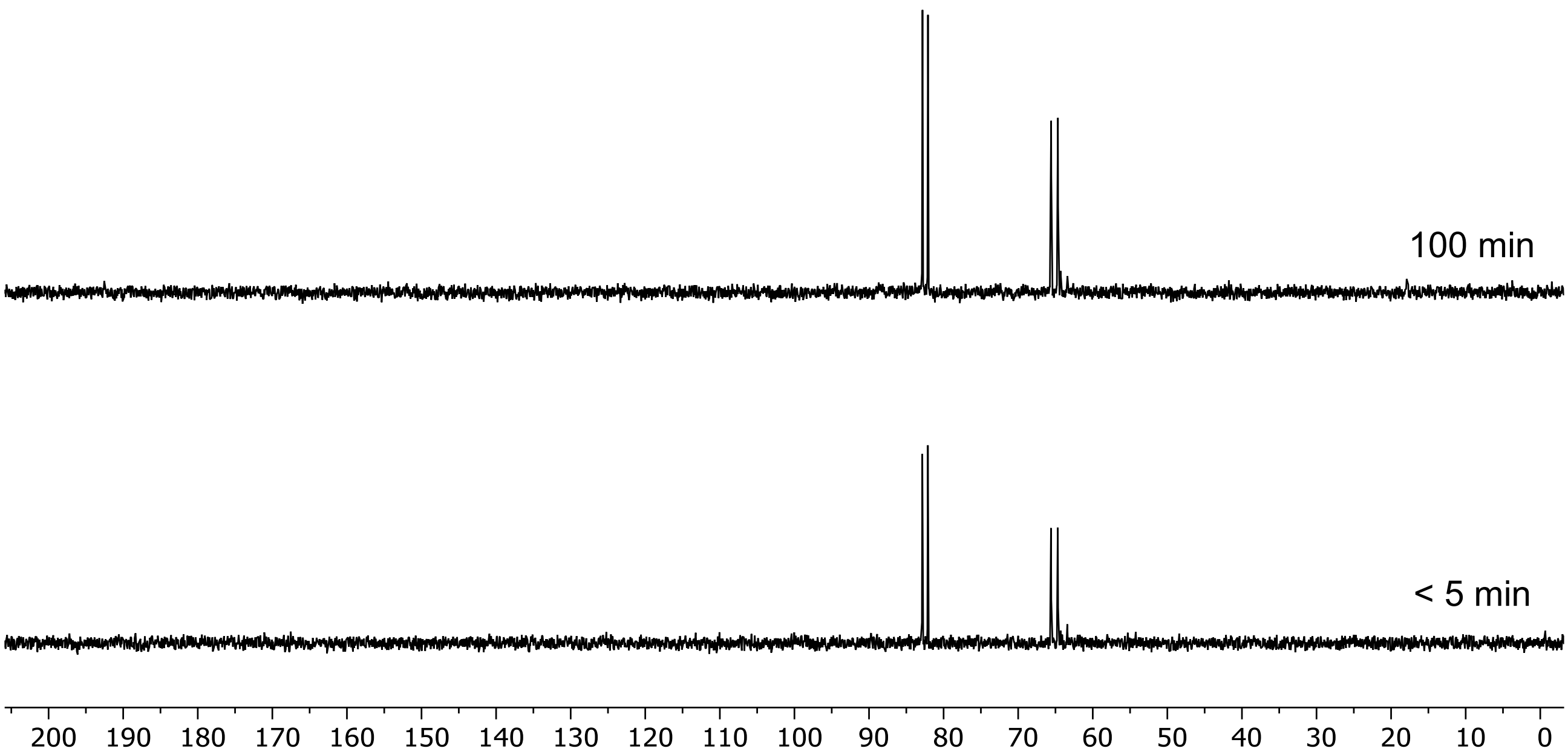

Figure S6. ^{31}P{^{1}H} NMR spectra recorded following oxidation of [Rh(PNP-*t*Bu)H] with Fc[BAr$^{F}{}_{4}$] in MeTHF at RT (162 MHz). After 100 min a 1:0.95 mixture of [Rh(PNP-*t*Bu)(H_2)][BAr$^{F}{}_{4}$] **3** (δ_{31P} 82.5, $^{1}J_{RhP}$ = 120 Hz; δ_{1H} –10.4, $^{1}J_{RhH}$ = 26 Hz, $^{2}J_{PH}$ = 6 Hz) and [Rh(PNP-*t*Bu)(MeTHF)][BAr$^{F}{}_{4}$] **5** (δ_{31P} 65.1, $^{1}J_{RhP}$ = 147 Hz; see below for independent synthesis) was obtained.

3 Preparation and characterisation of [Rh(PNP-*t*Bu)(CH_3)][BAr$^F_4$] 4

A solution of [Rh(PNP-*t*Bu)(CH_3)] (158 mg, 308 μmol) and Fc[BAr$^F_4$] (317 mg, 303 μmol) in DFB (5 mL) was stirred at RT for 10 min. The product was thereafter isolated as an analytically pure dark purple microcrystalline powder by addition of excess hexane (30 mL) and collected by filtration. The phase purity was confirmed by PXRD analysis. Yield: 354 mg (258 mmol, 84%). The sample for analysis by EPR spectroscopy was prepared by dissolution in MeTHF (250 μL, 10 mM) at RT, immediately frozen at 77 K and then flame-sealed under vacuum. The sample was kept at 77 K until it was loaded into the pre-cooled spectrometer at 115 K. Single crystals suitable for analysis by SCXRD were reproducibly grown from a 10 mM solution of the product in DFB layered with hexane at RT and collected after 16 h as purple/dark red blocks. A single batch was used for all subsequent single-crystal to single-crystal transformations.

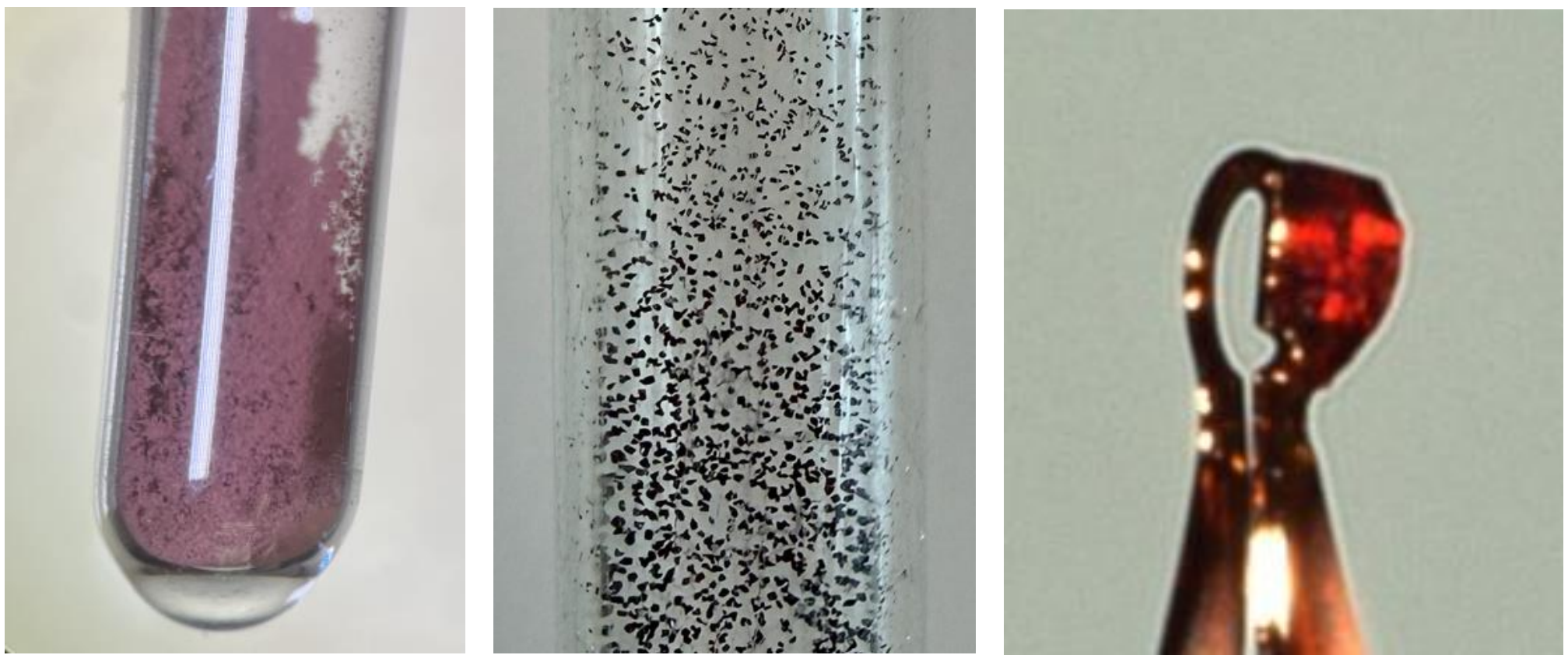

Figure S7. Samples of **4**: microcrystalline solid, single crystals, and single crystal.

^{1}H NMR (400 MHz, MeTHF, selected data): *δ* 32.59 (vbr, fwhm = 560 Hz, 4H, CH_2), 17.20 (vbr, fwhm = 430 Hz, 36H, *t*Bu), –60.24 (vbr, fwhm = 400 Hz, 3H, CH_3).

^{1}H NMR (400 MHz, DFB, selected data): *δ* 31.30 (vbr, fwhm = 490 Hz, 4H, CH_2), 16.55 (vbr, fwhm = 510 Hz, 36H, *t*Bu), 2.07 (br, fwhm = 53, 2H, py) –61.62 (vbr, fwhm = 1500 Hz, 3H, CH_3).

^{31}P{^{1}H} NMR (162 MHz, MeTHF): no resonances observed between *δ* −600 and +600.

^{31}P{^{1}H} NMR (162 MHz, DFB): no resonances observed between *δ* −600 and +600.

^{19}F{^{1}H} NMR (377 MHz, MeTHF): *δ* 62.9 (s).

^{11}B{^{1}H} NMR (128 MHz, MeTHF): *δ* –6.2 (s).

μ_{eff} (Evans' method, MeTHF): 1.64 μ_B

EPR (MeTHF glass, 115 K): **4**, **g** = [3.801 1.851 1.521], **A**(^{103}Rh) = [298 191 181] MHz; **4**·MeTHF, **g** = [2.496 2.336 1.971], **A**(^{103}Rh) = [121 131 128] MHz. **4**:**4**·MeTHF ratio = 1.0:1.0.

IR (ATR): featureless hydride region. Spectrum on page S27.

Anal. Calcd for $C_{56}H_{58}BF_{24}NP_2Rh$ (1376.71 gmol^{-1}): C, 48.86; H, 4.25; N, 1.02. Found: C, 48.88; H, 4.08; N, 1.16.

3.1 Solution stability and characterisation by NMR spectroscopy

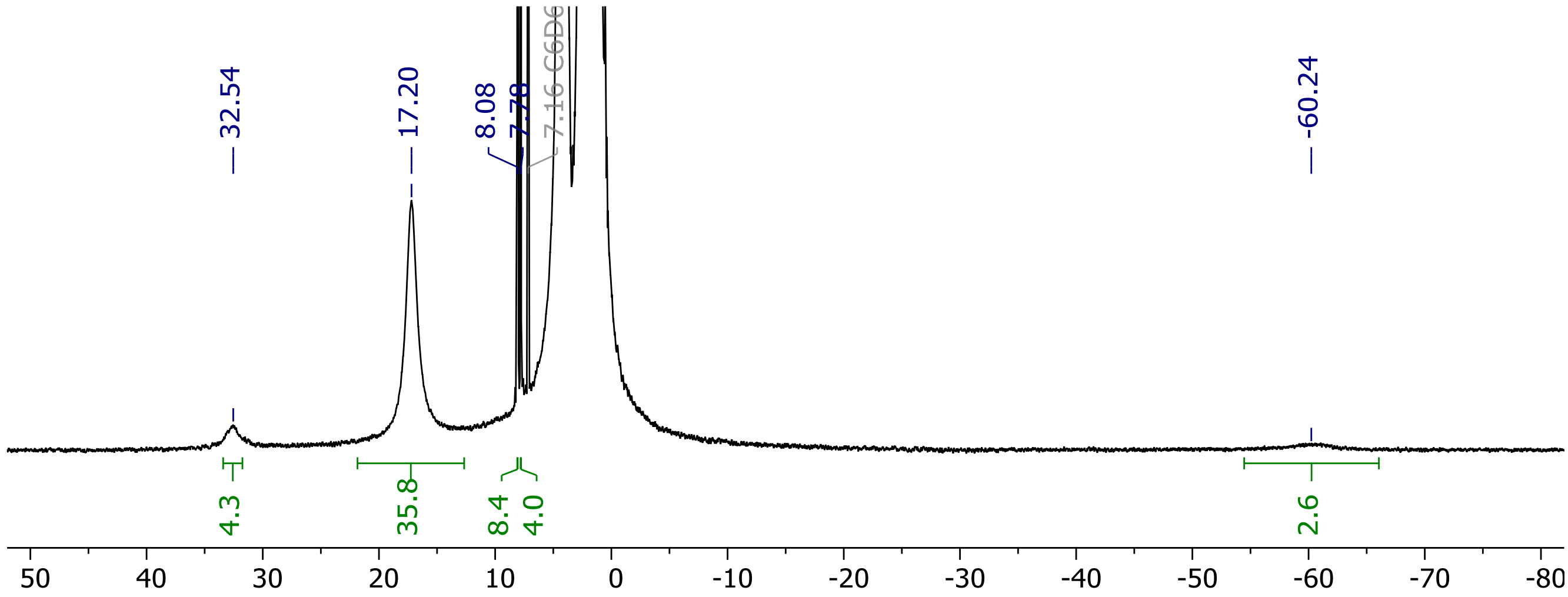

Figure S8. ^{1}H NMR spectrum of **4** (10 mM) recorded after < 5 min in MeTHF at RT (400 MHz).

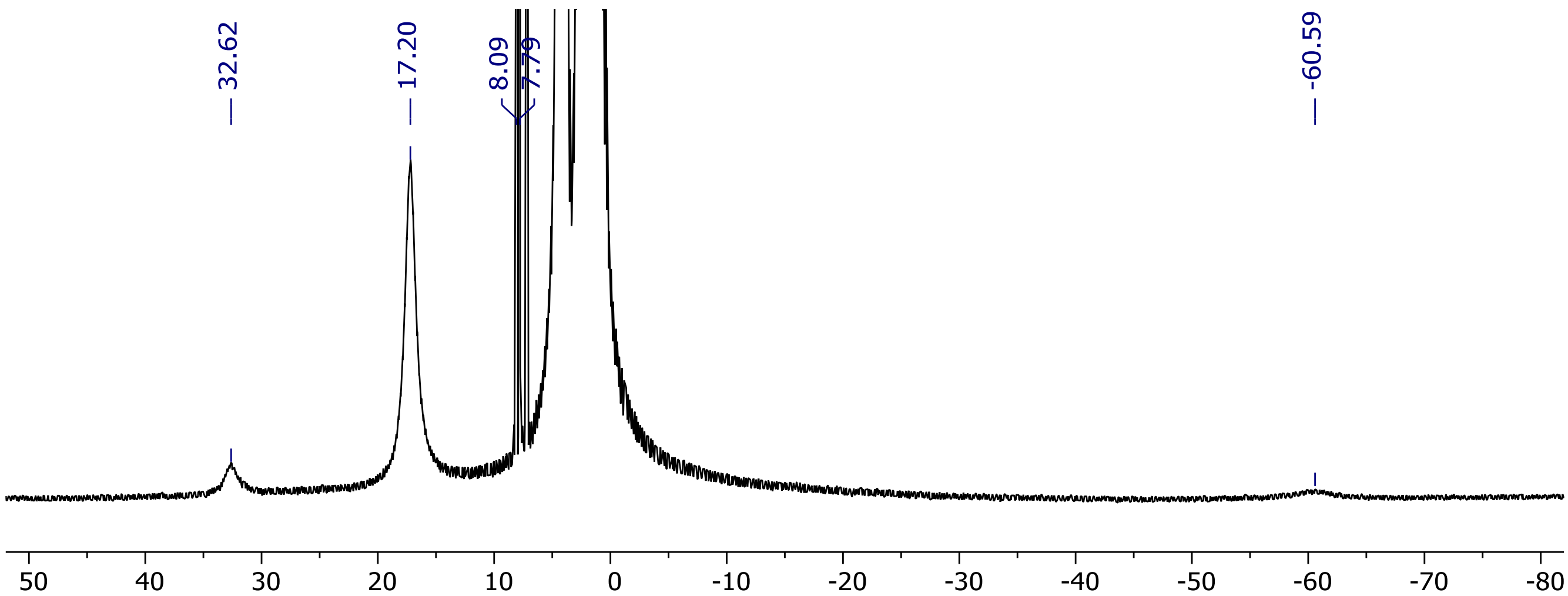

Figure S9. ^{1}H NMR spectrum of **4** (10 mM) recorded after 24 h in MeTHF at RT (400 MHz).
< 1% decomposition observed.

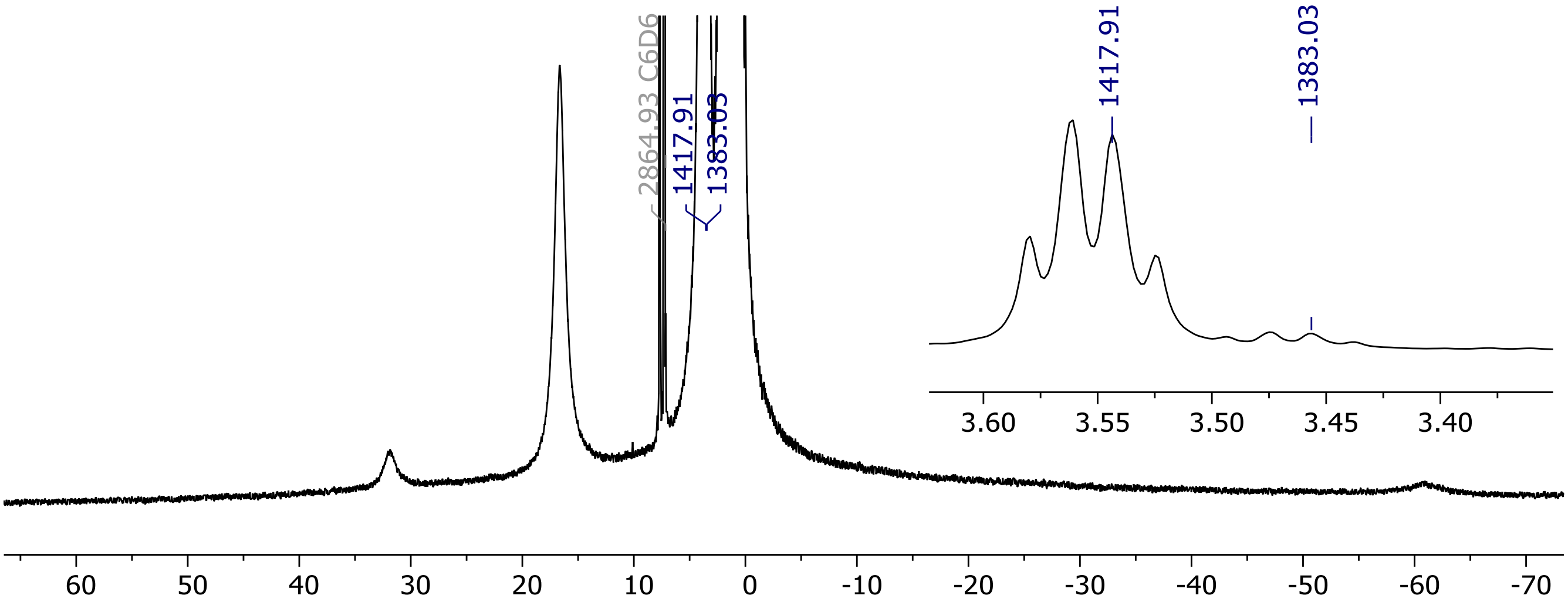

Figure S10. ^{1}H NMR spectrum of **4** (6.8 mg, 4.94 μmol) in a 4:1 v/v solution of MeTHF/C_6D_6 (450 μL) with an internal capillary of MeTHF/C_6D_6 (400 MHz, peaks in Hz). MeTHF paramagnetic shift = 35 Hz.

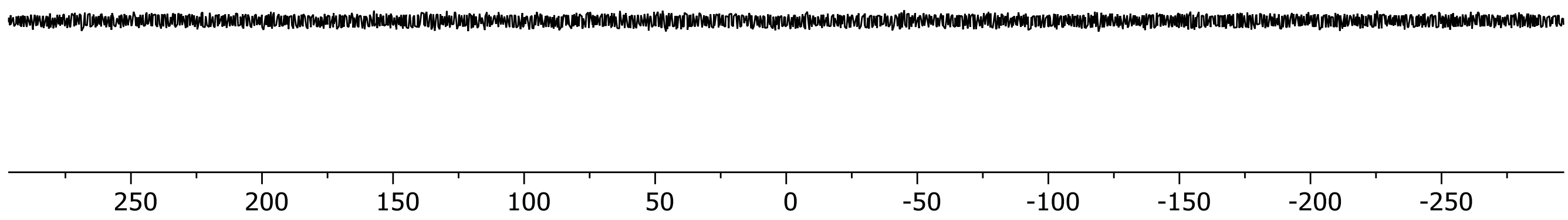

Figure S11. $^{31}P\{^{1}H\}$ NMR spectrum of **4** (10 mM) recorded after < 5 min in MeTHF at RT (162 MHz).

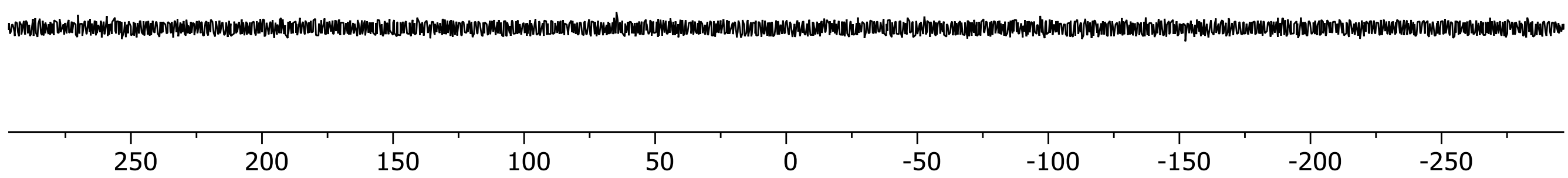

Figure S12. $^{31}P\{^{1}H\}$ NMR spectrum of **4** (10 mM) recorded after 24 h in MeTHF at RT (162 MHz). < 1% decomposition observed.

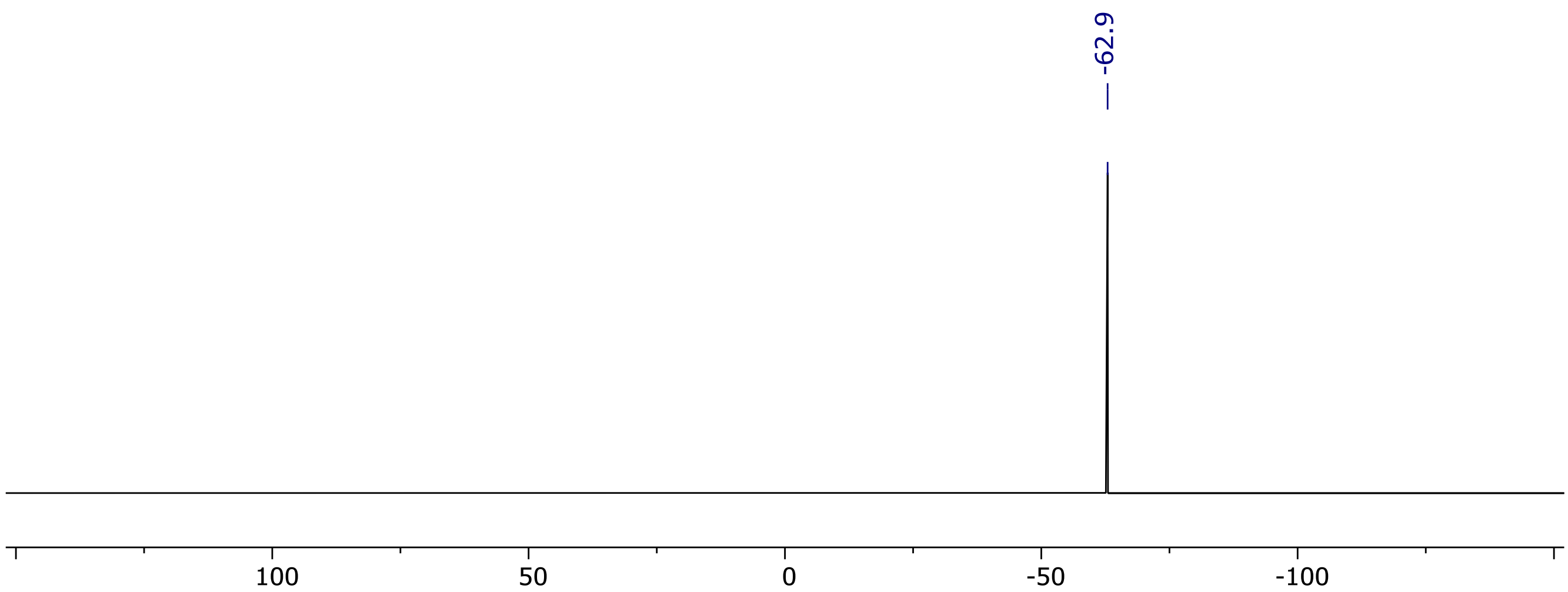

Figure S13. $^{19}F\{^{1}H\}$ NMR spectrum of **4** (10 mM) recorded after < 5 min in MeTHF at RT (377 MHz).

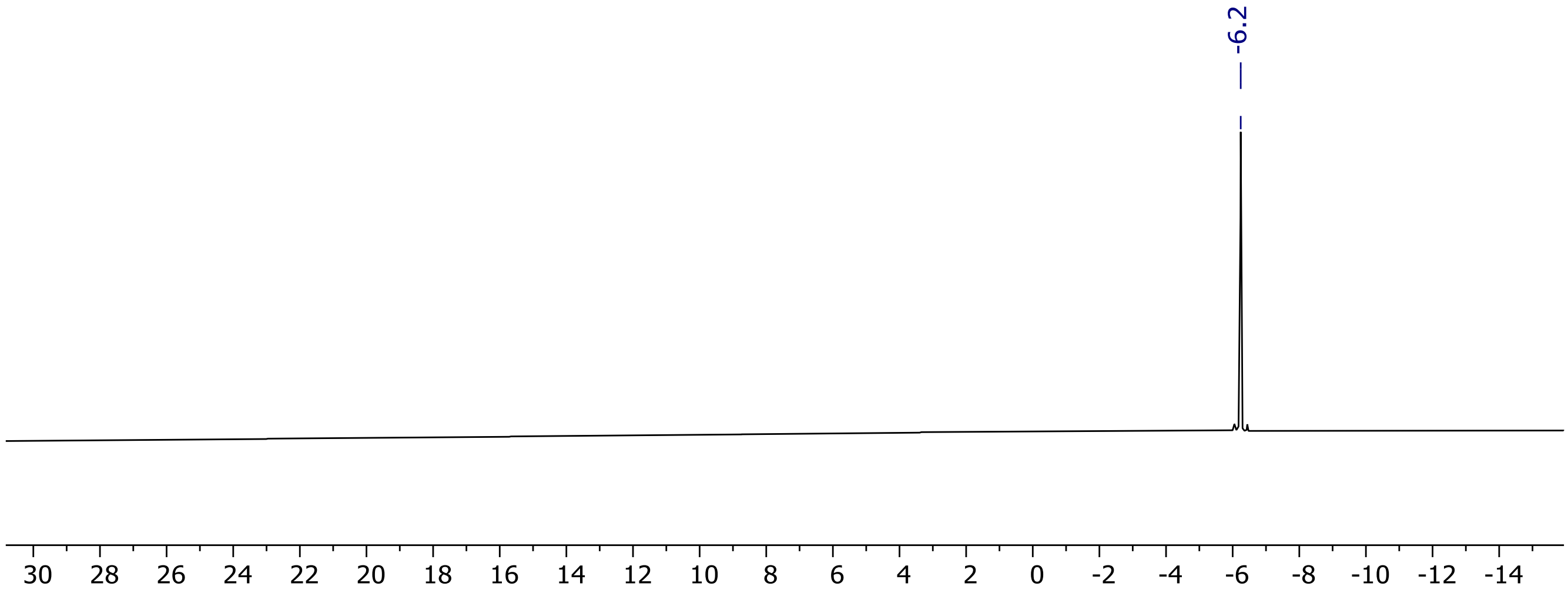

Figure S14. $^{11}B\{^1H\}$ NMR spectrum of **4** (10 mM) recorded after < 5 min in MeTHF at RT (128 MHz).

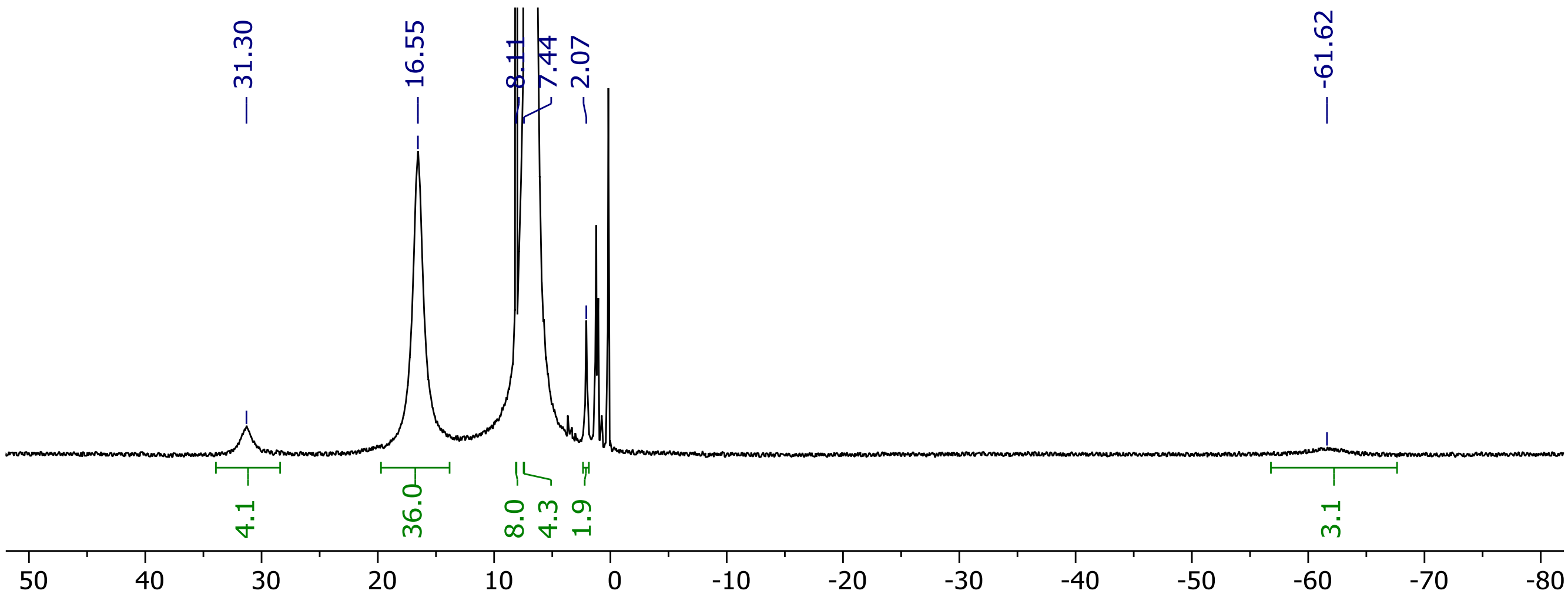

Figure S15. ^1H NMR spectrum of **4** (10 mM) recorded after < 5 min in DFB at RT (400 MHz).

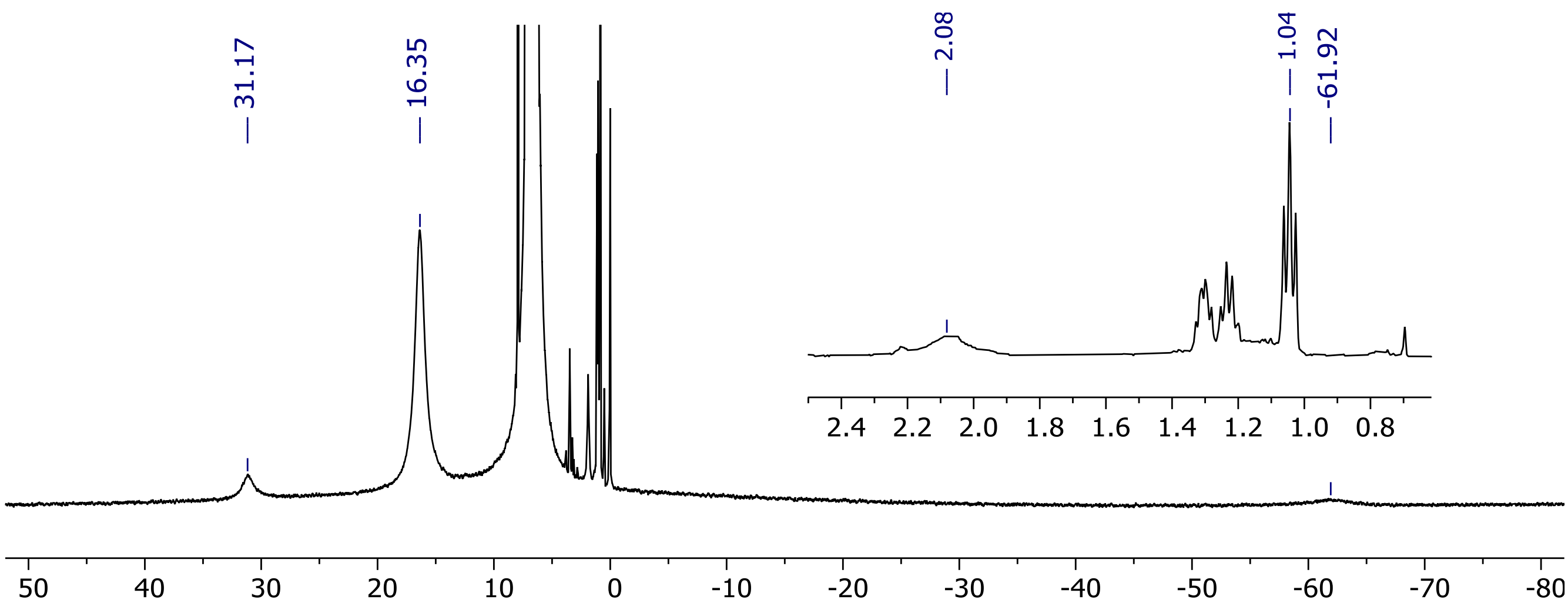

Figure S16. ^1H NMR spectrum of **4** (10 mM) recorded after 24 h in DFB at RT (400 MHz).

< 10% decomposition observed.

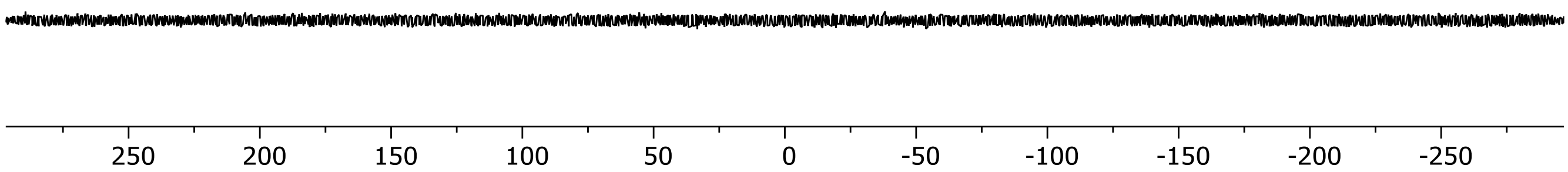

Figure S17. $^{31}P\{^{1}H\}$ NMR spectrum of **4** (10 mM) recorded after < 5 min in DFB at RT (162 MHz).

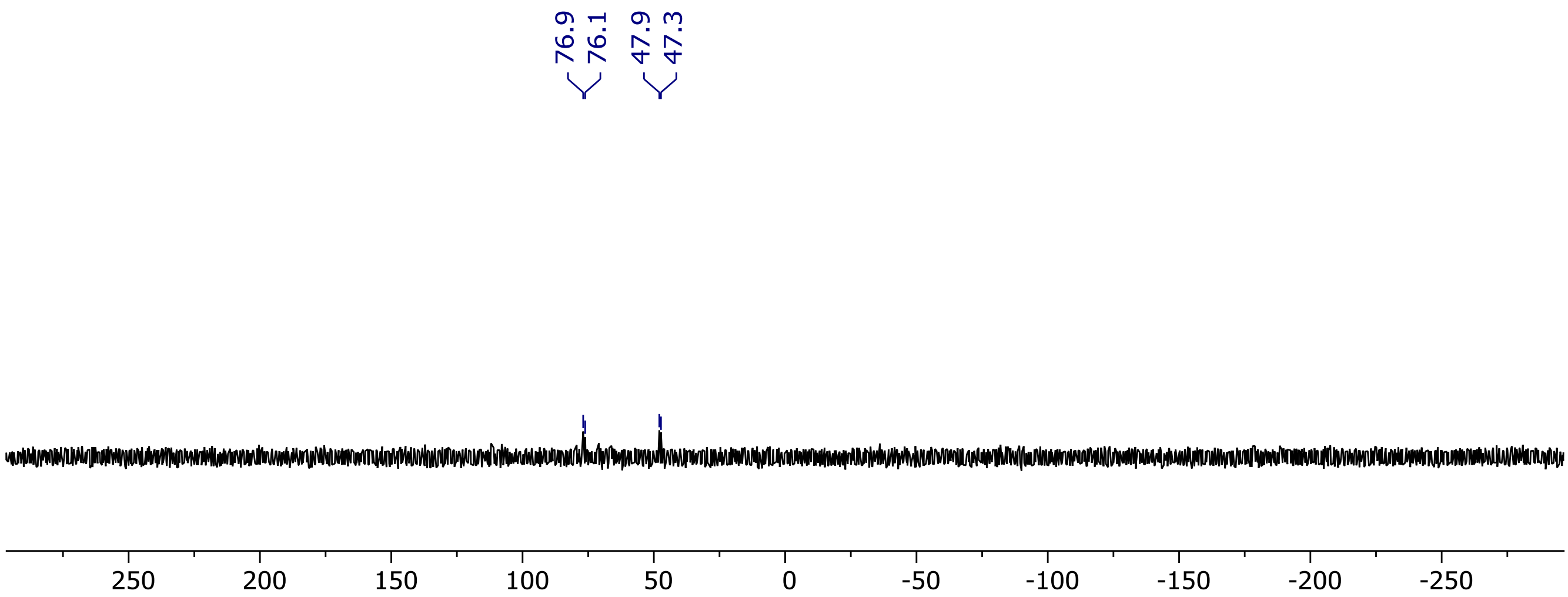

Figure S18. $^{31}P\{^{1}H\}$ NMR spectrum of **4** (10 mM) recorded after 24 h in DFB at RT (162 MHz).
< 10% decomposition observed.

3.2 Characterisation by EPR spectroscopy

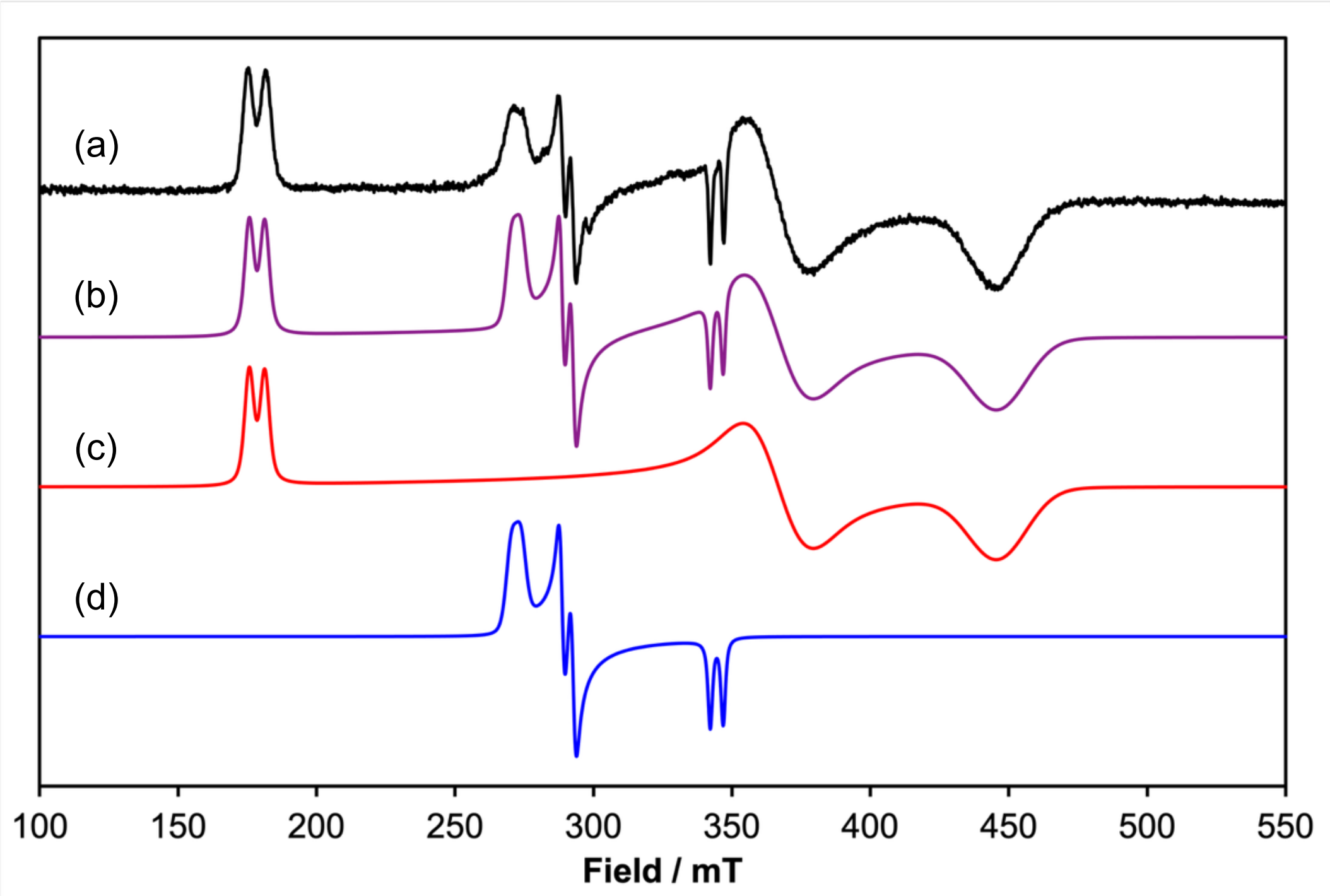

Figure S19. CW X-band EPR spectrum of **4** recorded in MeTHF frozen glass at 115 K. (a) experimental spectrum, (b) full simulated spectrum, and simulated spectra of **4** (c) and **4**·MeTHF (d). **4**:**4**·MeTHF ratio = 1.0:1.0.

3.3 Characterisation by SCXRD

The structure of **4** was refined full-matrix least squares on F^2. All non-hydrogen atoms were refined anisotropically. Hydrogen atoms were placed in calculated positions and refined using the riding model. There is no significant crystallographic disorder evident in the cation (Figure S20). Rotational disorder of the CF_3 groups of the anion was treated by modelling the fluorine atoms over two sites and restraining their geometry. Restraints to thermal parameters were applied to the fluorine atoms to maintain sensible values.

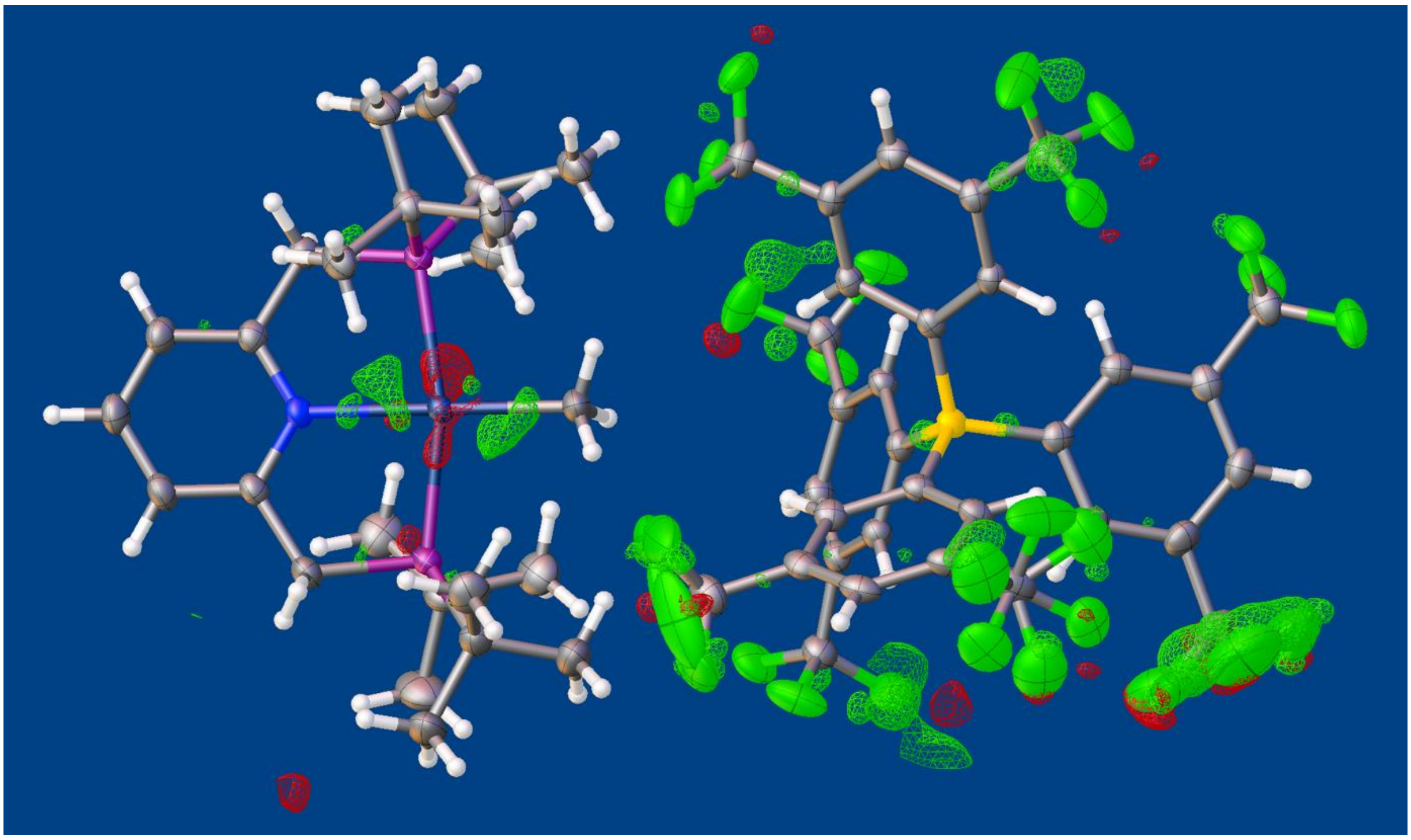

Figure S20. Difference map for the refined SCXRD structure of **4** showing residual density > 0.3 eÅ^{-3}.

3.4 Characterisation by PXRD

Phase purity of microcrystalline **4** was confirmed by Rietveld refinement of PXRD data collected at 100 K using the SCXRD structure determined at 100 K as the reference.

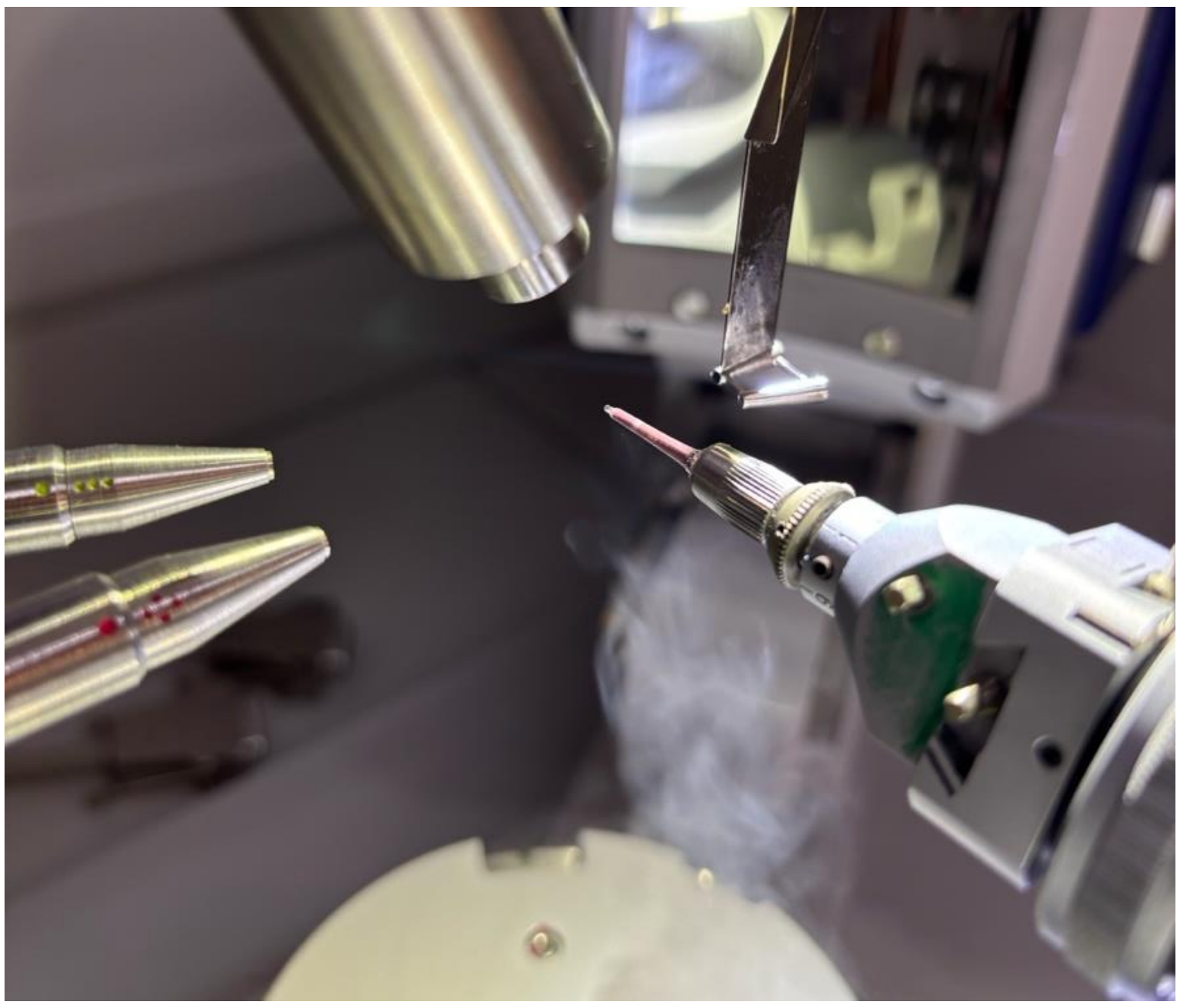

Figure S21. Collection of PXRD data for **4** using a sealed glass capillary at 100 K.

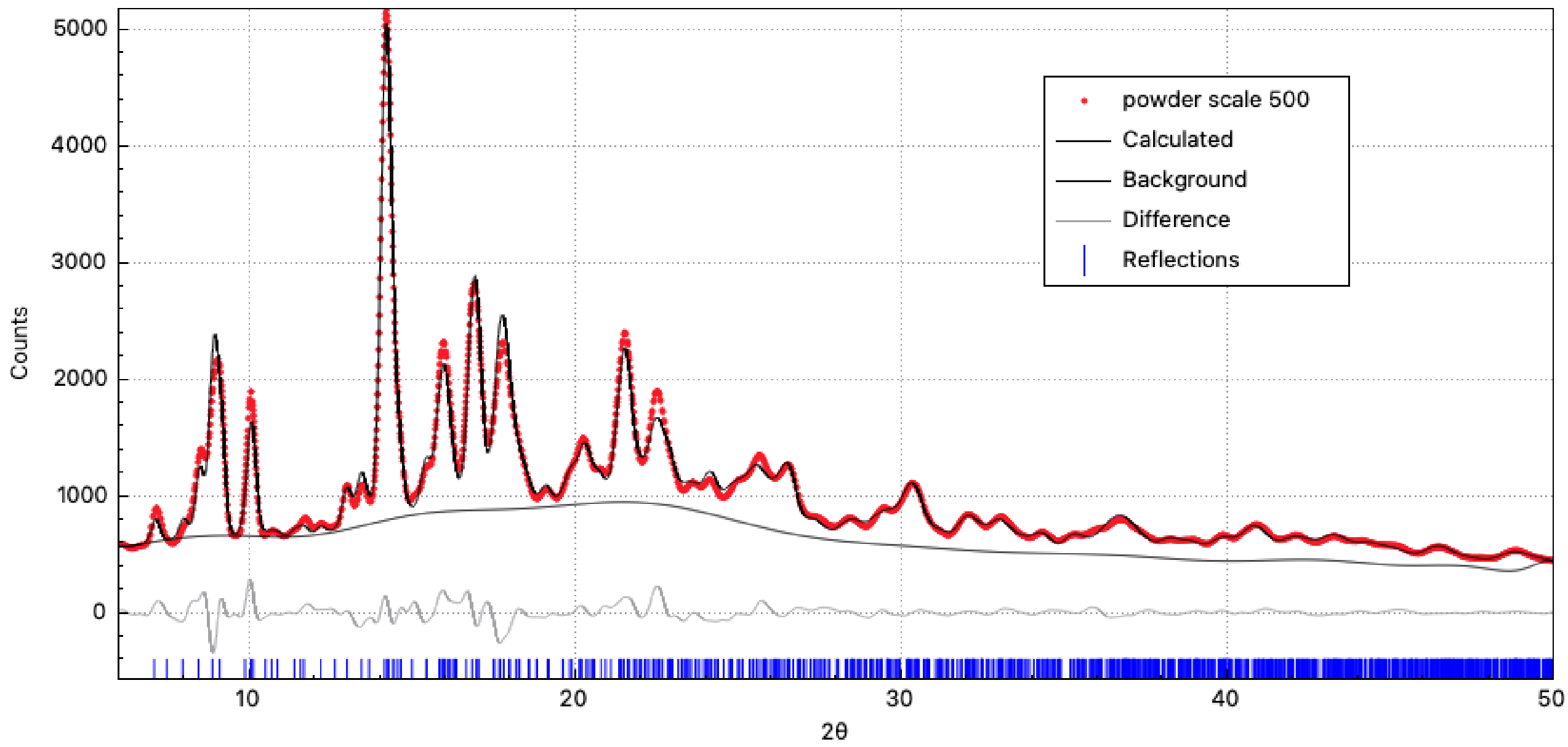

Figure S22. PXRD data for **4**. Refined Rietveld parameters:
R_p = 3.681 R_{wp} = 4.867 R_{exp} = 3.215 χ^2= 2.291.

4 Preparation and characterisation of [Rh(PNP-*t*Bu)H][BAr^F_4] 1

Method A: A J. Young valve NMR tube charged with microcrystalline **4** (6.9 mg, 5.01 µmol) was placed under H_2 (1 atm) for 1 h at RT. The headspace was analysed by GC-TCD, indicating the generation of CH_4, and the pastel pink powder was characterised by ATR-IR and NMR spectroscopies. The isotopologue [Rh(PNP-*t*Bu)D][BAr^F_4] d_1-**1** was prepared analogously using D_2 gas.

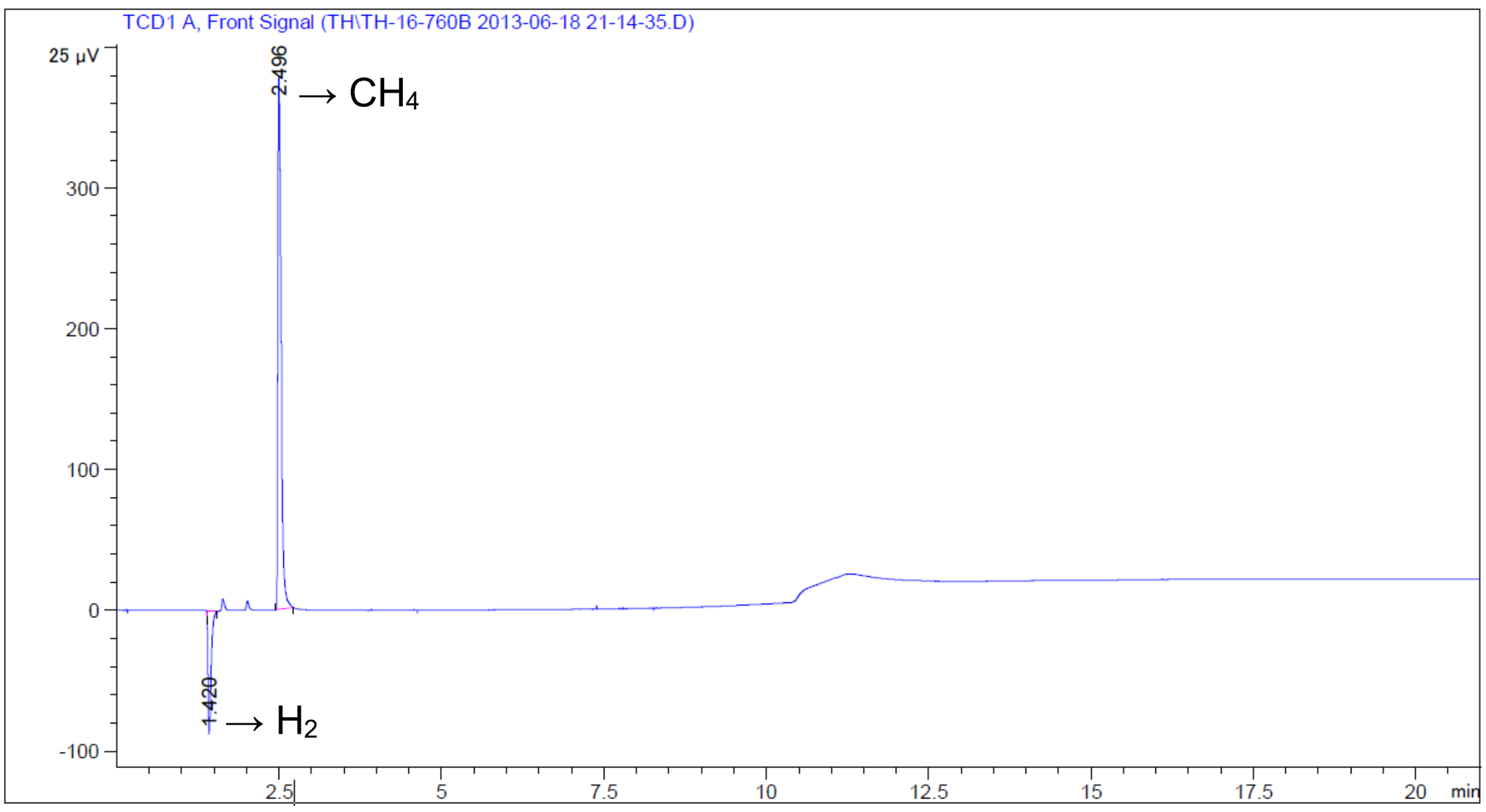

Figure S23. GC-TCD trace collected following the reaction of **4** with H_2.

Method B: A J. Young valve EPR tube charged with microcrystalline **4** (3.4 mg, 2.47 µmol) was placed under H_2 (1 atm) for 1 h at RT. The sample was placed under vacuum and MeTHF (250 µL) was added by vacuum transfer at 77 K. The product was dissolved in thawing MeTHF at 195 K to give a 10 mM

solution which was thereafter frozen at 77 K and flame-sealed under vacuum. The sample was kept at 77 K until it was loaded into the pre-cooled spectrometer at 115 K.

Method C: Single crystals of **4** were placed under H_2 (1 atm) for 2 h at RT. The resulting orange crystals were analysed by SCXRD at 100 K.

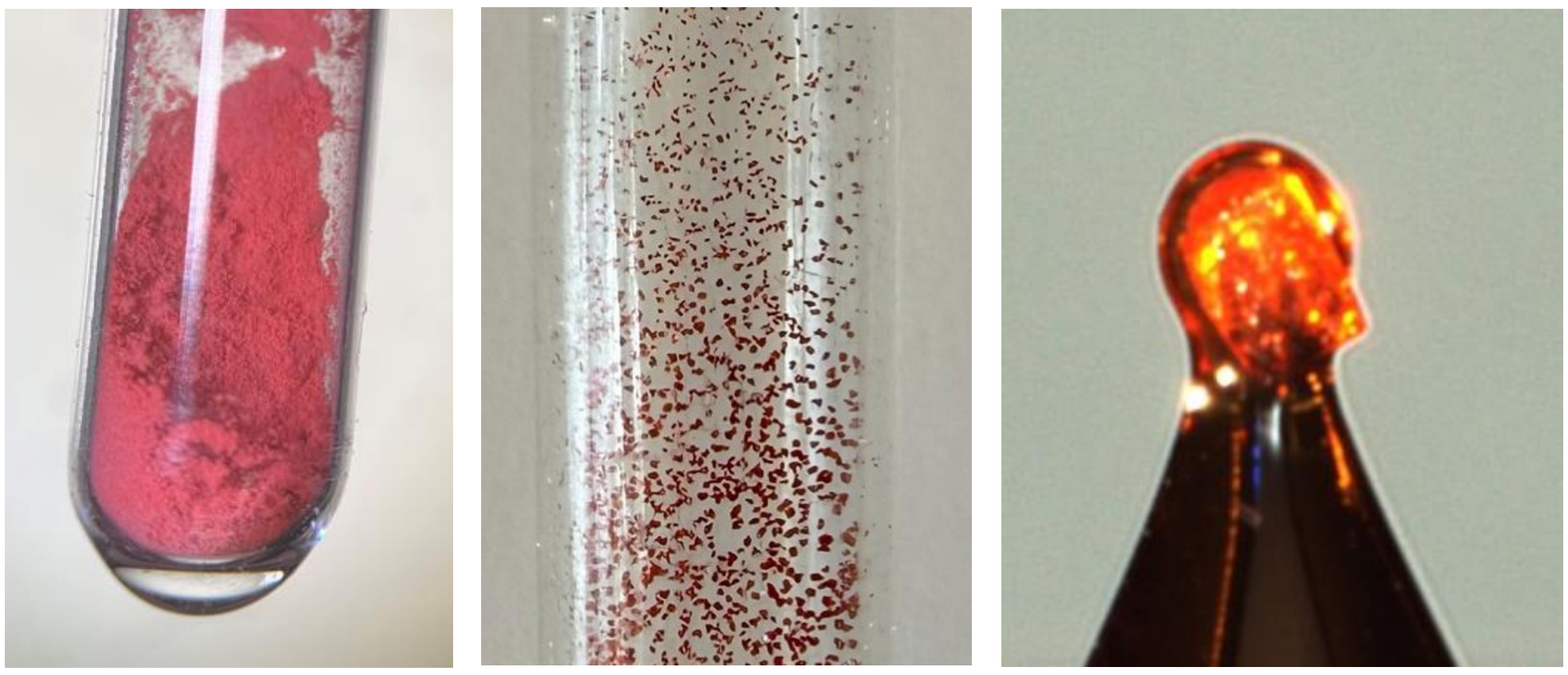

Figure S24. Samples of **1**: microcrystalline solid, single crystals, and single crystal.

^{1}H NMR (400 MHz, MeTHF, selected data): δ 20.95 (vbr, fwhm = 380 Hz, 4H, CH_2), 14.66 (vbr, fwhm = 150 Hz, 36H, *t*Bu), 9.29 (s, 1H, py), 6.23 (br, fwhm = 80 Hz, 2H, py).
^{31}P{^{1}H} NMR (162 MHz, MeTHF): no resonances observed between δ −600 and +600.
EPR (MeTHF glass, 115 K): **1**, **g** = [3.246 2.351 1.825], **A**(^{103}Rh) = [291 195 187] MHz; **1·**MeTHF; **g** = [2.446 2.336 1.975], **A**(^{103}Rh) = [133 139 126] MHz, **A**(^{1}H) = [0 0 40] MHz. **1**:**1·**MeTHF ratio = 1.0:1.3.
IR (ATR): v(Rh–H) = 1941 cm^{-1}. Spectrum on page S27.

4.1 Solution stability and characterisation by NMR spectroscopy

Isolated **1** was analysed in MeTHF (10 mM) by ^{1}H and ^{31}P NMR spectroscopy. The complex is sufficiently stable at RT to permit characterisation, with < 5% decomposition observed within 5 min. Upon standing at RT, > 95% decomposition was observed into a 1:0.75 mixture of [Rh(PNP-*t*Bu)(H_2)][BAr$^F_4$] **3** (δ_{31P} 82.5, $^{1}J_{RhP}$ = 120 Hz; δ_{1H} –10.4, $^{1}J_{RhH}$ = 26 Hz, $^{2}J_{PH}$ = 6 Hz) and a rhodium(I) complex assigned to [Rh(PNP-*t*Bu)(MeTHF)][BAr$^F_4$] **5** (δ_{31P} 65.1, $^{1}J_{RhP}$ = 147 Hz) within 90 min. The identity of the latter was thereafter substantiated by independent synthesis from [Rh(COD)$_2$][BAr$^F_4$] (11.8 mg, 9.98 µmol) and PNP-*t*Bu (4.0 mg, 10.1 µmol) in MeTHF (0.5 mL) at RT using a procedure we have previously developed for the synthesis of rhodium(I) complexes of the form [Rh(pincer)L]$^+$ (quantitative spectroscopic yield).[7, 16] Considerably slower decomposition of **1** was observed starting with a concentration of 5 mM. Although the paramagnetically broadened signals and use of a proto solvent make it difficult to study the kinetics quantitatively, the initial rates approximated by integration are consistent with the dominant decomposition pathway being bimolecular in nature (3.0×10^{-6} molL^{-1}s^{-1} vs 8.1×10^{-7} molL^{-1}s^{-1}). To interrogate the mechanism further, the decomposition of d_1-**1** (10 mM) was studied revealing a significant kinetic isotope effect, with an approximate initial rate of 1.2×10^{-6} molL^{-1}s^{-1} (KIE ~ 2.4) and > 95% decomposition within 230 min. The known isotopologues of **3** [Rh(PNP-*t*Bu)(D_2)][BAr$^F_4$] (δ_{31P} 82.4, $^{1}J_{RhP}$ = 120 Hz) and [Rh(PNP-

tBu)(HD)][BAr$^F_4$] (δ_{31P} 82.5, $^1J_{RhP}$ = 120 Hz; δ_{1H} –10.4, $^1J_{RhH}$ ~ $^1J_{DH}$ = 27 Hz, $^2J_{PH}$ = 6 Hz) were ultimately obtained in a 1:0.2 ratio alongside **5**.[17] The ~10% H-atom incorporation and KIE is consistent with primary decomposition of **1** occurring via a bimetallic pathway (viz. [Rh^{II}]–H + [Rh^{II}]–H → [Rh^{I}]–H_2 + [Rh^{I}]–MeTHF, 0% H-atom incorporation from d_1-**1**) with secondary decomposition by H-atom abstraction from the solvent (viz. [Rh^{II}]–H + MeTHF → [Rh^{I}]–H_2 + $C_5H_{10}O^\bullet$, 50% H-atom incorporation from d_1-**1**) although the relative rates are difficult to extrapolate to d_0-**1**.

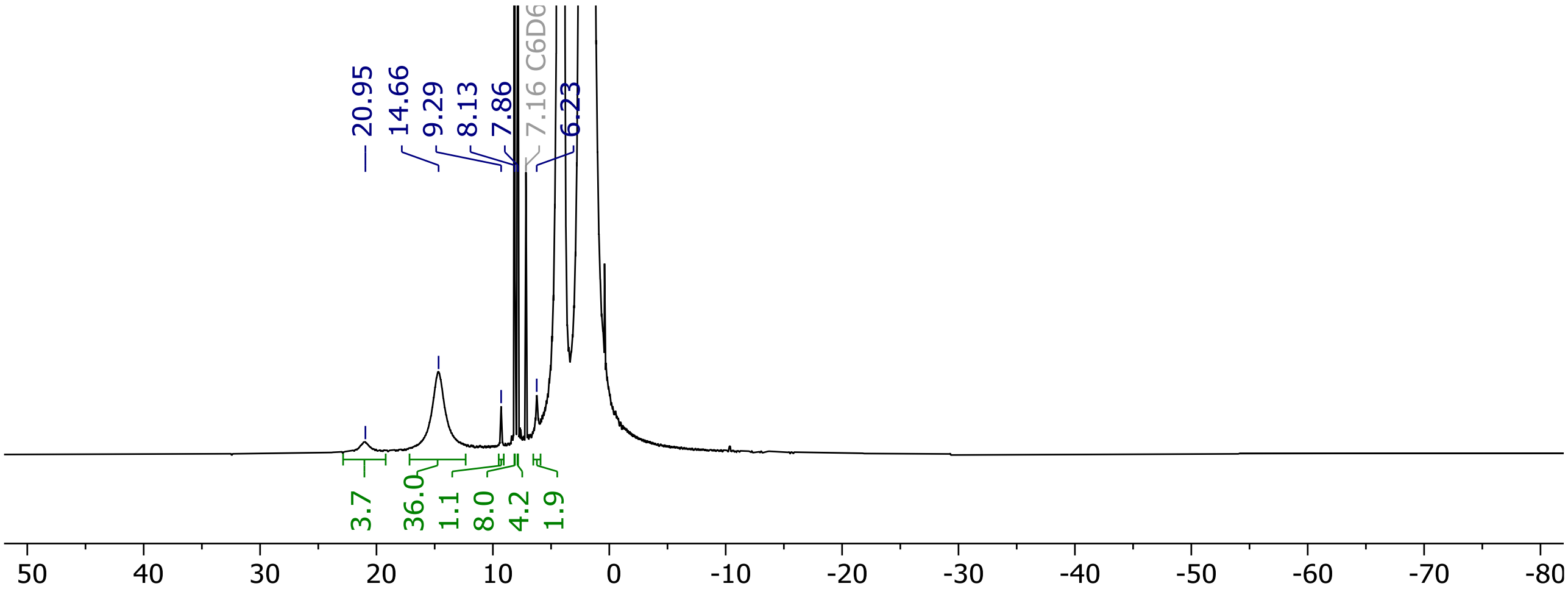

Figure S25. ^{1}H NMR spectrum of **1** (10 mM) recorded after < 5 min in MeTHF at RT (400 MHz).

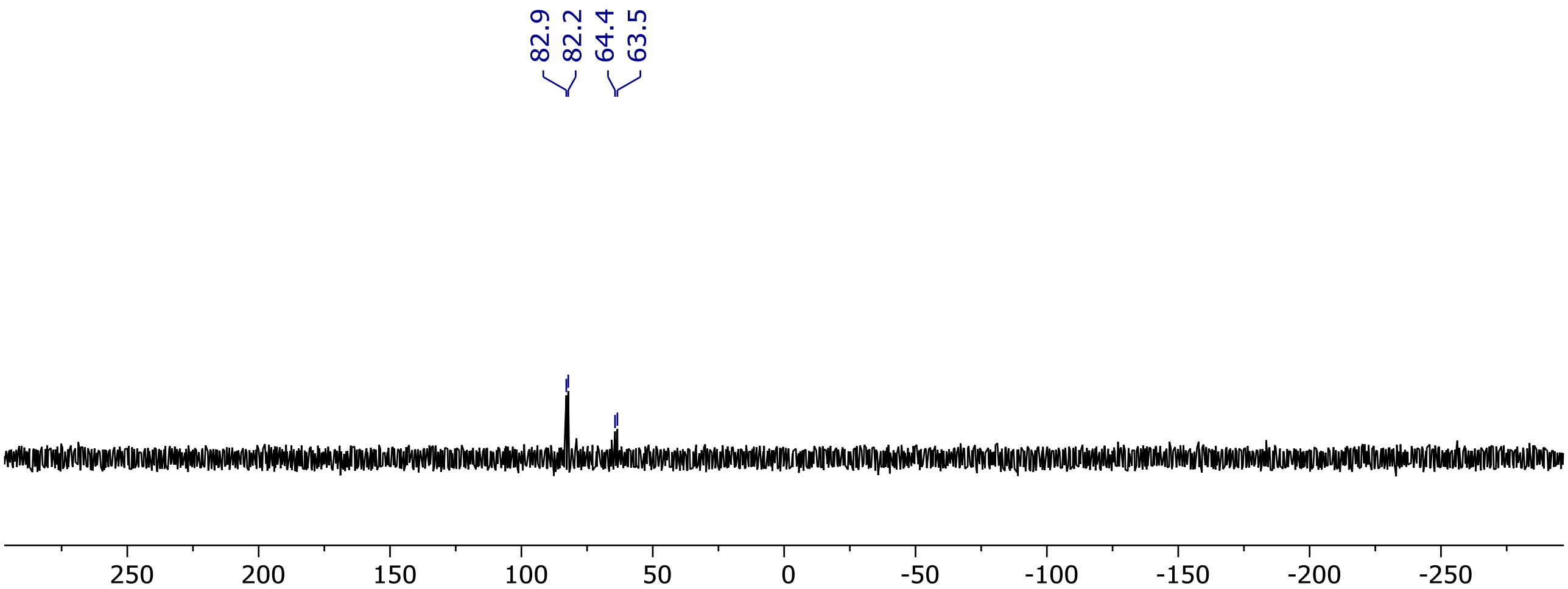

Figure S26. ^{31}P{^{1}H} NMR spectrum of **1** (10 mM) recorded after < 5 min in MeTHF at RT (162 MHz).

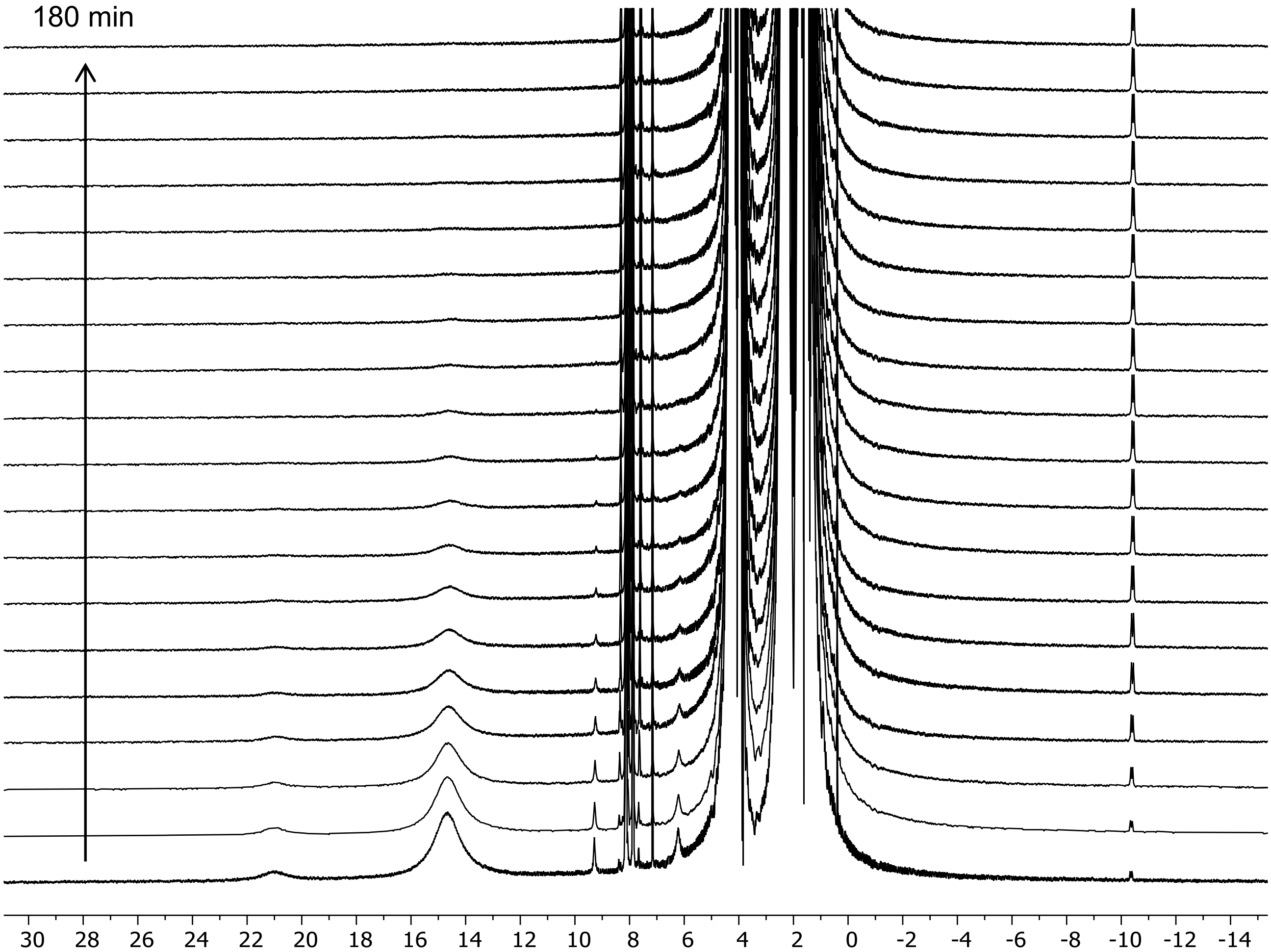

Figure S27. ^{1}H NMR spectra of **1** (10 mM) recorded over time in MeTHF at 298 K (10 min increments, 400 MHz). > 95% decomposition of **1** within 90 min.

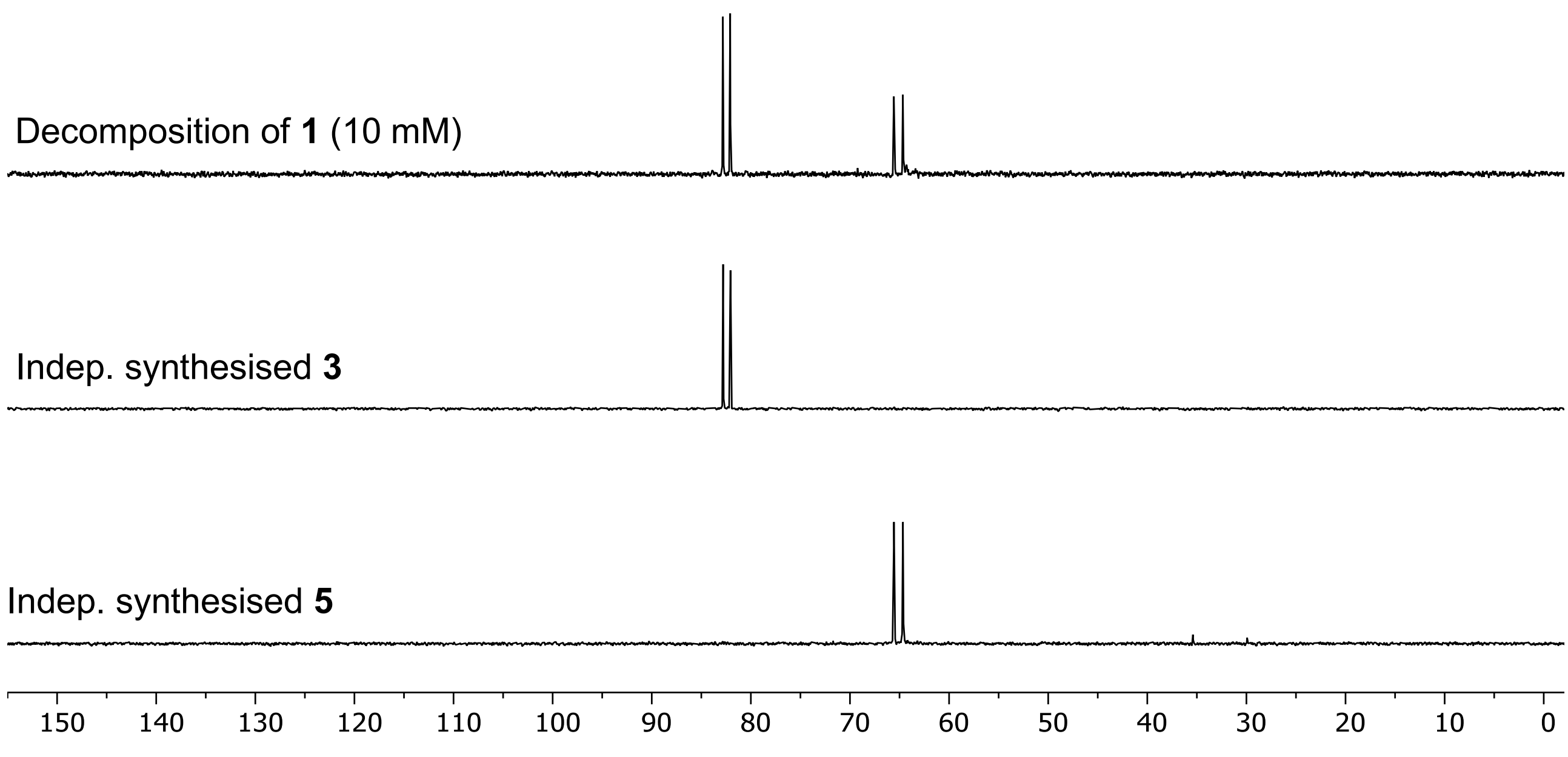

Figure S28. $^{31}P\{^{1}H\}$ NMR spectra of **1** (10 mM) recorded after 180 min in MeTHF at RT, independently synthesised **3** in MeTHF, and independently synthesised **5** in in MeTHF (162 MHz).

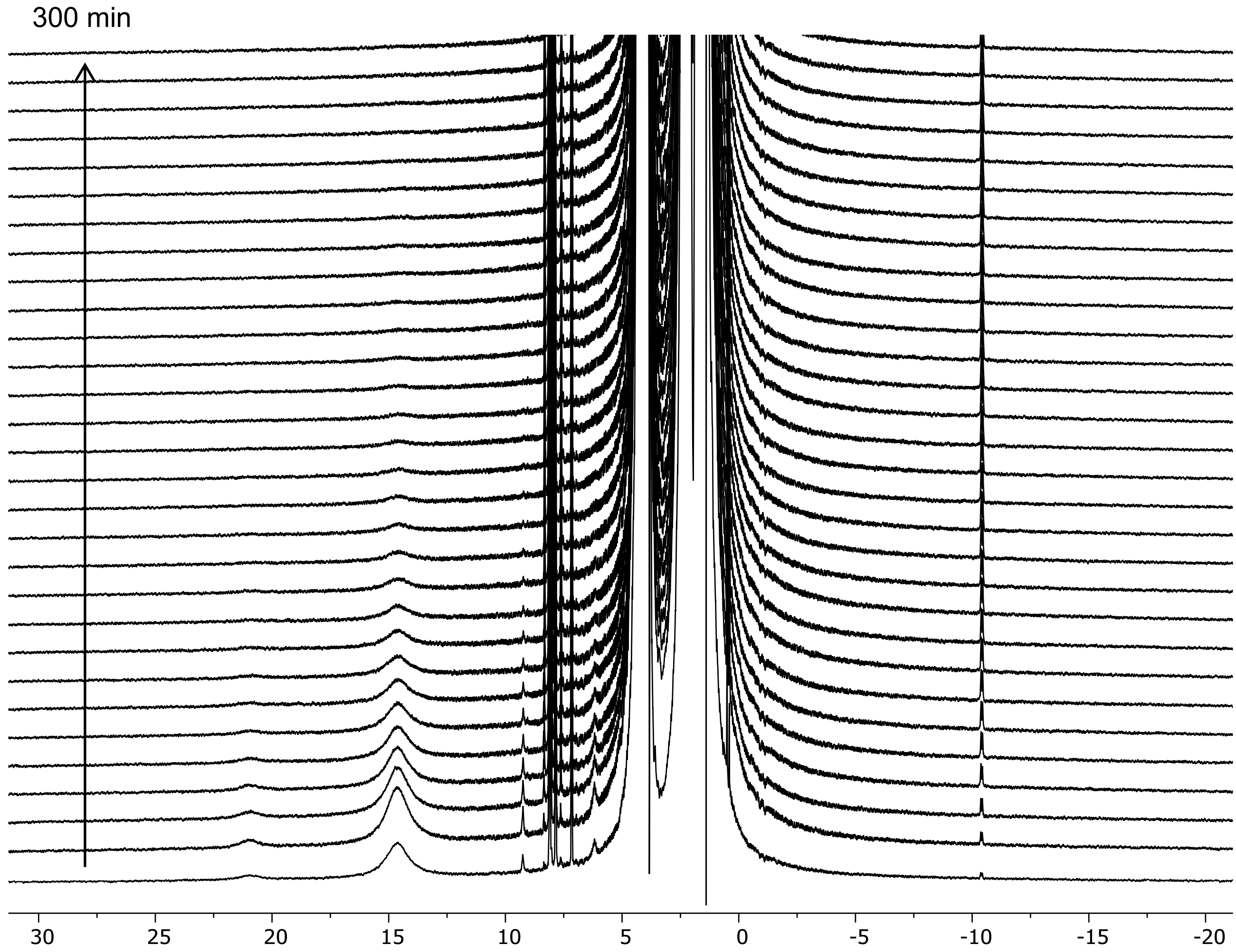

Figure S29. ^{1}H NMR spectra of **1** (5 mM) recorded over time in MeTHF at 298 K (10 min increments, 400 MHz). > 95% decomposition of **1** within 130 min.

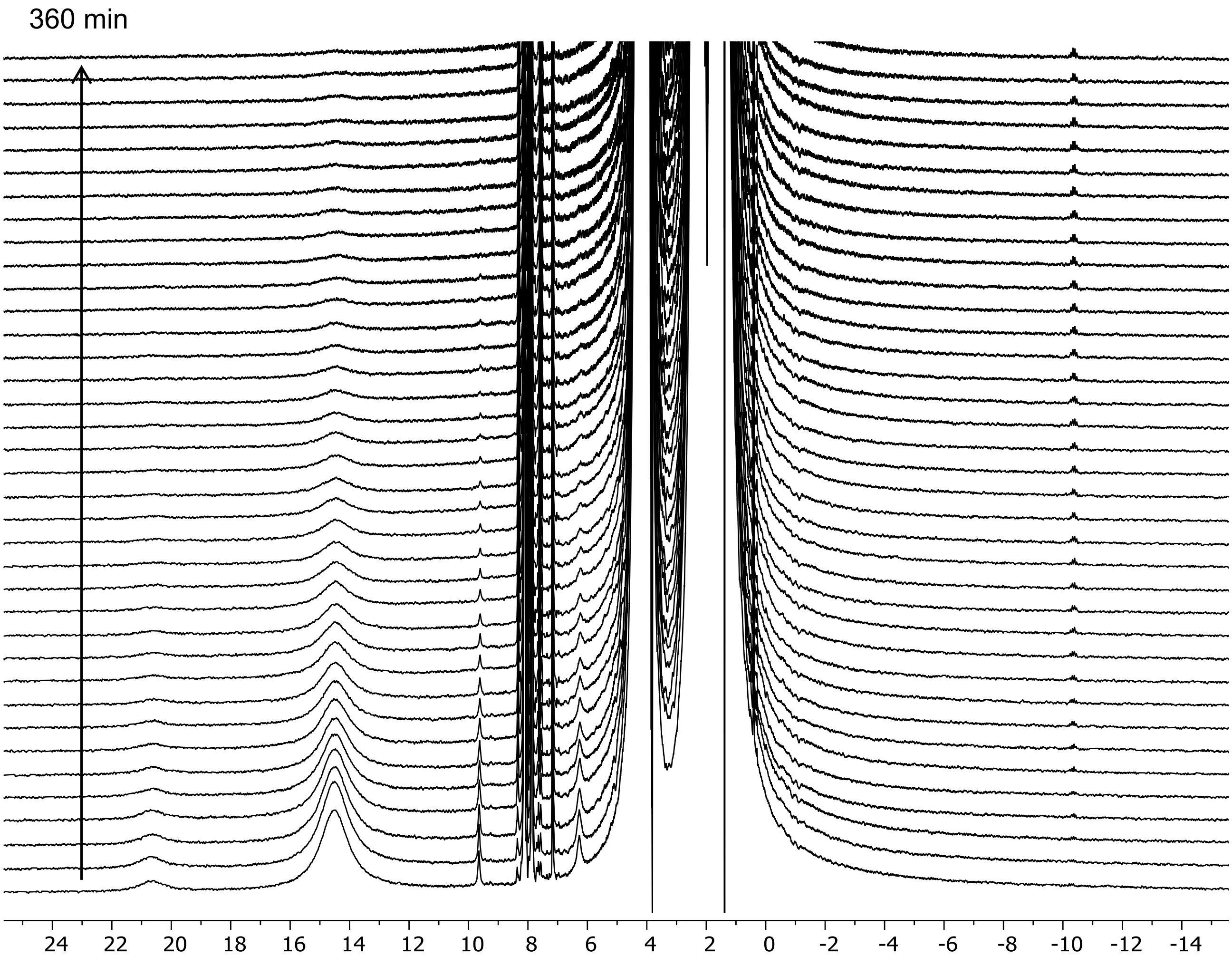

Figure S30. ^{1}H NMR spectra of d_1-**1** (10 mM) recorded over time in MeTHF at 298 K (10 min increments, 400 MHz). > 95% decomposition of d_1-**1** within 230 min.

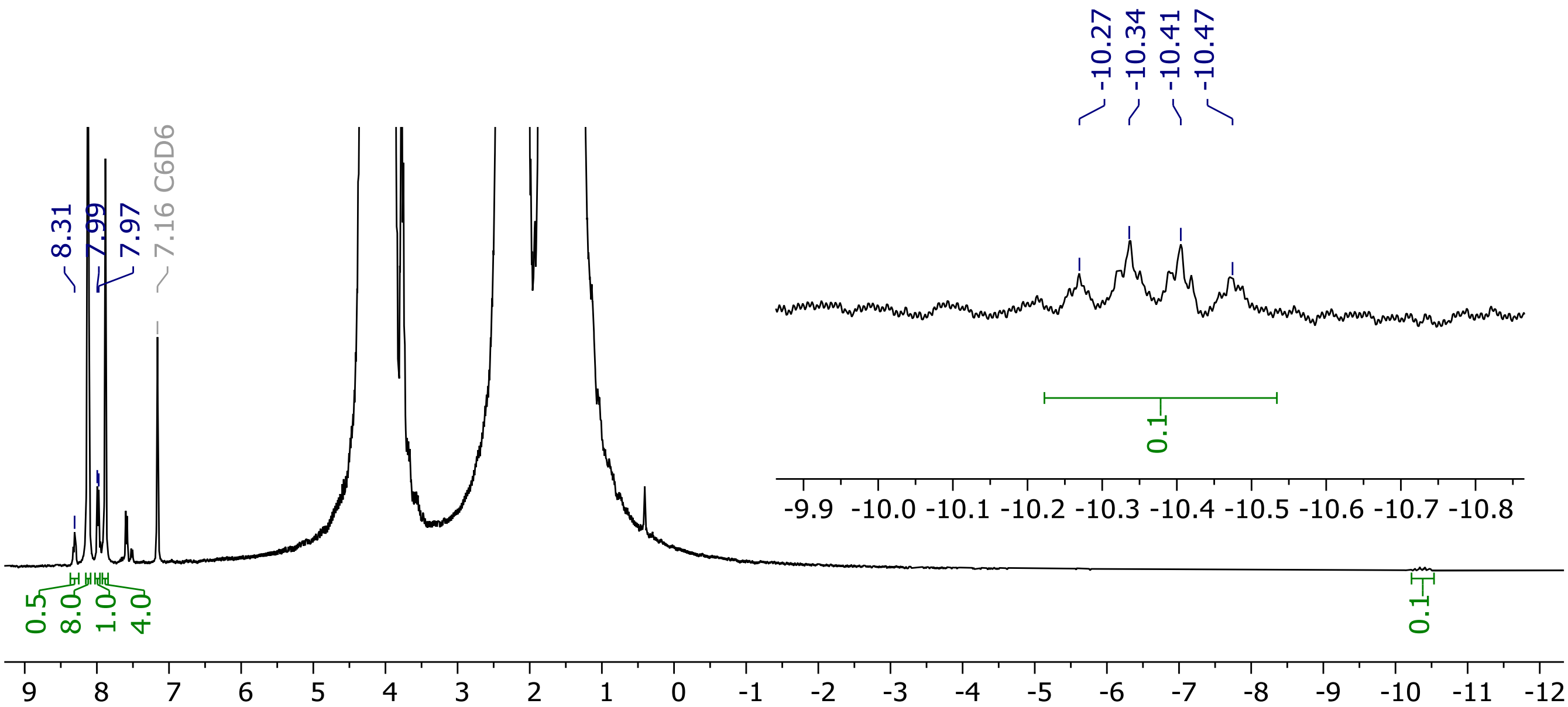

Figure S31. ^{1}H NMR spectrum of d_1-**1** (10 mM) recorded after 360 min in MeTHF at RT (400 MHz).

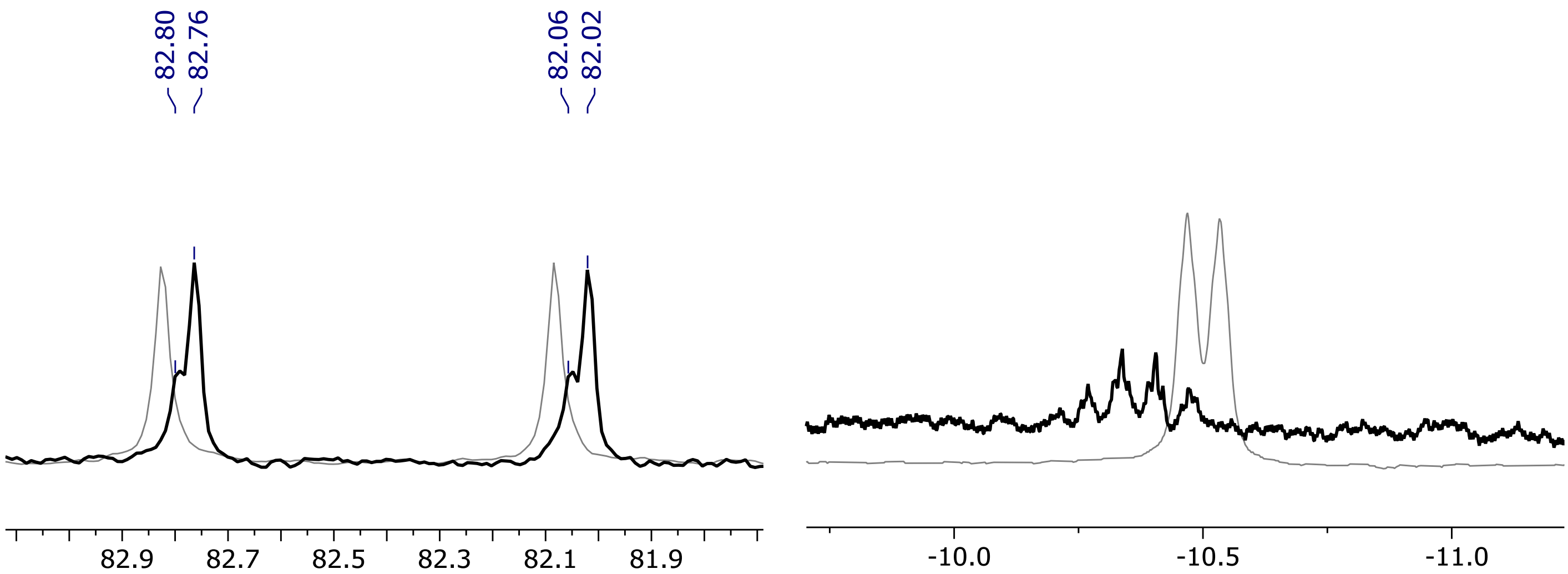

Figure S32. Comparison of the $^{31}P\{^1H\}$ NMR spectra (left) and ^1H NMR spectra (right) of the isotopologues of **3** formed from decomposition of d_0-**1** (grey) and d_1-**1** (black).

4.2 Characterisation by EPR spectroscopy

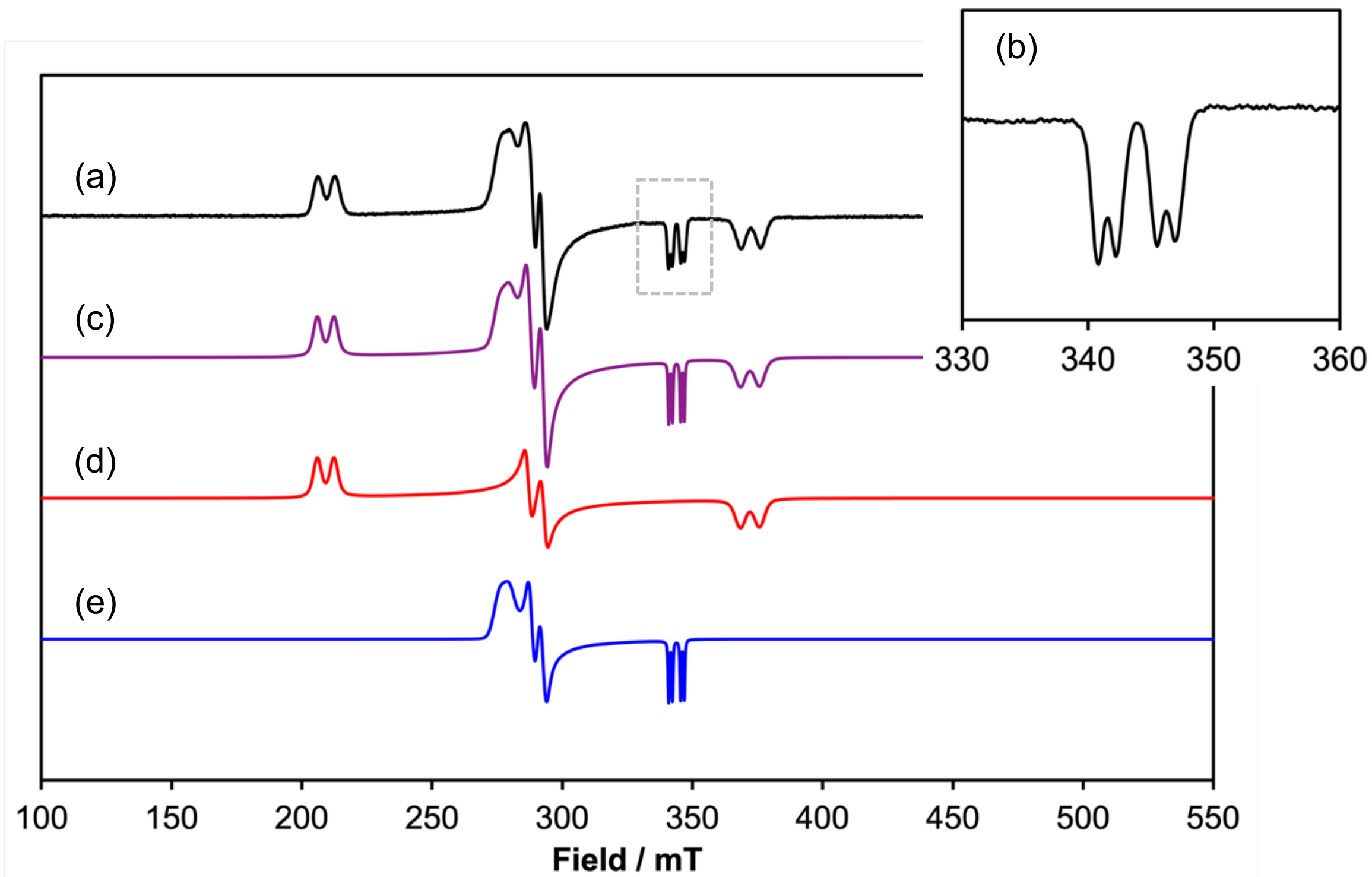

Figure S33. CW X-band EPR spectrum of **1** recorded in MeTHF frozen glass at 115 K. (a) experimental spectrum with dashed region magnified (b), (c) full simulated spectrum, and simulated spectra of **1** (d) and **1**·MeTHF (e). **1**:**1**·MeTHF ratio = 1.0:1.3.

4.3 Characterisation by SCXRD

The structure of **1** was refined full-matrix least squares on F^2. All non-hydrogen atoms were refined anisotropically. The hydrido ligand was located off the Fourier difference map (Figure S34) and refined isotopically with a restrained Rh–H bond length of 1.600 Å, based on the location of the Fourier peak and by comparison to preceding data collections. All other hydrogen atoms were placed in calculated positions

and refined using the riding model. There is no significant crystallographic disorder evident in the cation (Figure S35). Rotational disorder of the CF_3 groups of the anion was treated by modelling the fluorine atoms over two sites and restraining their geometry. Restraints to thermal parameters were applied to the fluorine atoms to maintain sensible values.

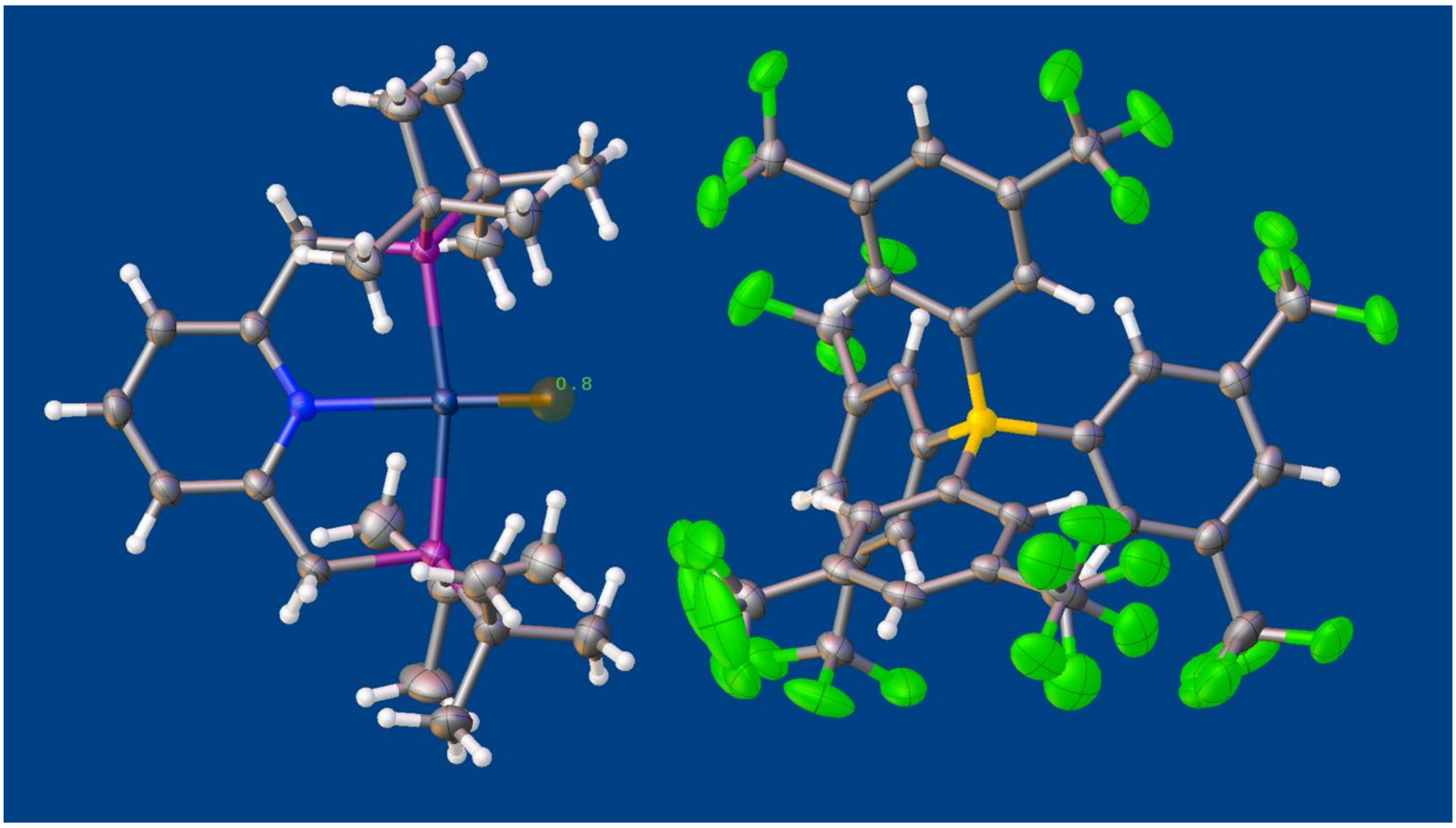

Figure S34. Location of the hydrido ligand from the difference map for the partially refined SCXRD structure of **1**. Density of Fourier peak in eÅ^{-3}.

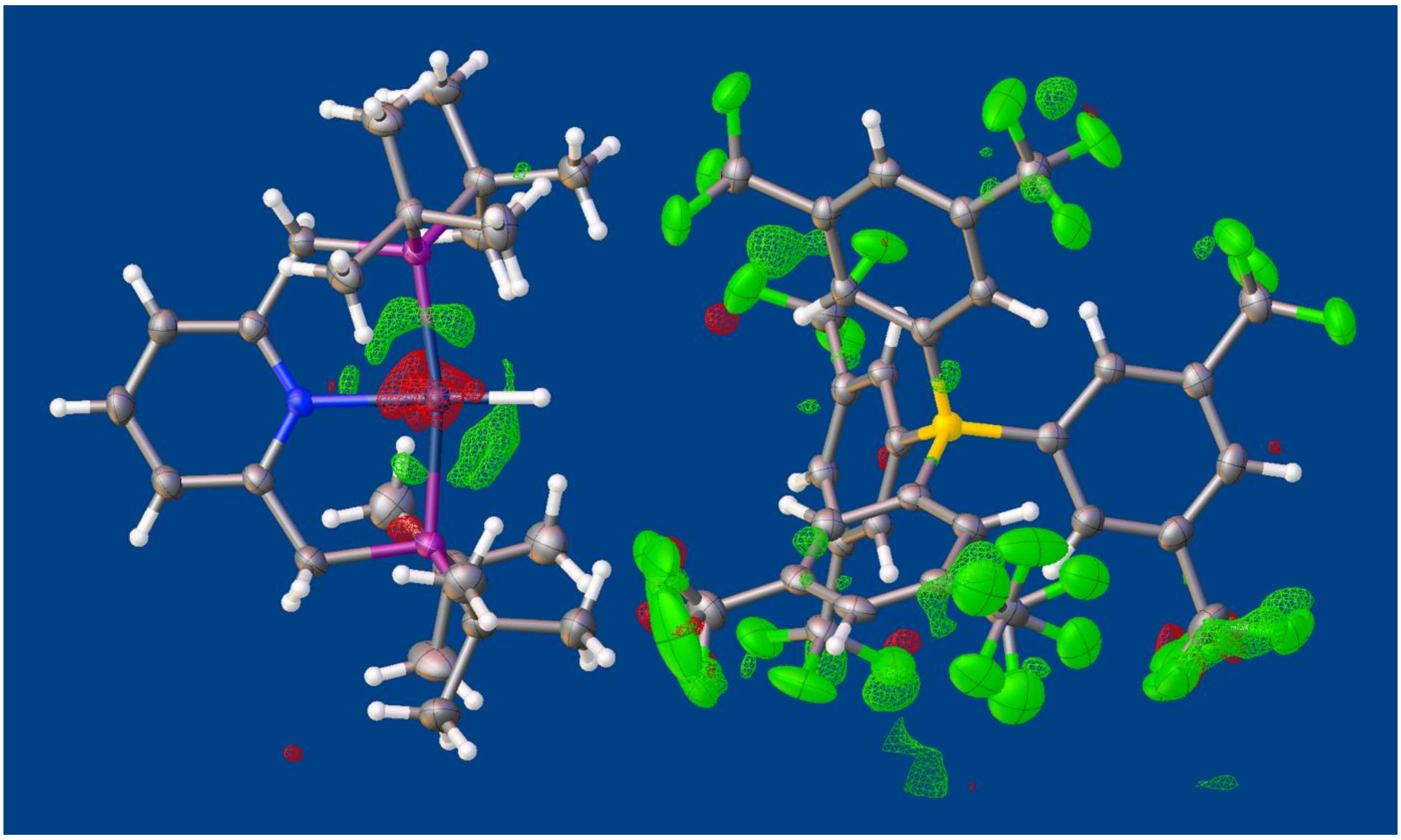

Figure S35. Difference map for the refined SCXRD structure of **1**, showing residual density > 0.3 eÅ^{-3}.

5 Preparation and characterisation of [Rh(PNP-*t*Bu)(OH)][BAr$^F_4$] 2

Method A: A J. Young valve NMR tube charged with microcrystalline **4** (6.9 mg, 5.01 µmol) was successively placed under H_2 (1 atm) for 1 h at RT, high vacuum, and N_2O (1 atm) for 1 h at RT. The headspace was analysed by GC-TCD, indicating the generation of N_2, and the light peach powder was characterised by ATR-IR and NMR spectroscopies.

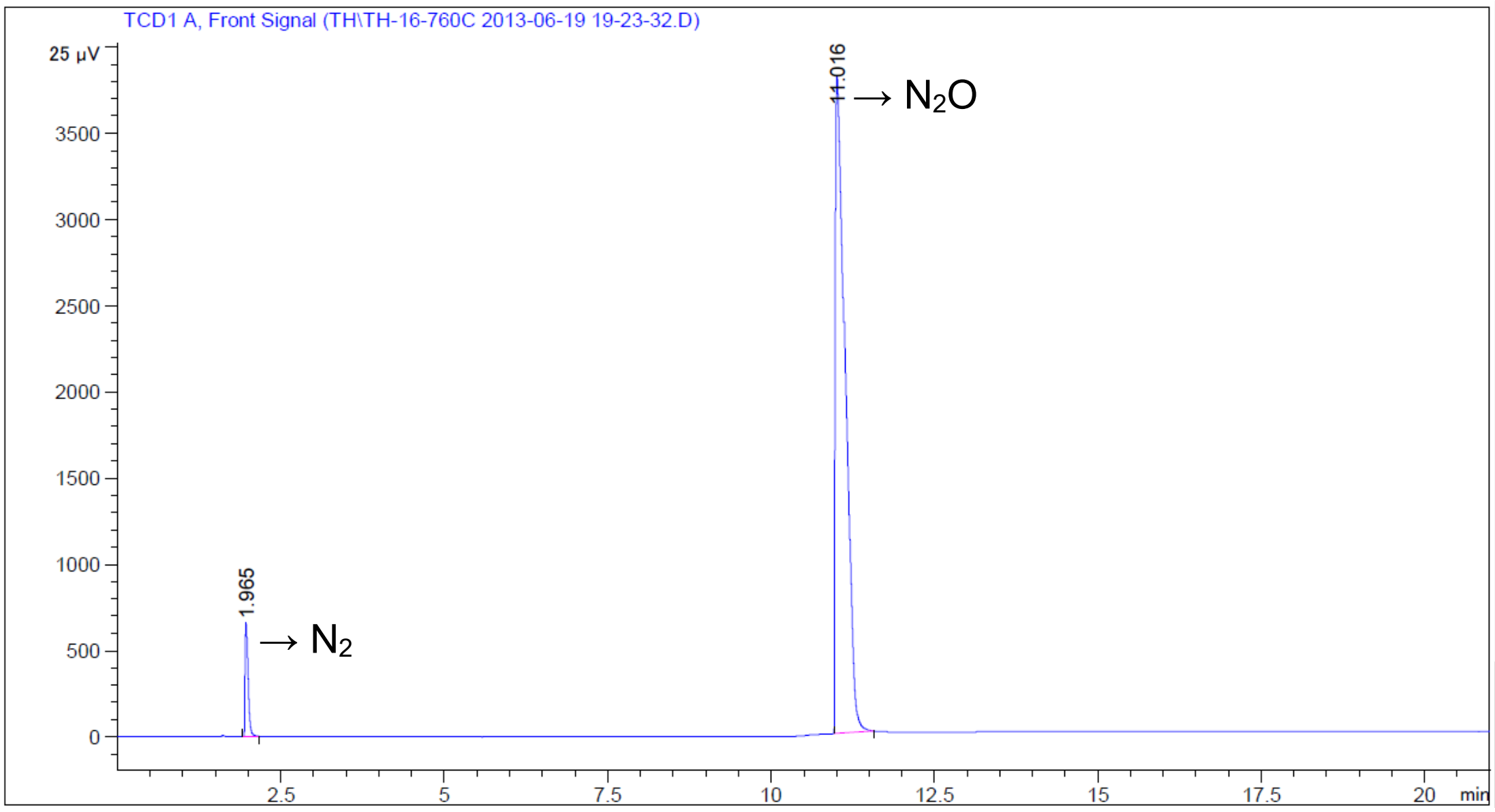

Figure S36. GC-TCD trace collected following the reaction of **1** with N_2O.

Method B: A J. Young valve EPR tube charged with microcrystalline **4** (3.4 mg, 2.47 µmol) was successively placed under H_2 (1 atm) for 1 h at RT, high vacuum, and then N_2O (1 atm) for 1 h at RT. The sample was placed under vacuum and MeTHF (250 µL) was added by vacuum transfer at 77 K. The product was dissolved in thawing MeTHF at 195 K to give a 10 mM solution which was thereafter frozen at 77 K and flame-sealed under vacuum. The sample was kept at 77 K until it was loaded into the pre-cooled spectrometer at 115 K.

Method C: Single crystals of **4** were successively placed under H_2 (1 atm) for 2 h at RT, high vacuum, and then N_2O (1 atm) for 2 h at RT. The resulting yellow crystals were analysed by SCXRD at 100 K.

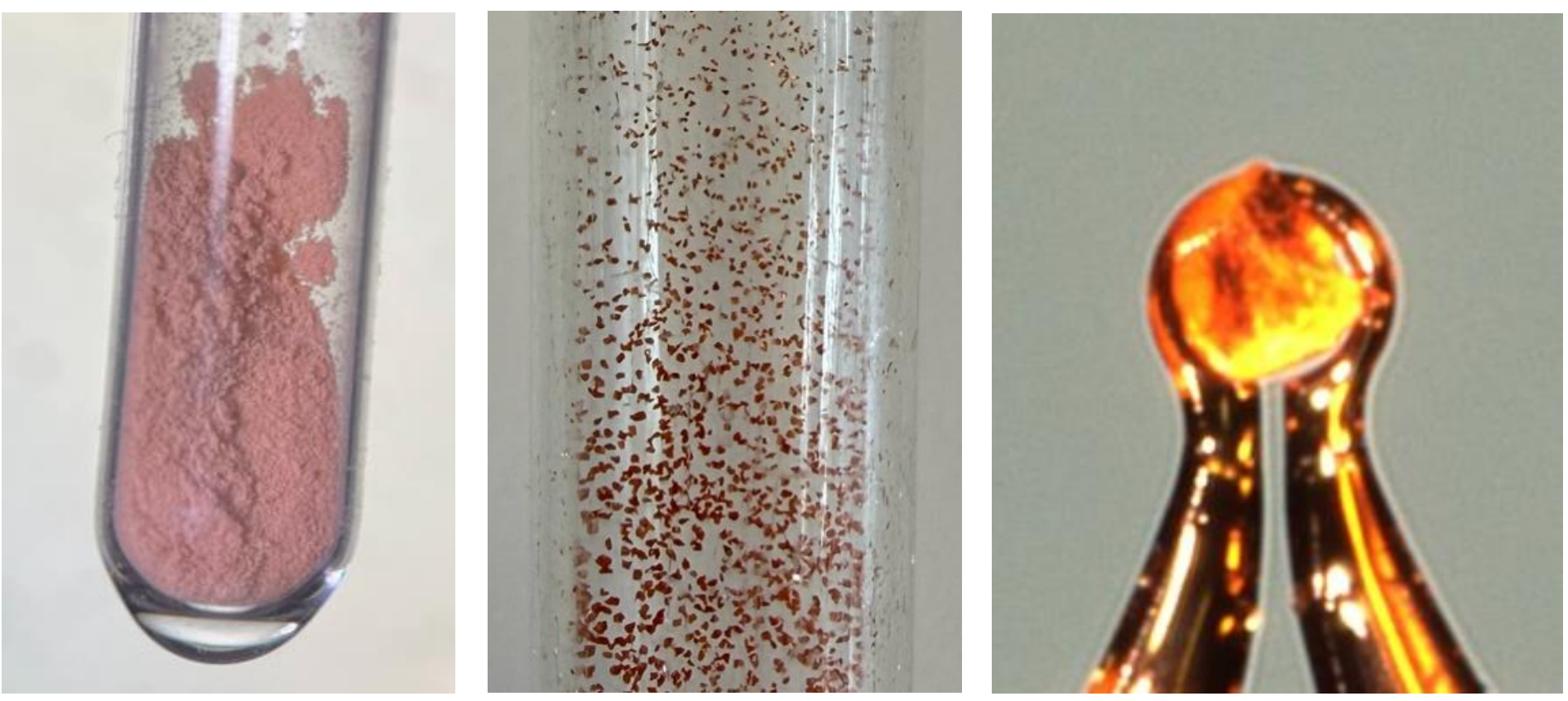

Figure S37. Samples of **2**: microcrystalline solid, single crystals, and single crystal.

^{1}H NMR (400 MHz, MeTHF, 273 K, selected data): δ 52.05 (vbr, fwhm = 660 Hz, 4H, CH_2), 19.8 (br, fwhm = 89, 2H, py) 8.02 (obscured vbr, fwhm = 520 Hz, 36H, *t*Bu), -64.65 (vbr, fwhm = 170 Hz, 1H, OH *or* py).
^{31}P{^{1}H} NMR (162 MHz, MeTHF, 193 K): no resonances observed between δ −600 and +600.
EPR (MeTHF glass, 115 K): **2**, **g** = [3.231 1.921 1.716], **A**(^{103}Rh) not resolved. **2**·MeTHF; **g** = [2.601 2.276 1.961], **A**(^{103}Rh) = [182 126 119] MHz. **2**:**2**·MeTHF ratio = 1.0:0.35.
IR (ATR): featureless hydride and hydroxide region. Spectrum on page S27.

5.1 Solution stability and characterisation by NMR spectroscopy

Isolated **2** was analysed in MeTHF (10 mM) by variable temperature ^{1}H and ^{31}P NMR spectroscopy. No diamagnetic impurities symptomatic of decomposition were observed when the sample was analysed at 193 K. Upon warming to RT, > 95% decomposition into a 1.0:2.0:4.3 mixture of an unknown diamagnetic rhodium complex (δ_{31P} 99.2, $^1J_{RhP}$ = 213 Hz), PNP-*t*Bu oxide (δ_{31P} 69.2),[2] and [Rh(PNP-*t*Bu)(OH_2)][BAr$^F{}_4$] (δ_{31P} 63.8, $^1J_{RhP}$ = 144 Hz, verified by independent synthesis)[7] was observed within 5 min.

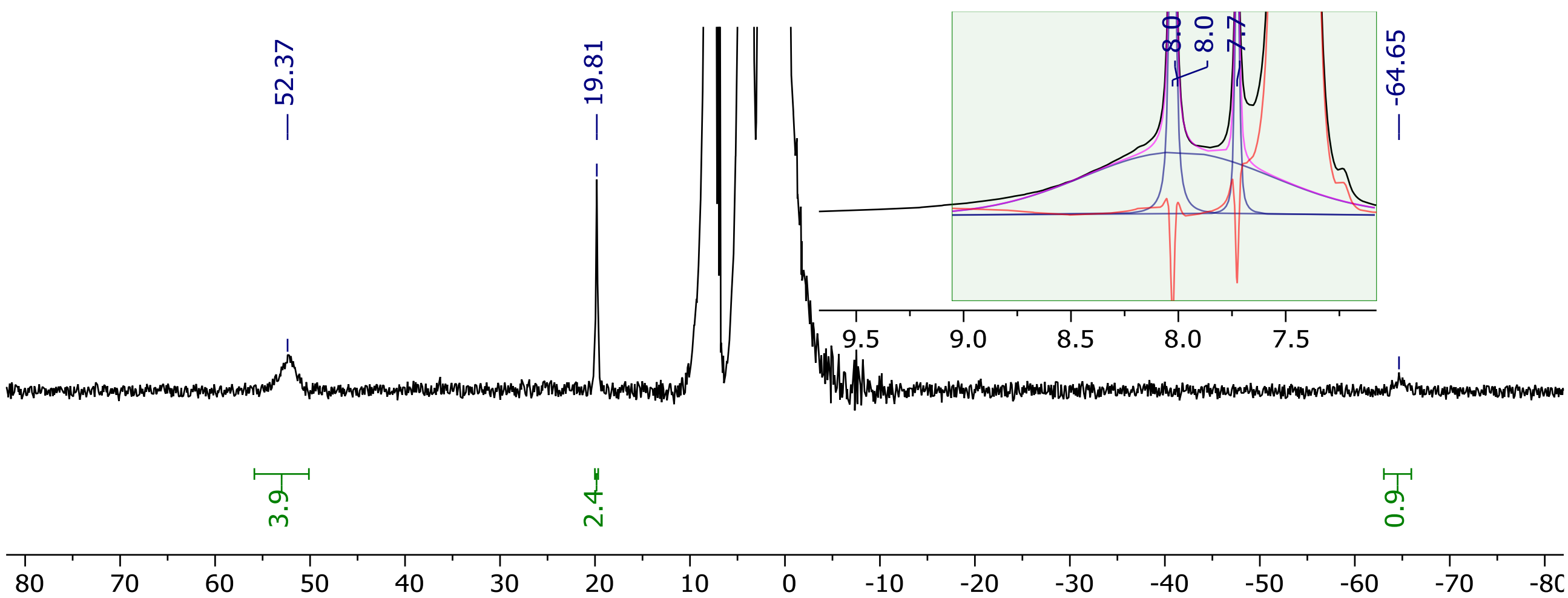

Figure S38. ^{1}H NMR spectrum of **2** (10 mM) in MeTHF at 273 K (500 MHz).

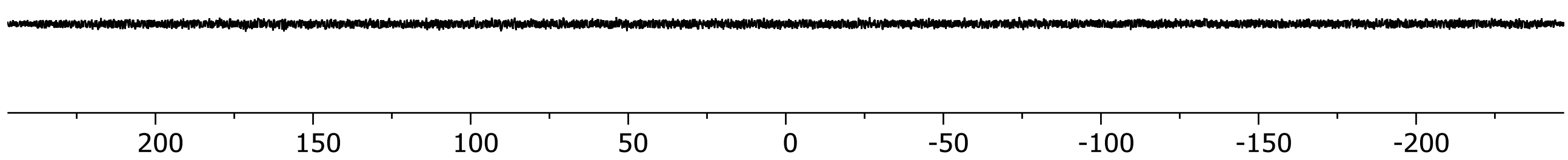

Figure S39. ^{31}P{^{1}H} NMR spectrum of **2** (10 mM) in MeTHF at 193 K (202 MHz).

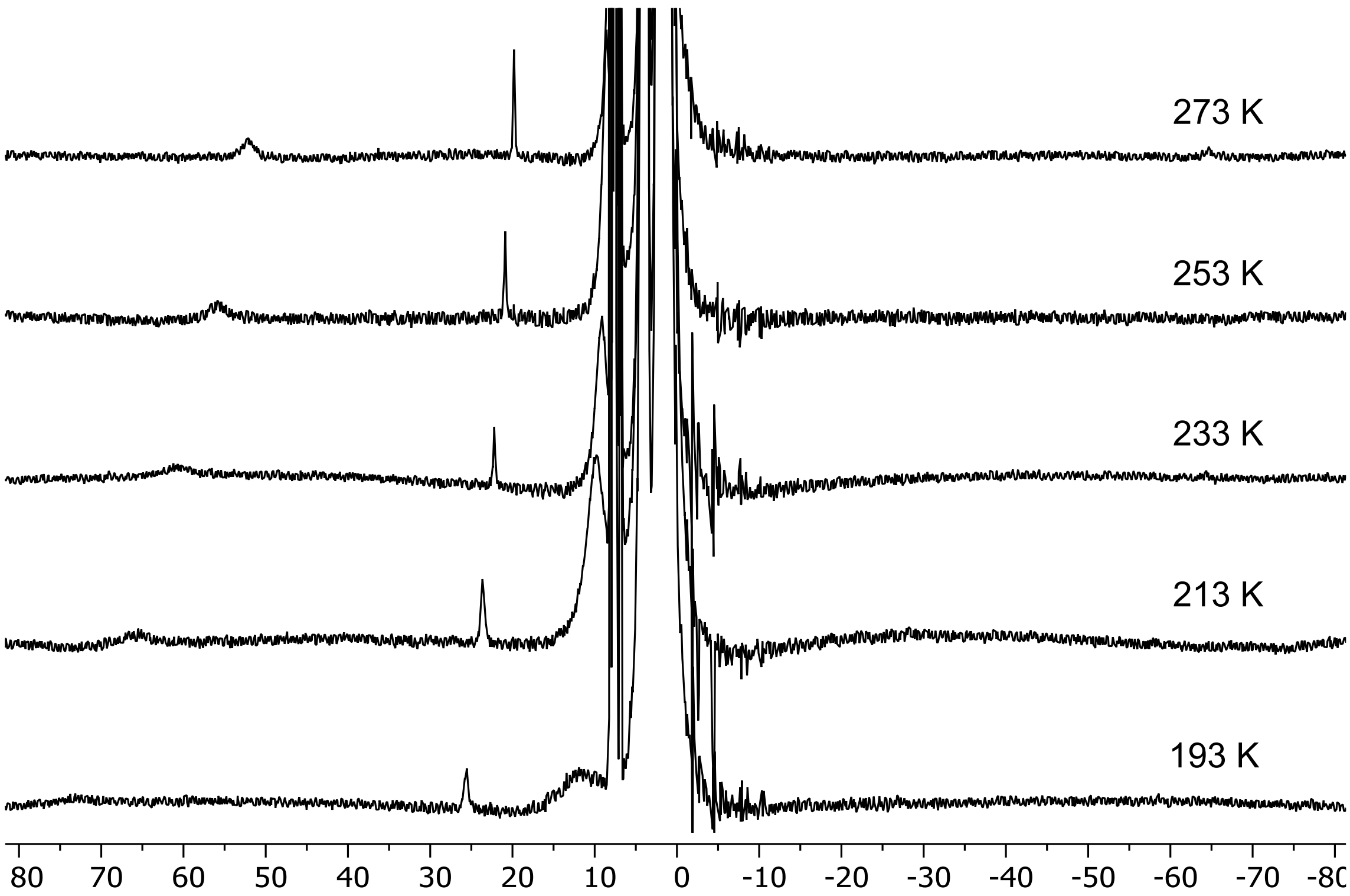

Figure S40. Variable temperature ^1H NMR spectra of **2** (10 mM) in MeTHF (500 MHz).

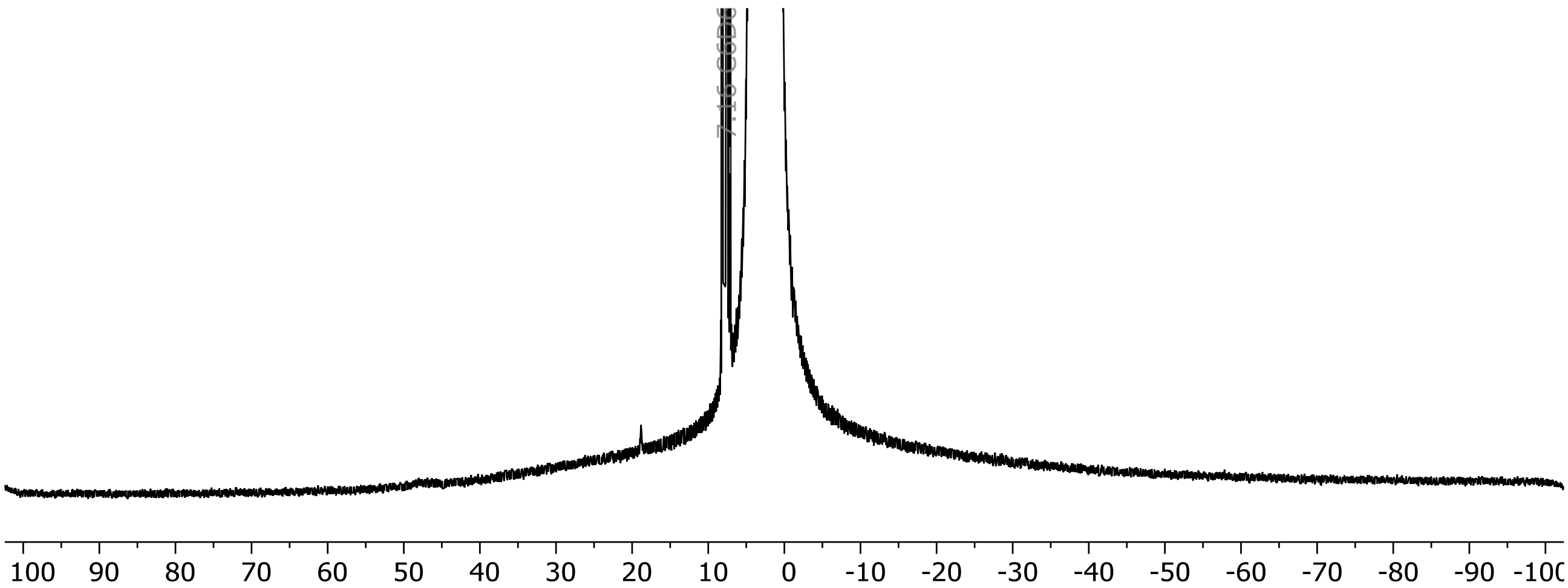

Figure S41. ^1H NMR spectrum of **2** (10 mM) recorded in MeTHF after < 5 min at RT (500 MHz). > 95% decomposition.

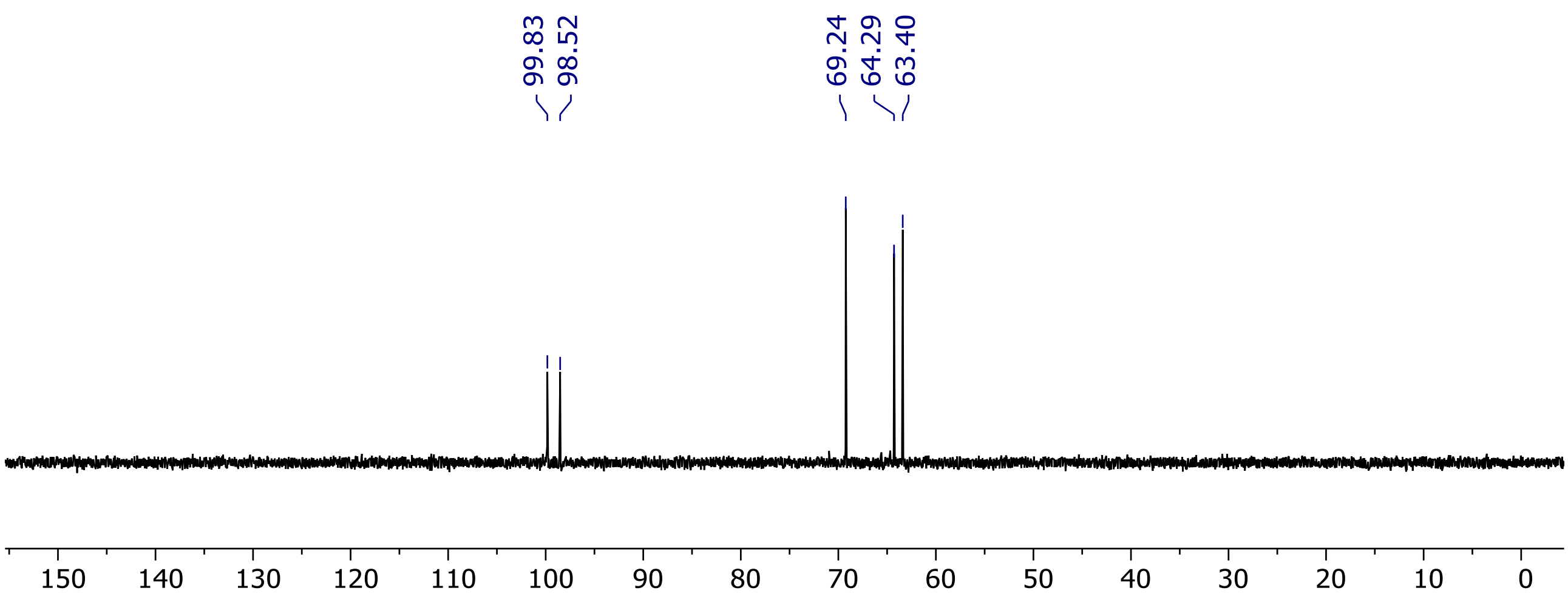

Figure S42. $^{31}P\{^1H\}$ NMR spectrum of **2** (10 mM) recorded in MeTHF after < 5 min at RT (162 MHz).

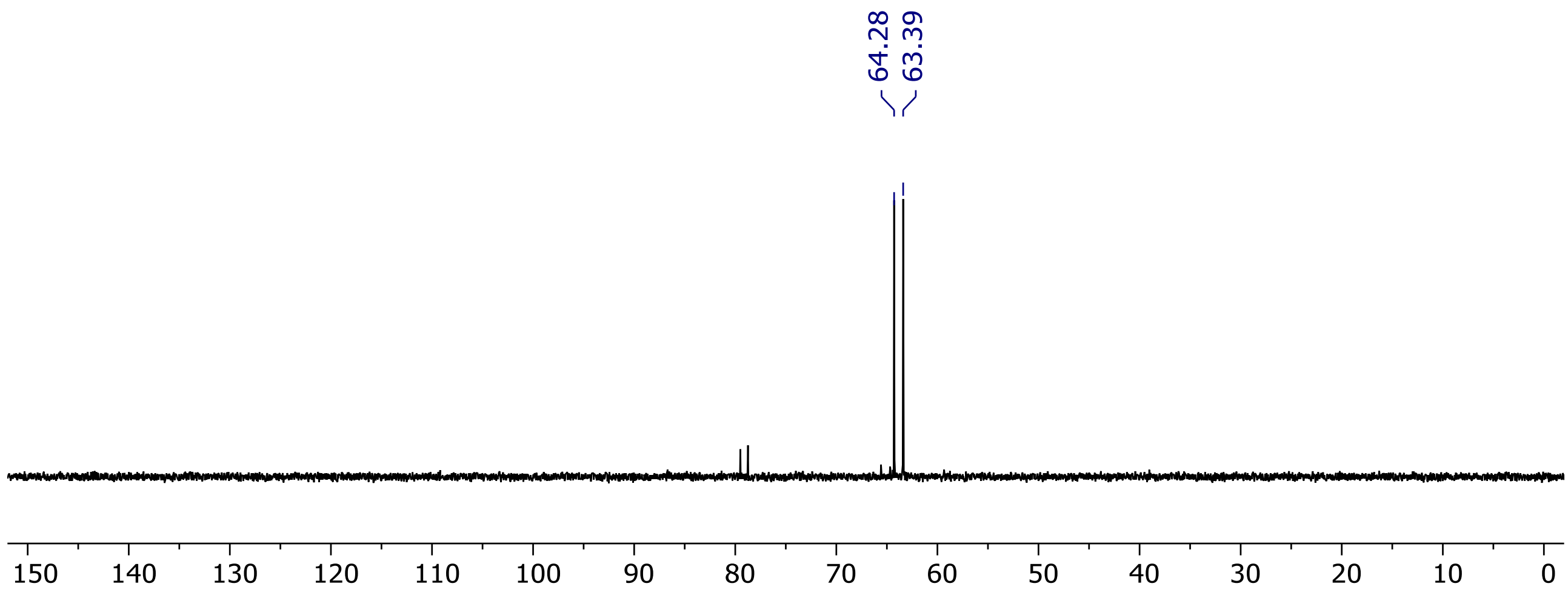

Figure S43. $^{31}P\{^{1}H\}$ NMR spectrum of independently prepared [Rh(PNP-*t*Bu)(H_2O)][BAr^{F}_4] (95% purity) in MeTHF (162 MHz).

5.2 Characterisation by EPR spectroscopy

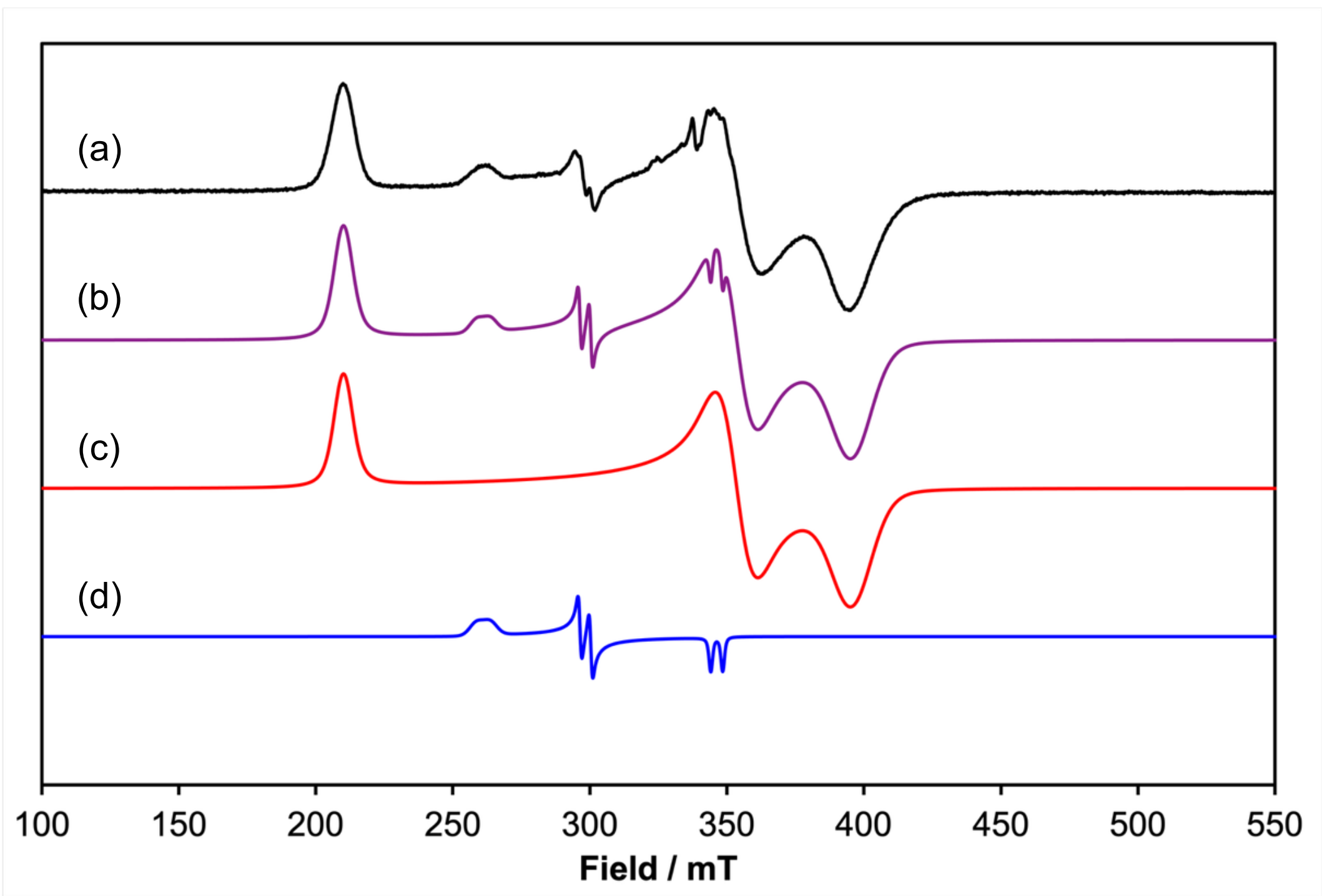

Figure S44. CW X-band EPR spectrum of **2** recorded in MeTHF frozen glass at 115 K. (a) experimental spectrum, (b) full simulated spectrum, and simulated spectra of **2** (c) and **2**·MeTHF (d). **2**:**2**·MeTHF ratio = 1.0:0.35

5.3 Characterisation by SCXRD

The structure of **2** was refined full-matrix least squares on F^2. All non-hydrogen atoms were refined anisotropically. Hydrogen atoms were placed in calculated positions and refined using the riding model, noting that the location of the hydroxo ligand proton was corroborated by inspection of the difference map (Figure S45). There is no significant crystallographic disorder evident in the cation (Figure S46). Rotational disorder of the CF_3 groups of the anion was treated by modelling the fluorine atoms over two sites and

restraining their geometry. Restraints to thermal parameters were applied to the fluorine atoms to maintain sensible values.

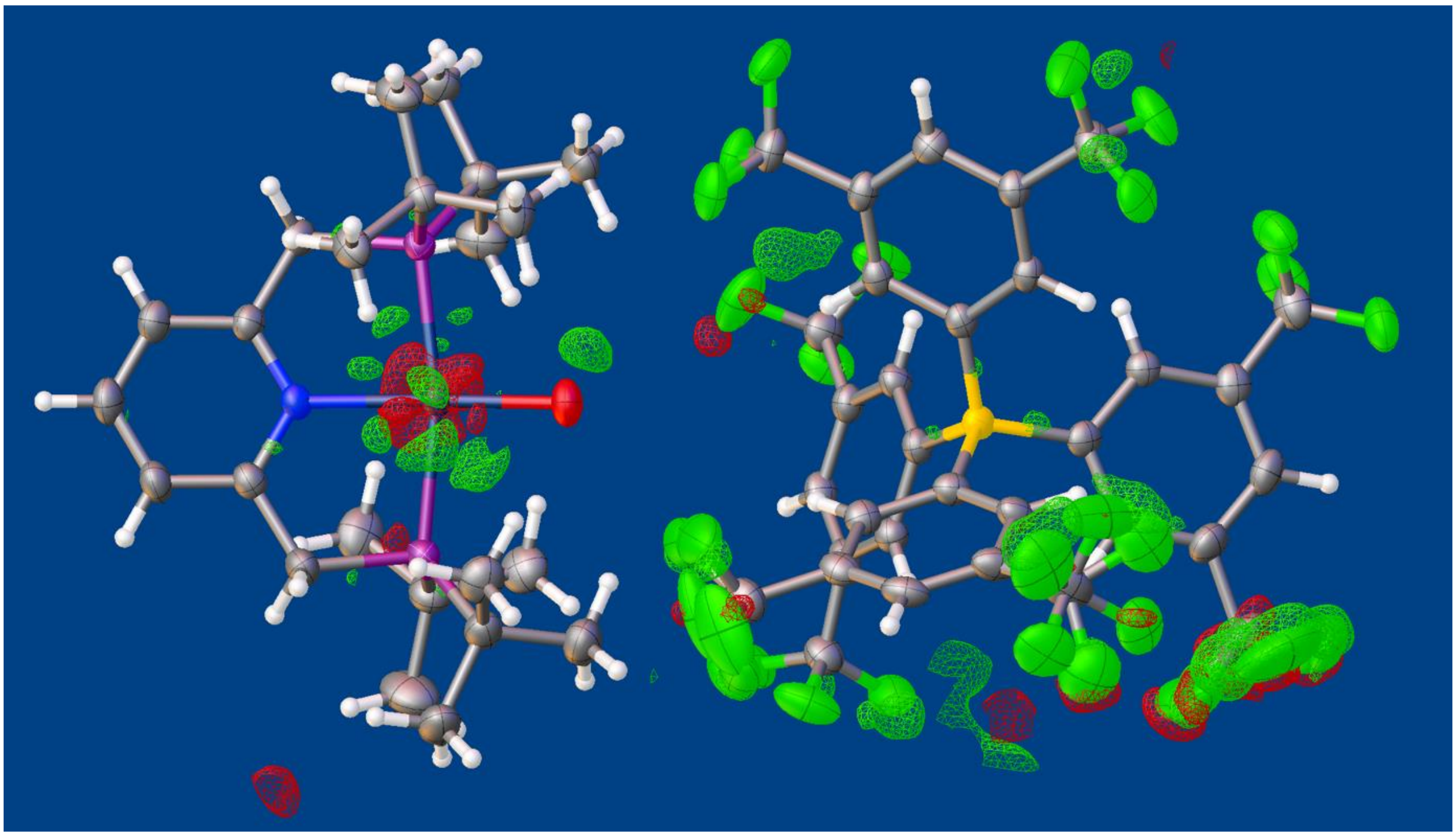

Figure S45. Difference map for the partially refined SCXRD structure of **2**, showing the location of the hydroxo ligand proton and residual density > 0.3 eÅ^{-3}.

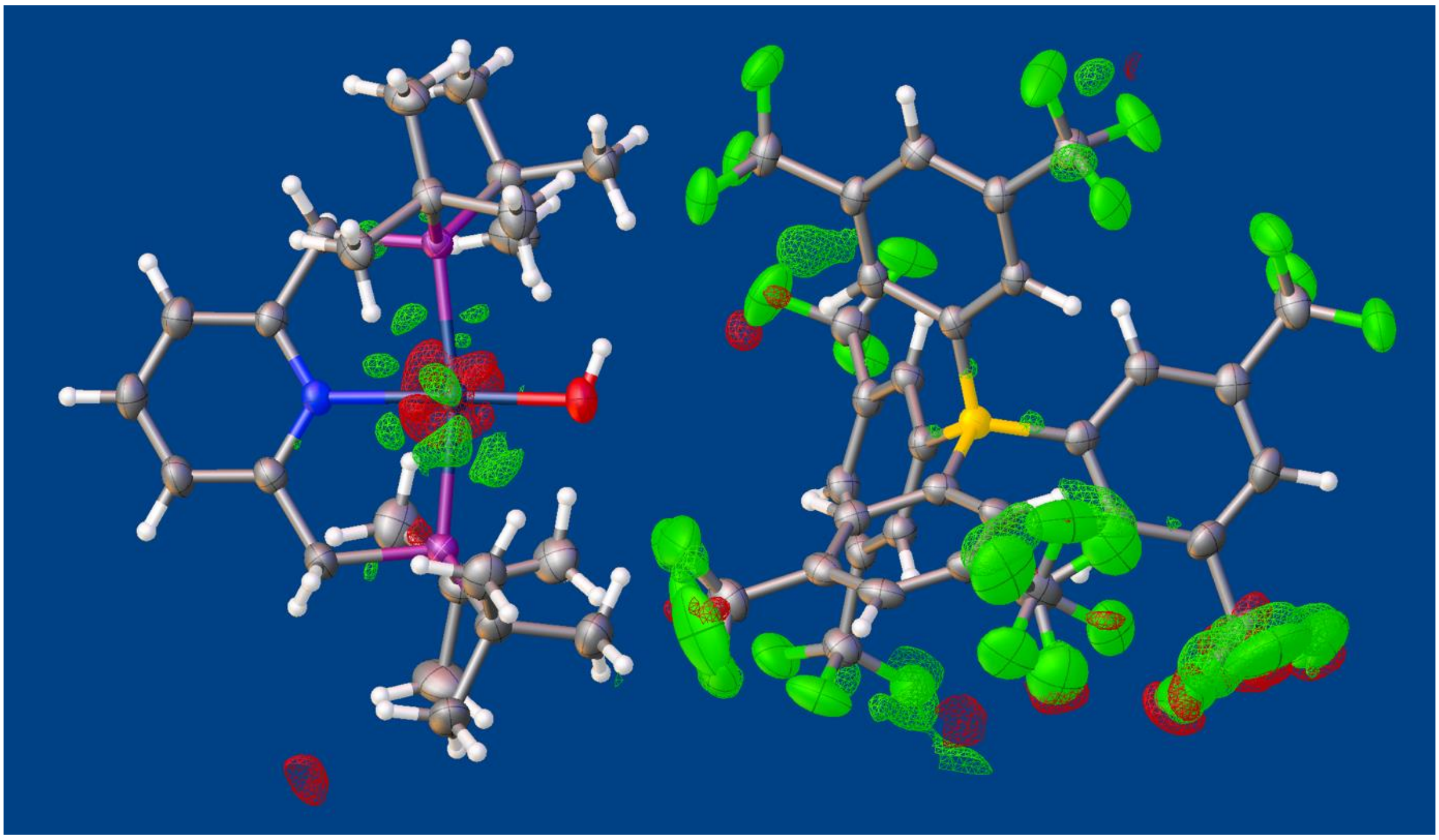

Figure S46. Difference map for the refined SCXRD structure of **2**, showing residual density > 0.3 eÅ^{-3}.

6 Solid-state reaction of [Rh(PNP-*t*Bu)(OH)][BAr$^F{}_4$] 2 with H_2/D_2.

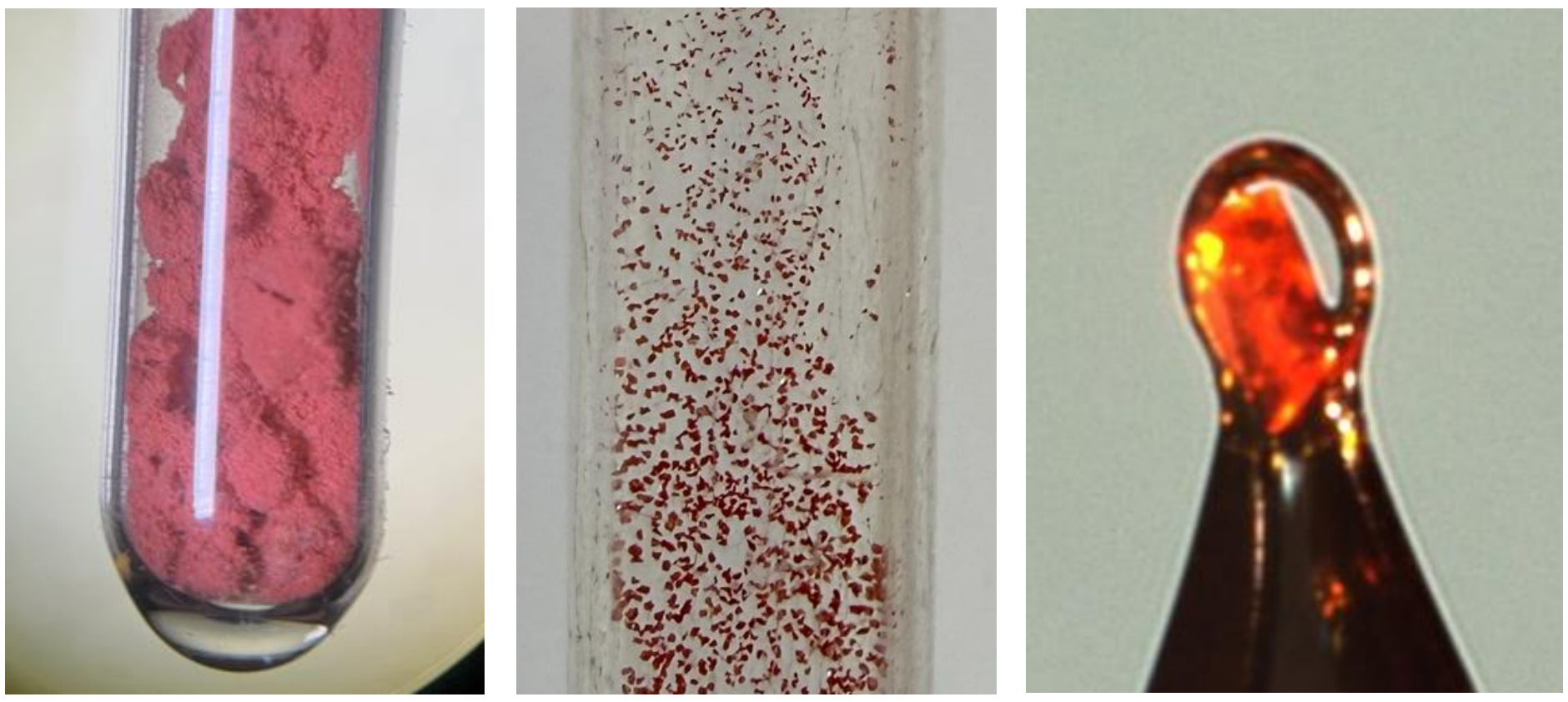

Figure S47. Samples of **2** + H_2: microcrystalline solid (after 5 h), single crystals, and single crystal (after 20 h).

6.1 Monitored by ATR-IR spectroscopy and GC-TCD

A J. Young valve NMR tube charged with microcrystalline **4** (6.9 mg, 5.01 µmol) was successively placed under H_2 (1 atm) for 1 h at RT, high vacuum, N_2O (1 atm) for 1 h at RT, high vacuum, and H_2 (1 atm) for 5 h at RT. The headspace was analysed by GC-TCD and the pink powder was characterised by ATR-IR spectroscopy, indicating the appearance of a *v*(Rh–H) band at 1941 cm^{-1}.

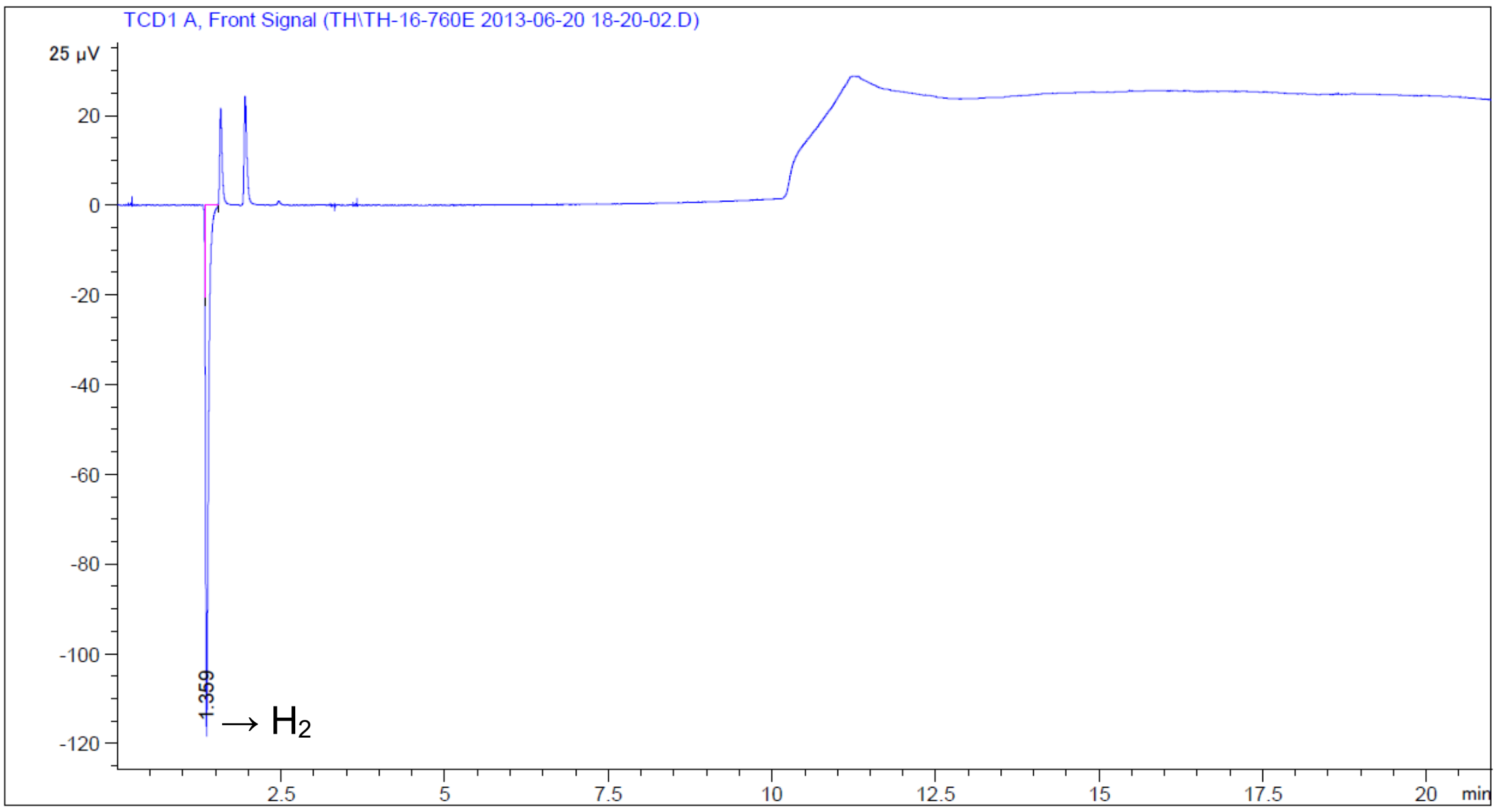

Figure S48. GC-TCD trace collected following the reaction of **2** + H_2 (5 h). Ar (1.6 min) and N_2 (2.0 min) are present in trace quantities (~ 20 µV) from the anaerobic GC-TCD setup.

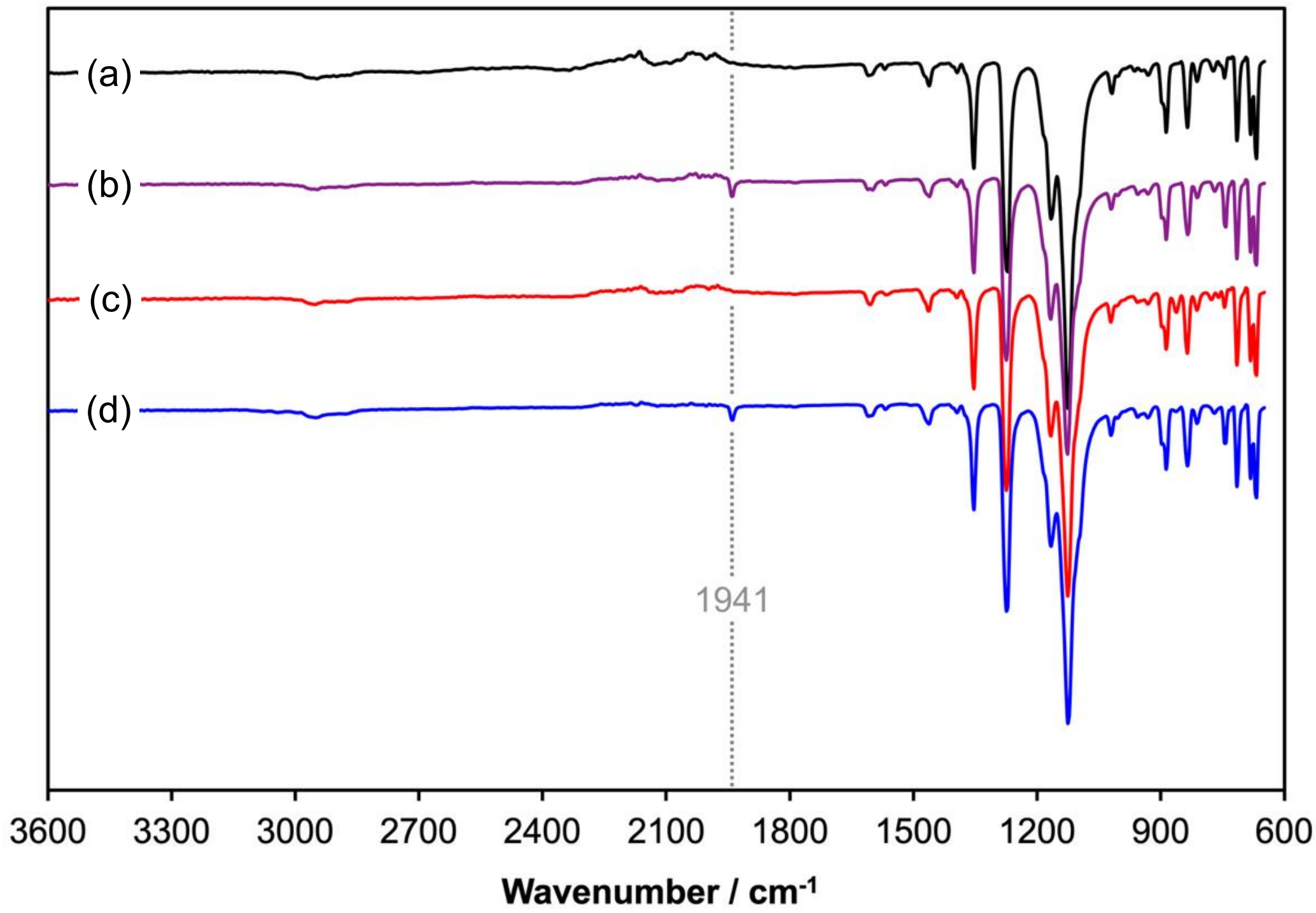

Figure S49. ATR-IR spectra of (a) **4**, (b) **1**, (c) **2**, (d) **2** + H_2 (5 h), with dashed line highlighting the position of the *v*(Rh–H) band in **1**.

6.2 Monitored by NMR spectroscopy

A J. Young valve NMR tube charged with microcrystalline **4** (6.9 mg, 5.01 µmol) was successively placed under H_2 (1 atm) for 1 h at RT, high vacuum, N_2O (1 atm) for 1 h at RT, high vacuum, and H_2 (1 atm) for 5 h at RT. The tube was placed under high vacuum at 77 K to remove H_2 and then sealed under static vacuum. The sample was warmed to RT and then successively placed under CO (1 atm) for 5 min, high vacuum at 77 K, and argon (1 atm) at RT. THF (0.5 mL) was added and the resulting 10 mM solution analysed by ^{1}H and ^{31}P NMR spectroscopy, indicating the formation of [Rh(PNP-*t*Bu)(CO)][BAr$^F_4$] **6** (δ_{31P} 79.3, $^1J_{RhP}$ = 120 Hz, >99%)[7] and 1 equivalent of H_2O (δ_{1H} 2.78).[2] When the reaction sequence was repeated, but using D_2 in place of H_2 in the third step, no D-atom incorporation into the pincer ligand was observed by ^{1}H NMR spectroscopy in THF and upon redissolution of **6** in CD_2Cl_2 (0.5 mL).

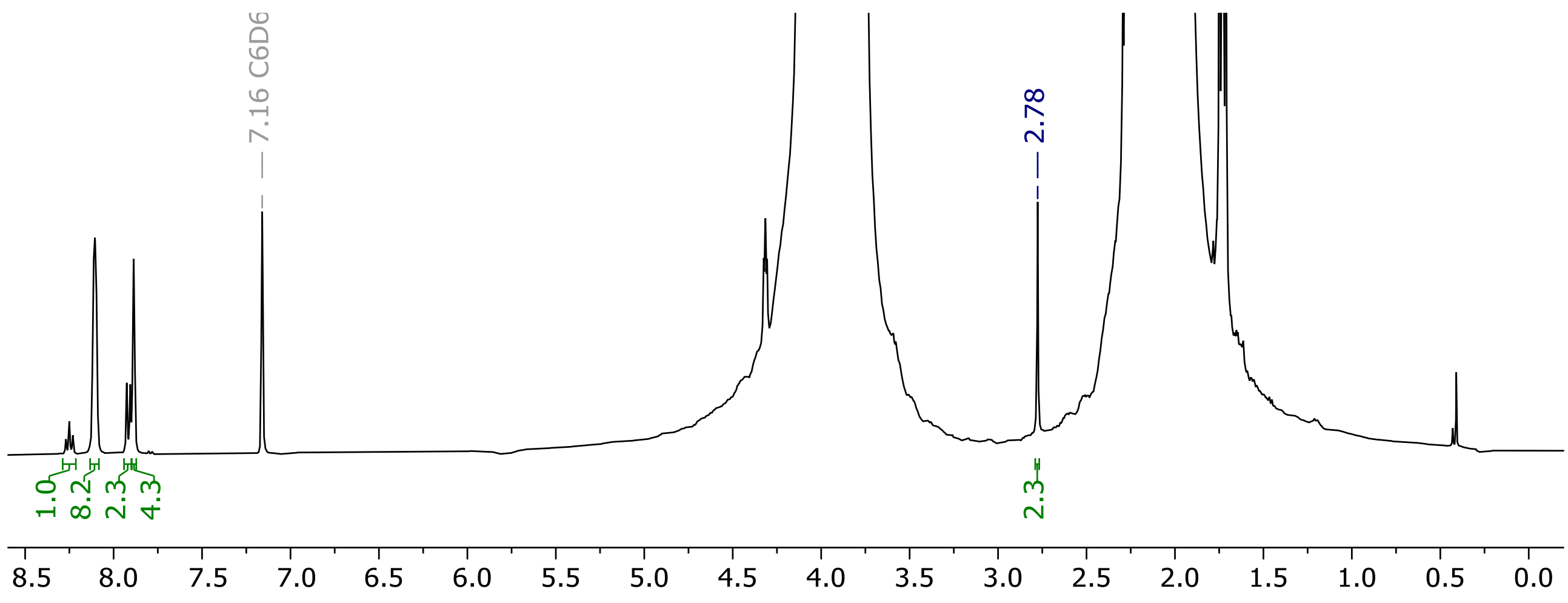

Figure S50. ^{1}H NMR spectrum of **2** + H_2 (5 h) recorded in THF (400 MHz, d1 = 30 s).

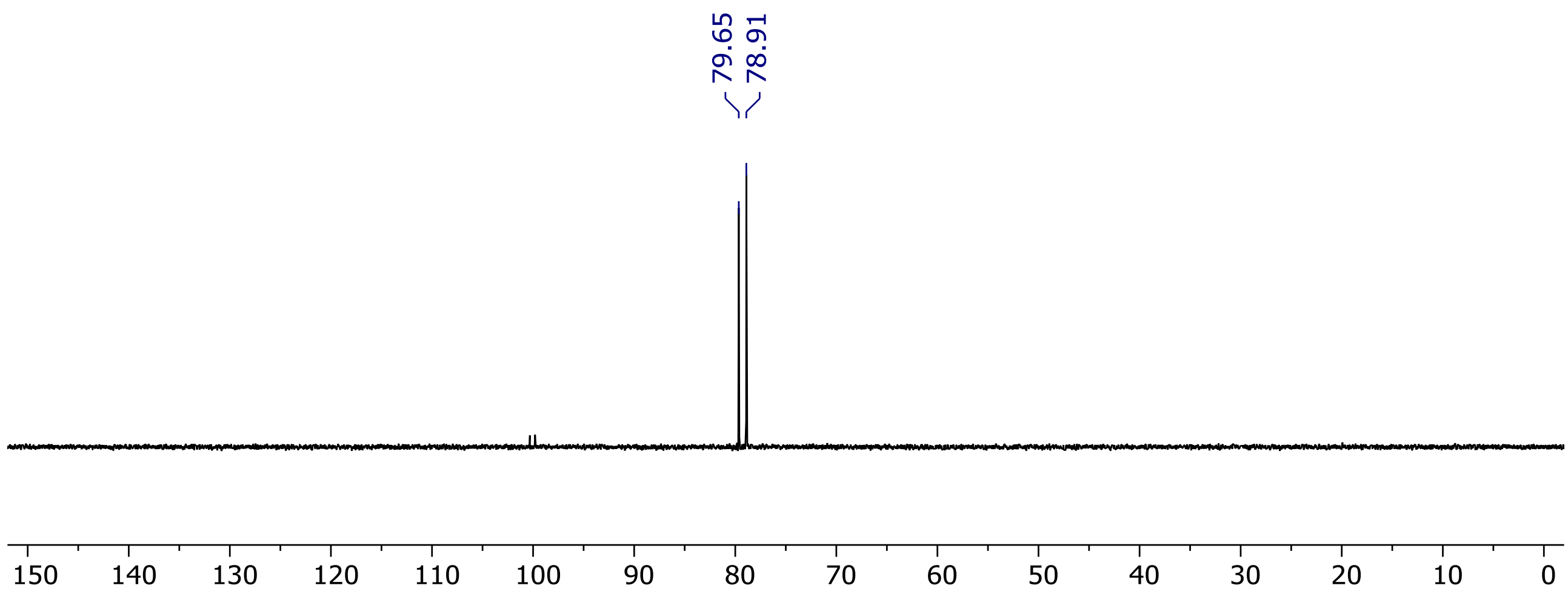

Figure S51. $^{31}P\{^{1}H\}$ NMR spectrum of **2** + H_2 (5 h) recorded in THF (162 MHz). > 99% complex **6**.

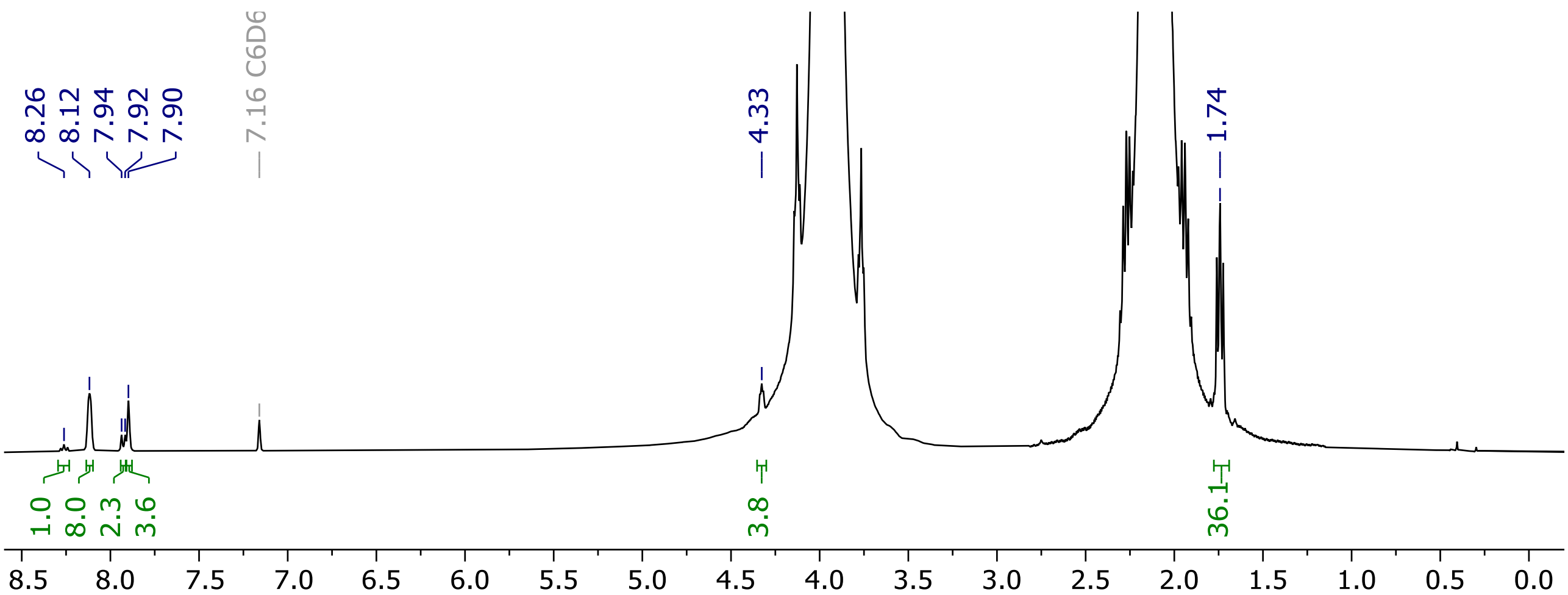

Figure S52. ^{1}H NMR spectrum of **2** + D_2 (5 h) recorded in THF (400 MHz).

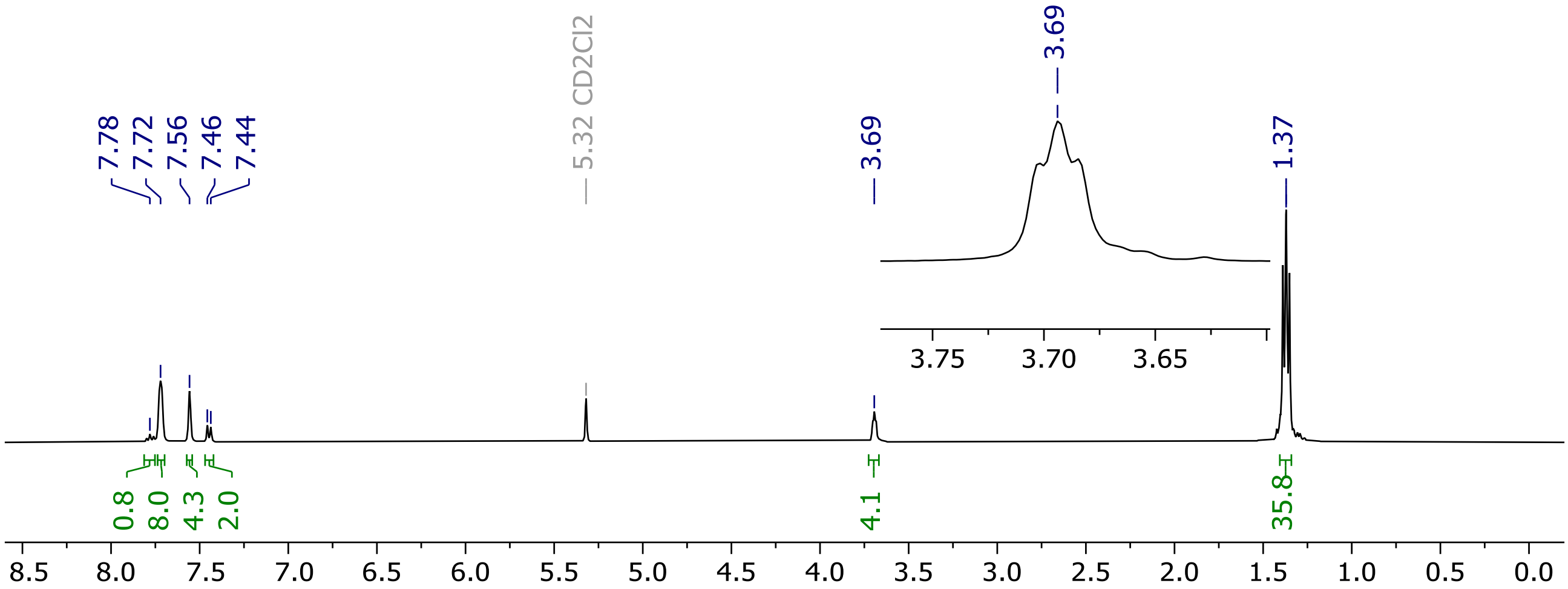

Figure S53. ^{1}H NMR spectrum of **2** + D_2 (5 h) recorded in CD_2Cl_2 (400 MHz)..

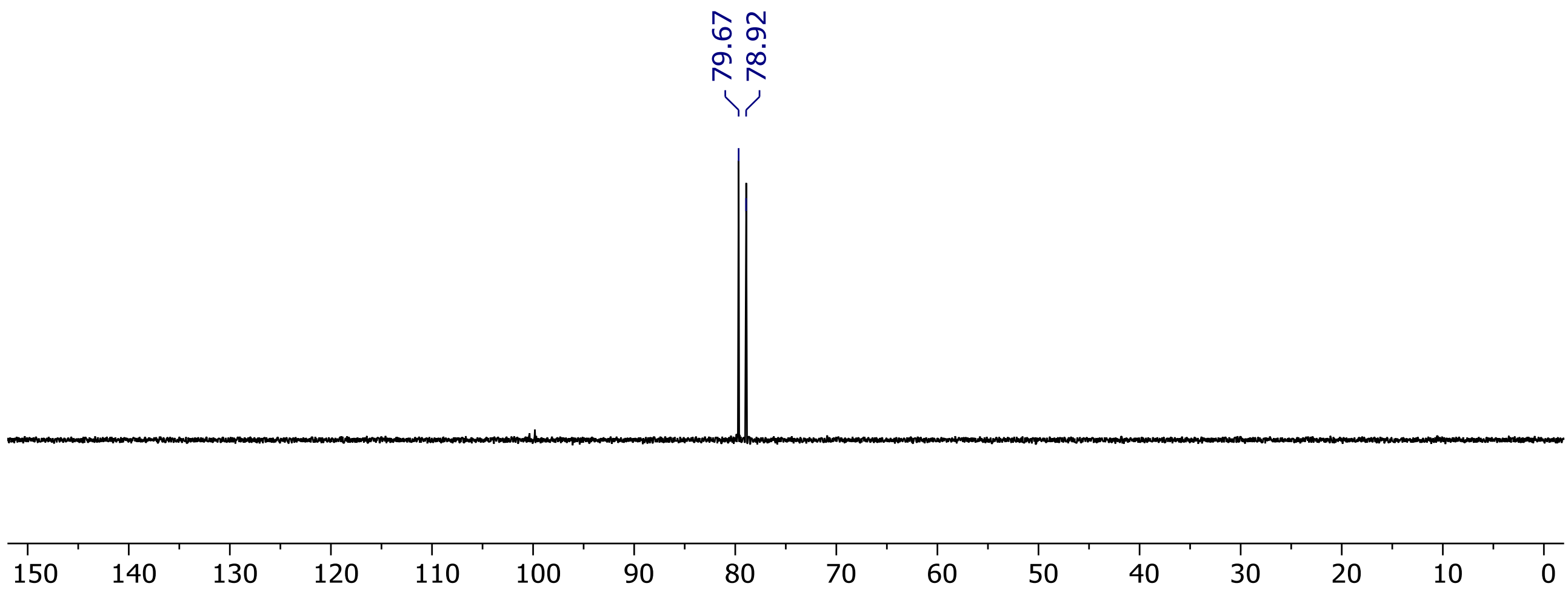

Figure S54. $^{31}P\{^1H\}$ NMR spectrum of **2** + D_2 (5 h) recorded in THF (162 MHz). > 99% complex **6**.

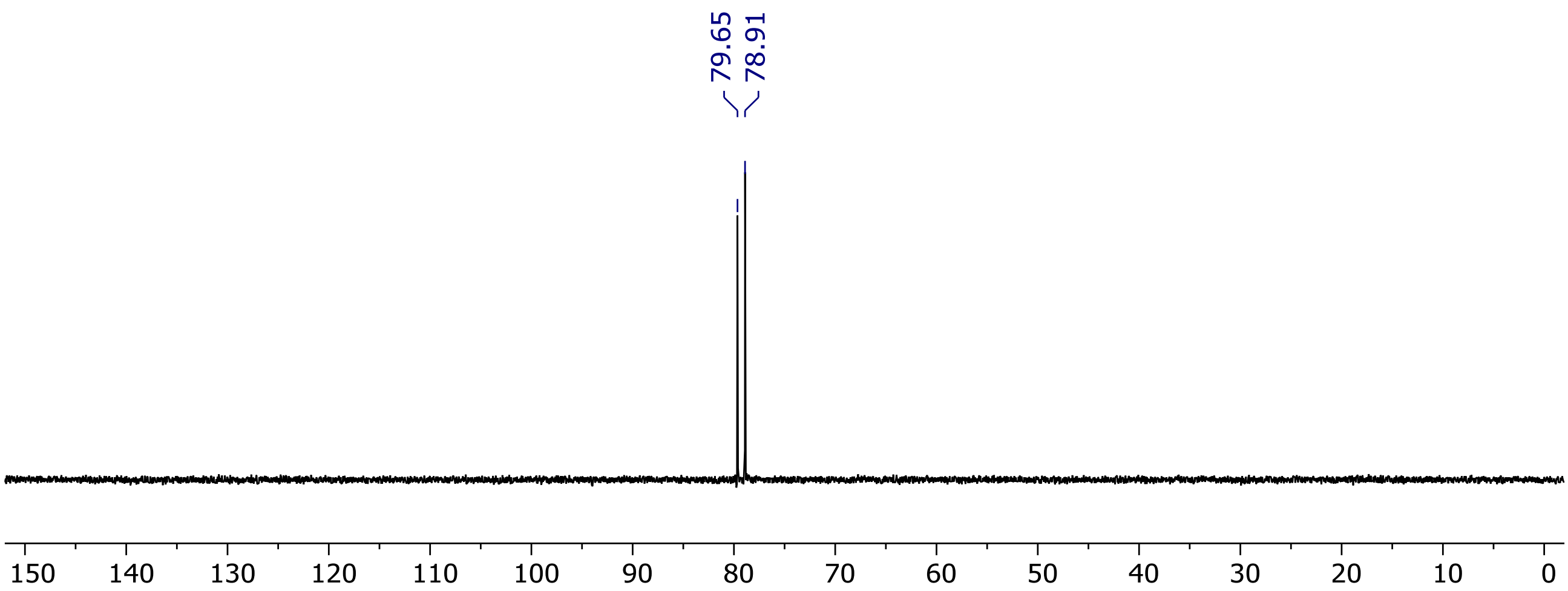

Figure S55. $^{31}P\{^1H\}$ NMR spectrum of independently prepared **6** in THF (162 MHz).

6.3 Monitored by EPR spectroscopy

A J. Young valve EPR tube charged with microcrystalline **4** (3.4 mg, 2.47 μmol) was successively placed under H_2 (1 atm) for 1 h at RT, high vacuum, and N_2O (1 atm) for 1 h at RT, and H_2 (1 atm) for either 1 h or 5 h at RT. The two samples were placed under vacuum and MeTHF (250 μL) was added by vacuum transfer at 77 K. The products were dissolved in thawing MeTHF at 195 K to give 10 mM solutions which were thereafter frozen at 77 K and flame-sealed under vacuum. The samples were kept at 77 K until they were loaded into the pre-cooled spectrometer at 115 K. Modelling of the data indicated 71% conversion from **2** to **1** after 1 h and complete conversion from **2** to **1** with 5 h. The reduced intensity of **1** in these EPR spectra is attributed to the presence of water.

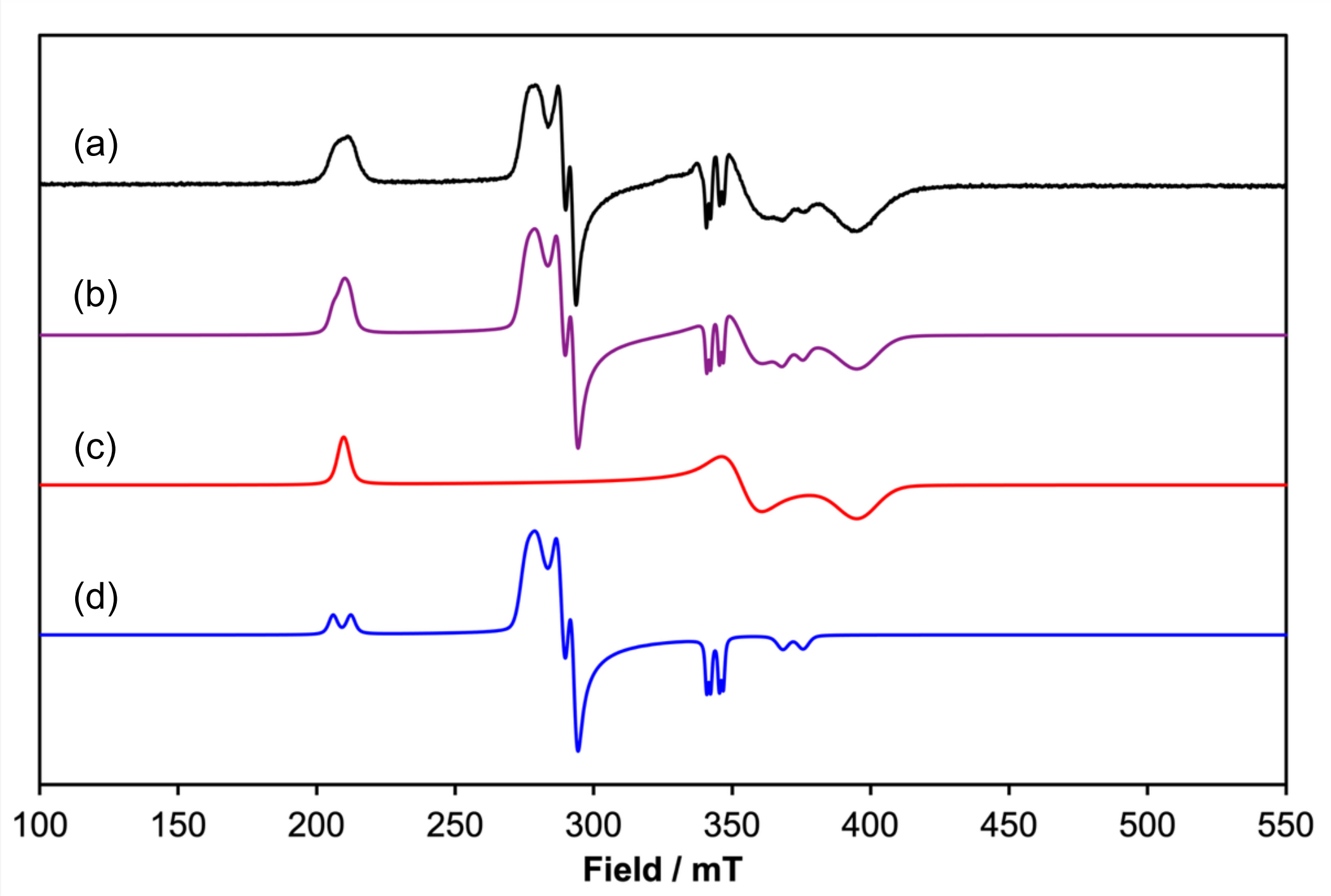

Figure S56. CW X-band EPR spectrum of **2** + H_2 after 1 h recorded in MeTHF frozen glass at 115 K. (a) experimental spectrum, (b) full simulated spectrum, and simulated spectra of **2** (c) and **1** + **1**·Solv (d). Conversion = 71.4%.

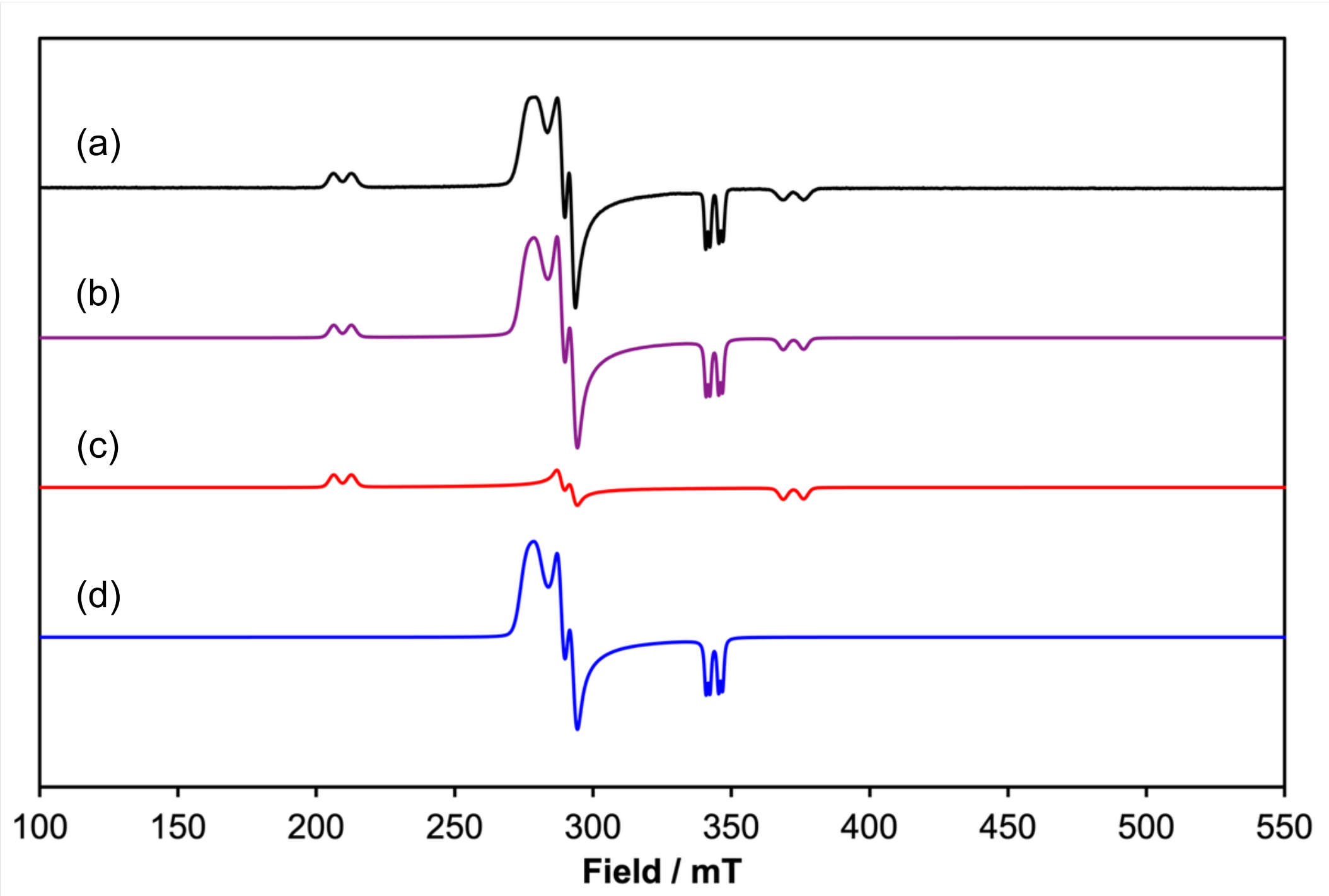

Figure S57. CW X-band EPR spectrum of **2** + H_2 after 5 h recorded in MeTHF frozen glass at 115 K. (a) experimental spectrum, (b) full simulated spectrum, and simulated spectra of **1** (c) and **1**·Solv (d). Conversion = 100%.

6.4 Monitored by SCXRD

Single crystals of **4** were successively placed under H_2 (1 atm) for 2 h at RT, high vacuum, and N_2O (1 atm) for 2 h at RT, and H_2 (1 atm) for either 10 h, 15 h or 20 h at RT. The resulting orange crystals were analysed by SCXRD at 100 K. The structures were refined full-matrix least squares on F^2. All non-hydrogen atoms were refined anisotropically. Increasing conversion from **2** into **1** over time is evident from the magnitude of the Fourier peak near rhodium (2.5, 1.4 and 1.1 e$Å^{-3}$; Figures S58, S60 and S62) and was quantified by disorder modelling (78.2(9), 91.7(5) and 94.8(5)%). The hydrido ligand was refined as the major disordered component, with a restrained Rh–H bond length of 1.600 Å based on the SCXRD structure of **1**. The hydroxo ligand was refined as the minor disordered component, with a restrained Rh–O bond length of 1.927 Å based on the SCXRD structure of **2**. A planarity restraint was applied across the metal centre in each case to maintain sensible geometries of the hydrido and hydroxo ligands but results in a non-negligible build-up of residual electron density around the nitrogen atom of the pyridine ring (Figures S59, S61 and S63). All other hydrogen atoms were placed in calculated positions and refined using the riding model. Rotational disorder of the CF_3 groups of the anion was treated by modelling the fluorine atoms over two sites and restraining their geometry. To maintain sensible values, the thermal parameters of the pyridine nitrogen/hydroxo oxygen atoms were constrained (15 h and 20 h) and the thermal parameters of the fluorine atoms restrained (all time points).

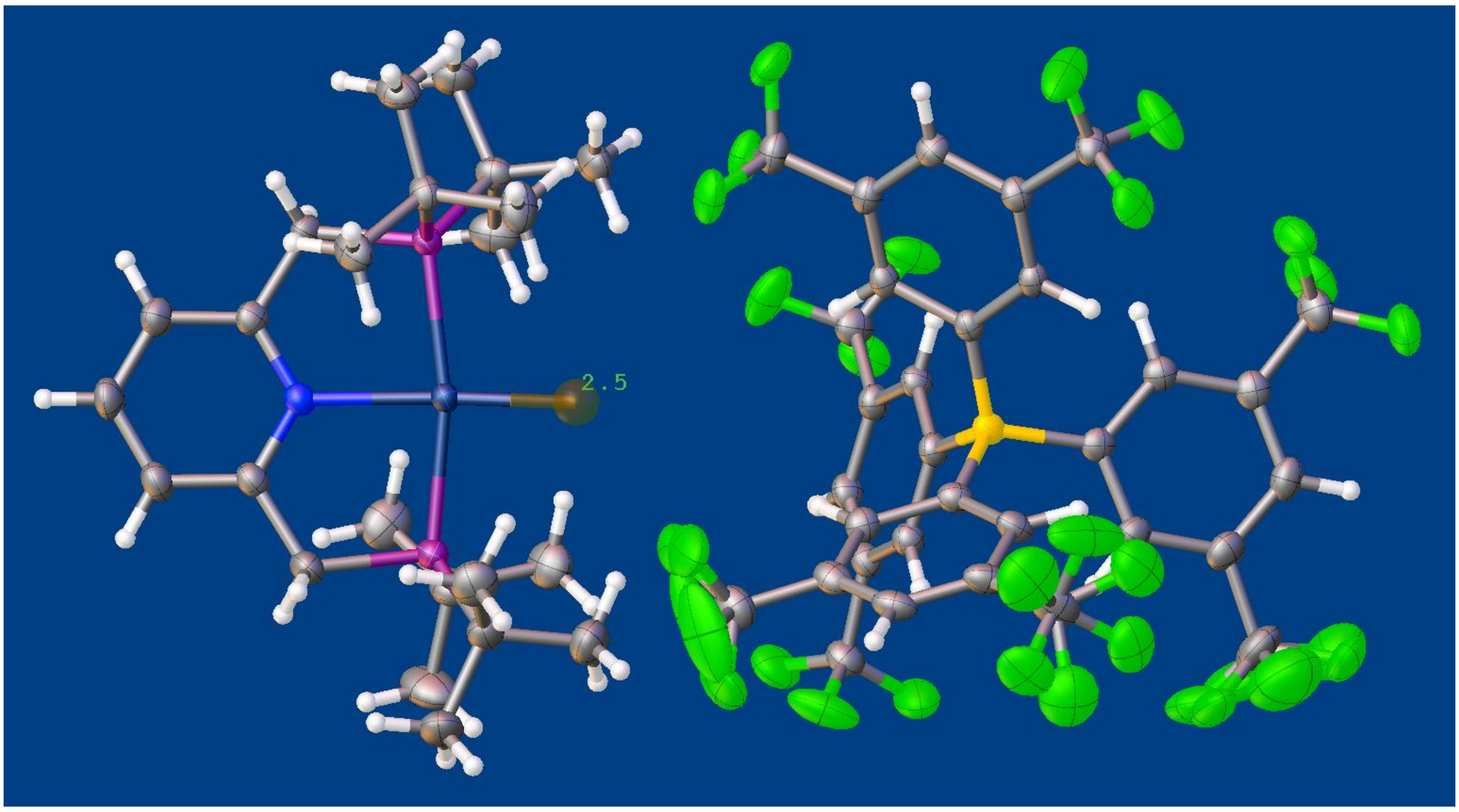

Figure S58. Difference map for the partially refined SCXRD structure of **2** + H_2 collected after 10 h. Density of Fourier peak in e$Å^{-3}$.

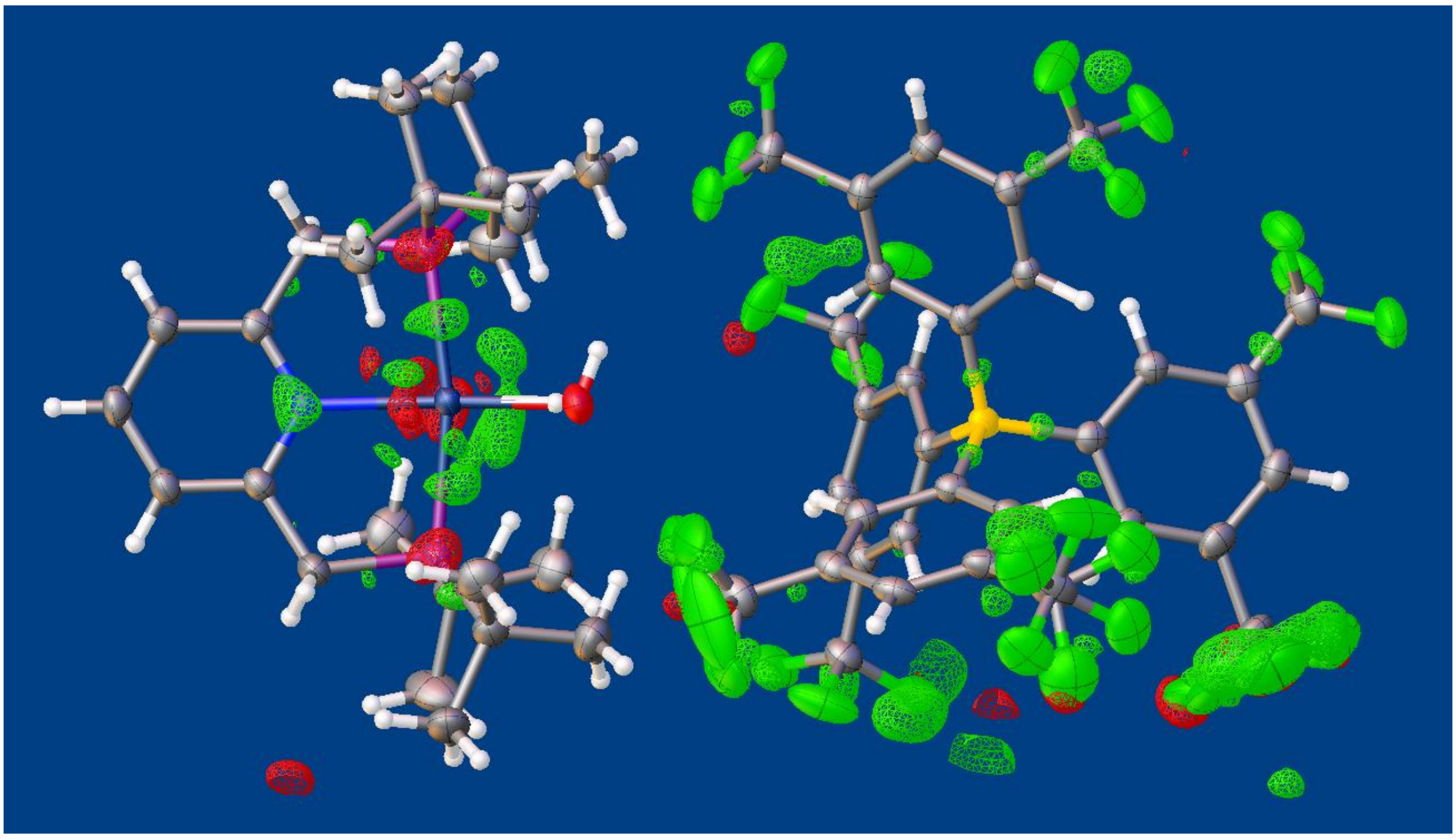

Figure S59. Difference map for the refined SCXRD structure of **2** + H_2 collected after 10 h, showing residual density > 0.3 e$Å^{-3}$. Occupancy of hydrido ligand = 78.2(9)%.

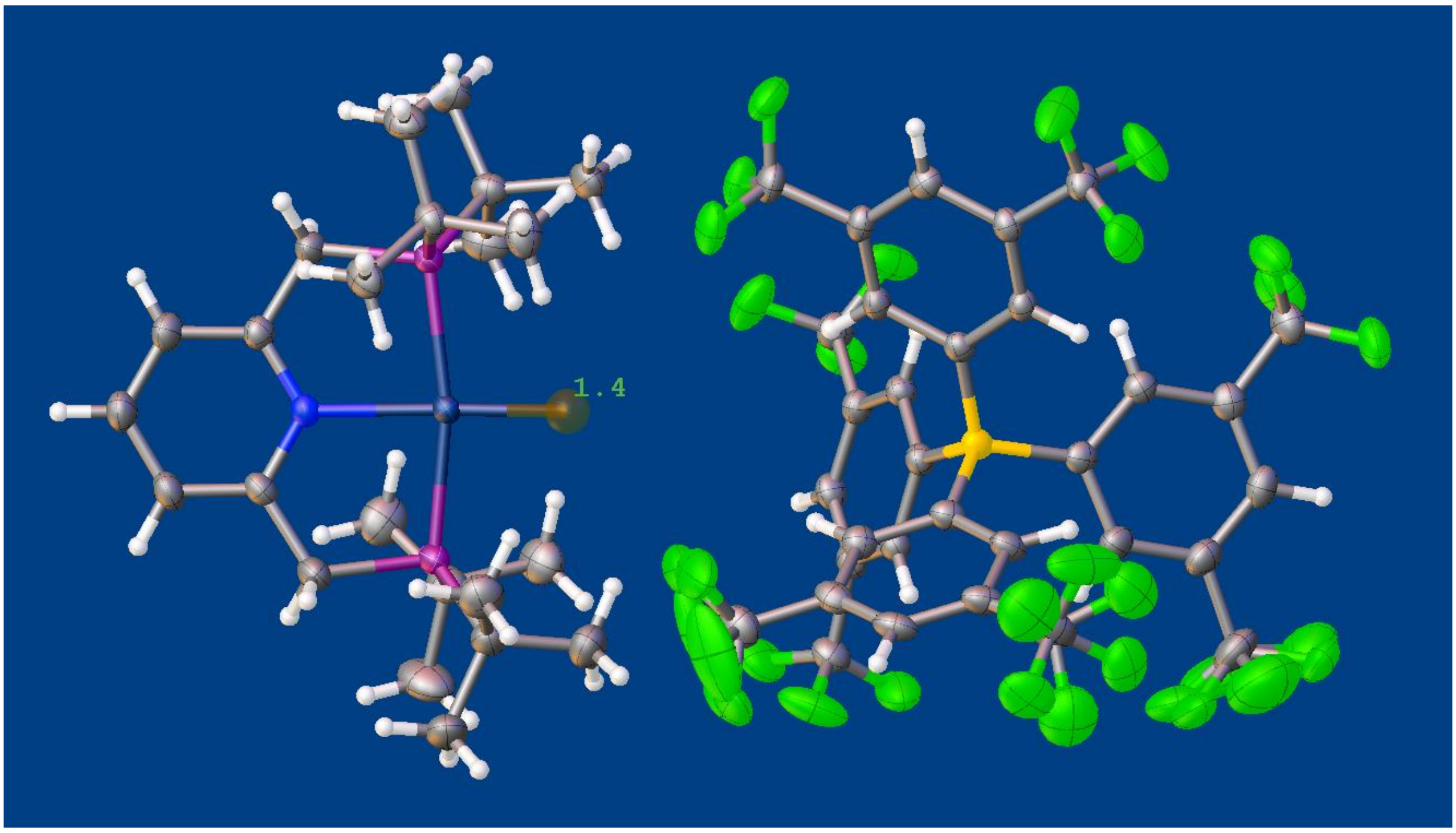

Figure S60. Difference map for the partially refined SCXRD structure of **2** + H_2 collected after 15 h. Density of Fourier peak in e$Å^{-3}$.

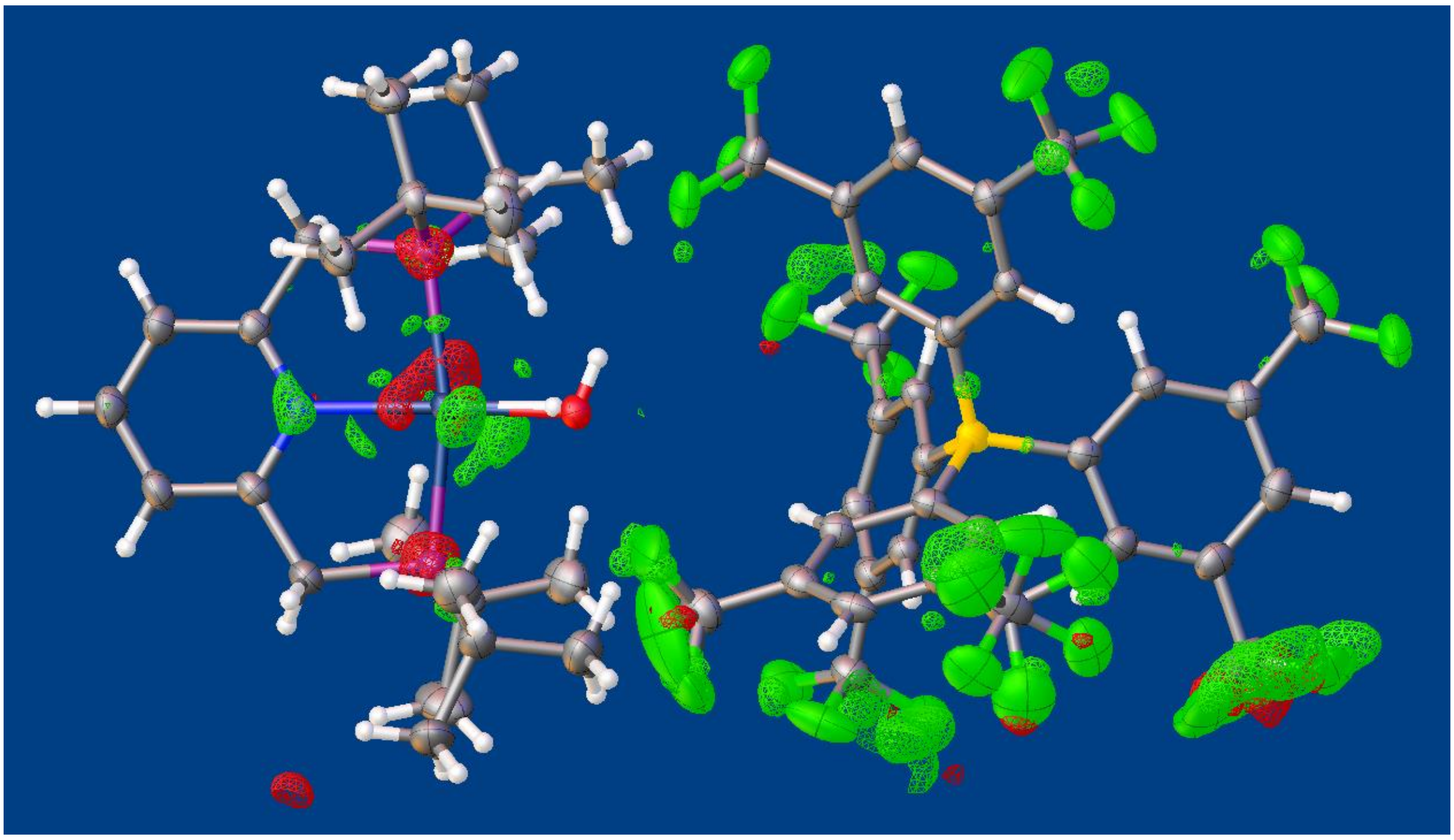

Figure S61. Difference map for the refined SCXRD structure of **2** + H_2 collected after 15 h, showing residual density > 0.3 eÅ^{-3}. Occupancy of hydrido ligand = 91.7(5)%.

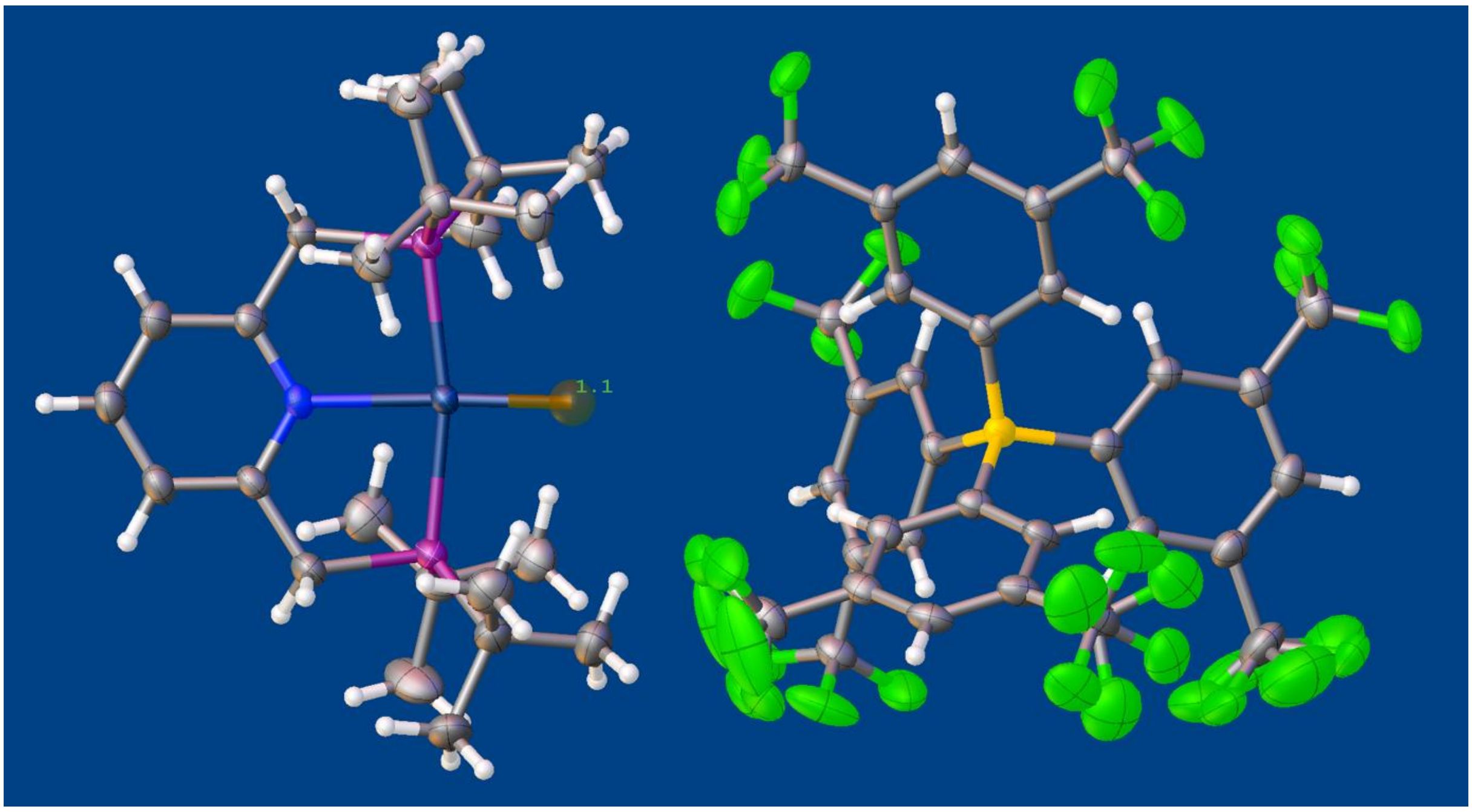

Figure S62. Difference map for the partially refined SCXRD structure of **2** + H_2 collected after 20 h. Density of Fourier peak in eÅ^{-3}.

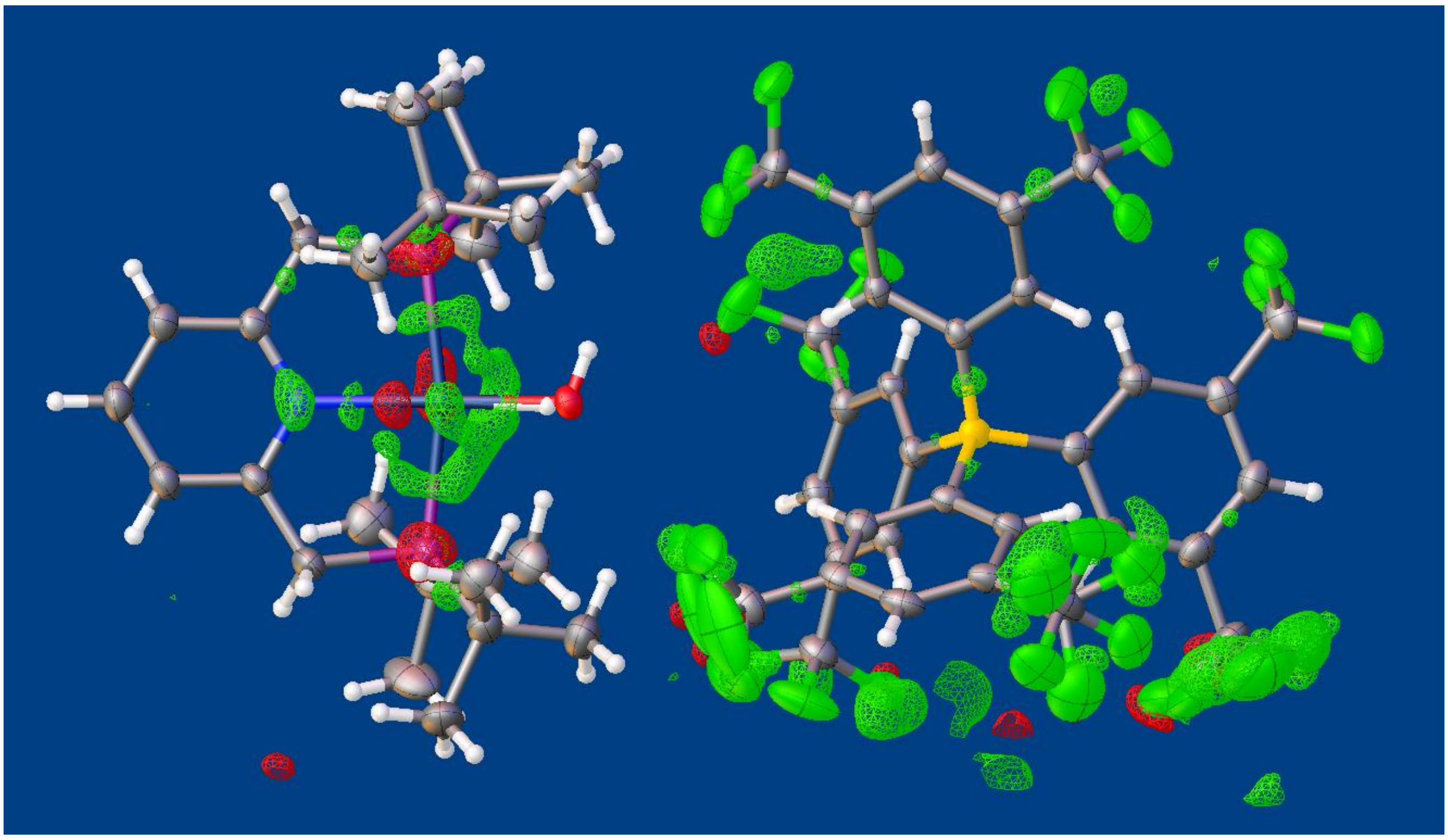

Figure S63. Difference map for the refined SCXRD structure of **2** + H_2 collected after 20 h, showing residual density > 0.3 eÅ^{-3}. Occupancy of hydrido ligand = 94.8(5)%.

7 Catalysis

Caution: mixtures of N_2O and H_2 are potentially explosive and should be handled with extreme care.[18] *This risk is compounded by the potential to form explosive mixtures of O_2 and H_2 in the presence of a metal catalyst. Hydrogenation reactions should therefore be carried out in a small scale under relatively mild conditions behind a blast shield.*

A J. Young valve NMR tube charged with microcrystalline **4** (6.9 mg, 5.01 µmol) was placed under N_2O (1 atm), sealed, and sheathed within a section of reinforced PVC hose. The end of the NMR tube was cooled to 77 K, to condense N_2O, and after 20 min backfilled with H_2 (1 atm) to give a 1:1 mixture of N_2O and H_2 (2 atm) upon warming to RT. The gas/solid reaction was run for 48 h at RT behind a blast shield and the headspace analysed by GC-TCD to determine the reaction conversion, using CH_4 as an internal standard and normalising for the detector resonance (CH_4:N_2 = 45:55). The tube was placed under high vacuum at 195 K to remove the gas mixture, sealed under static vacuum, and then removed from the PVC hose. The sample was warmed to RT and then successively placed under CO (1 atm) for 5 min, high vacuum at 77 K, and argon (1 atm) at RT. The sample was dissolved in THF (0.5 mL) and analysed by ^{1}H and ^{31}P{^{1}H} NMR spectroscopy, revealing the formation [Rh(PNP-*t*Bu)(CO)][BAr$^{F}_{4}$] **6** (δ_{31P} 79.3, $^{1}J_{RhP}$ = 120 Hz),[7] as the exclusive phosphorus containing species, and catalytic production of H_2O (δ_{1H} 2.8).[2] Average catalytic activity from duplicate runs reported in the manuscript.

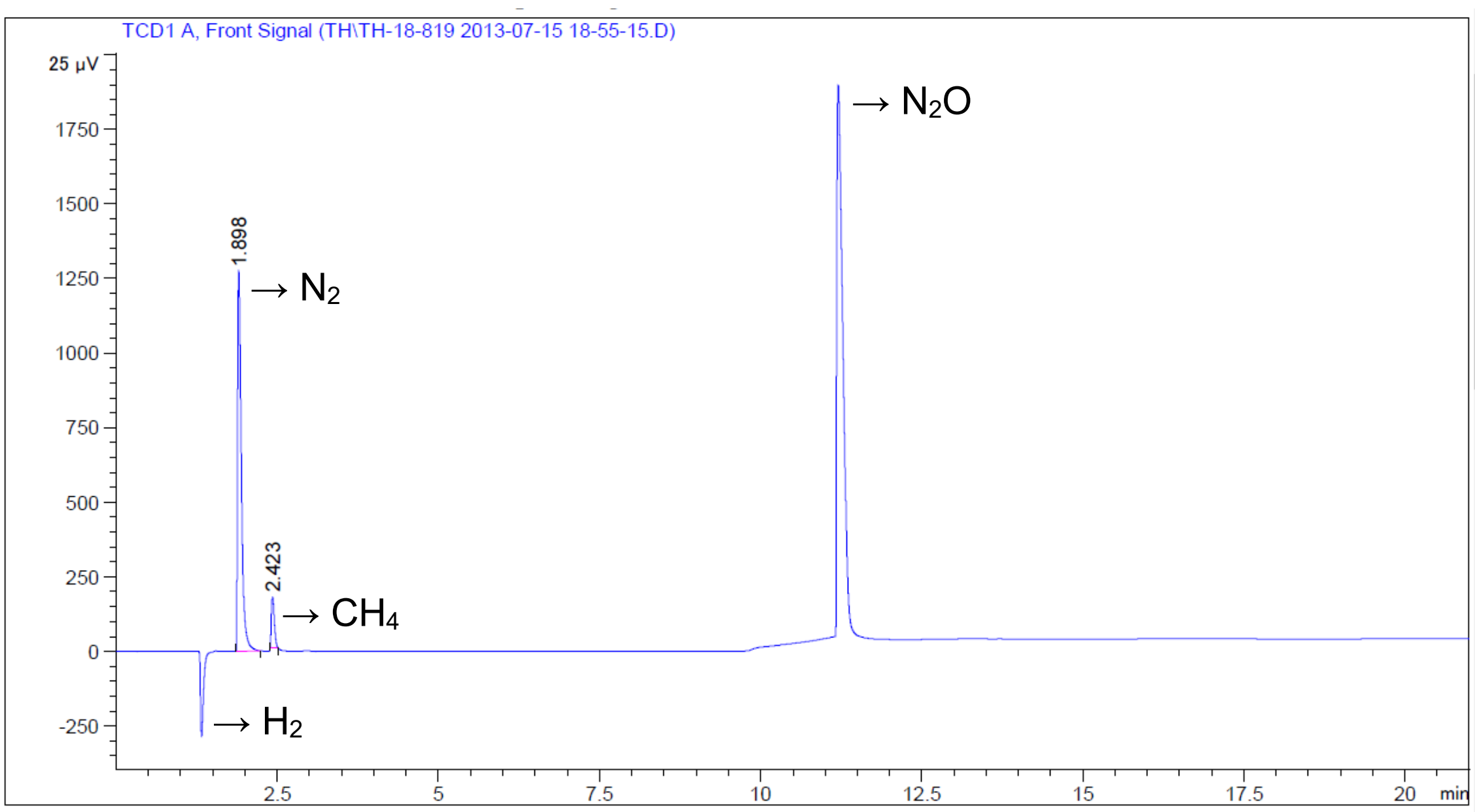

Figure S64. GC-TCD trace collected for N_2O hydrogen run 1. Calibrated N_2:CH_4 ratio = 90:10.

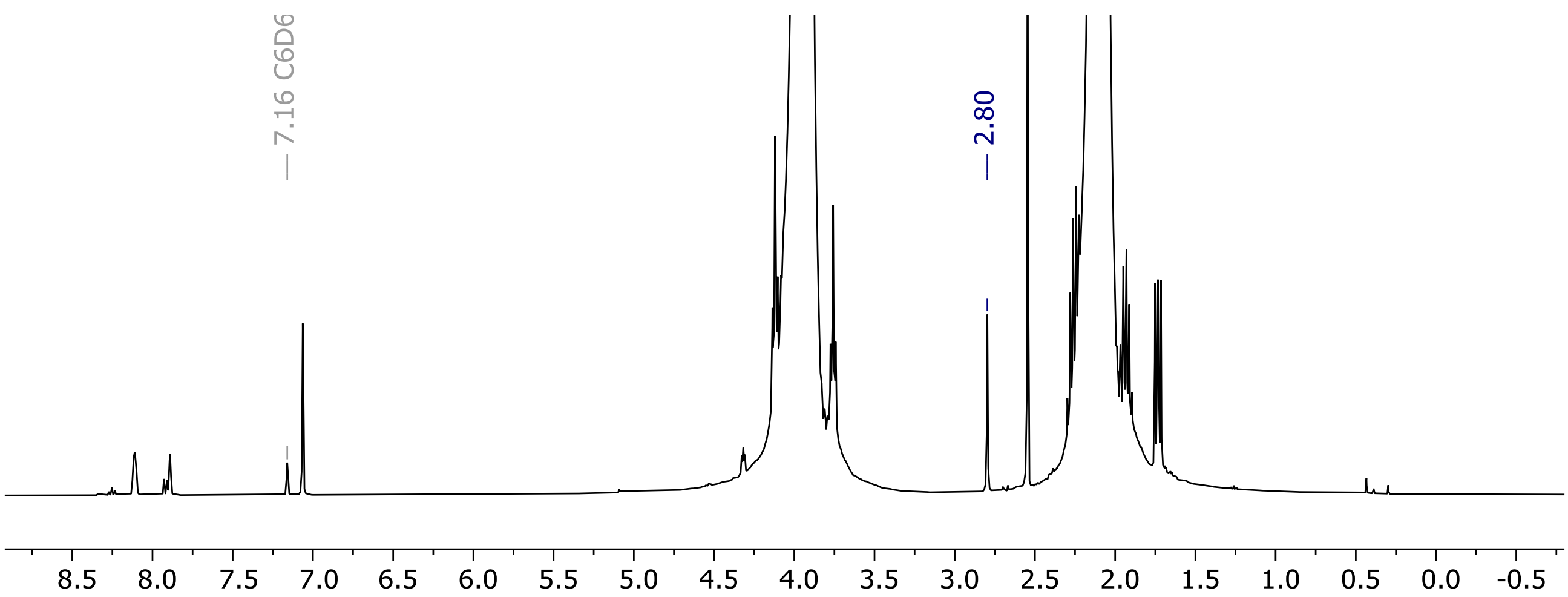

Figure S65. ^{1}H NMR spectrum recorded in THF following quenching of run 1 (400 MHz). Non-quantitative retention of H_2O during sample handling.

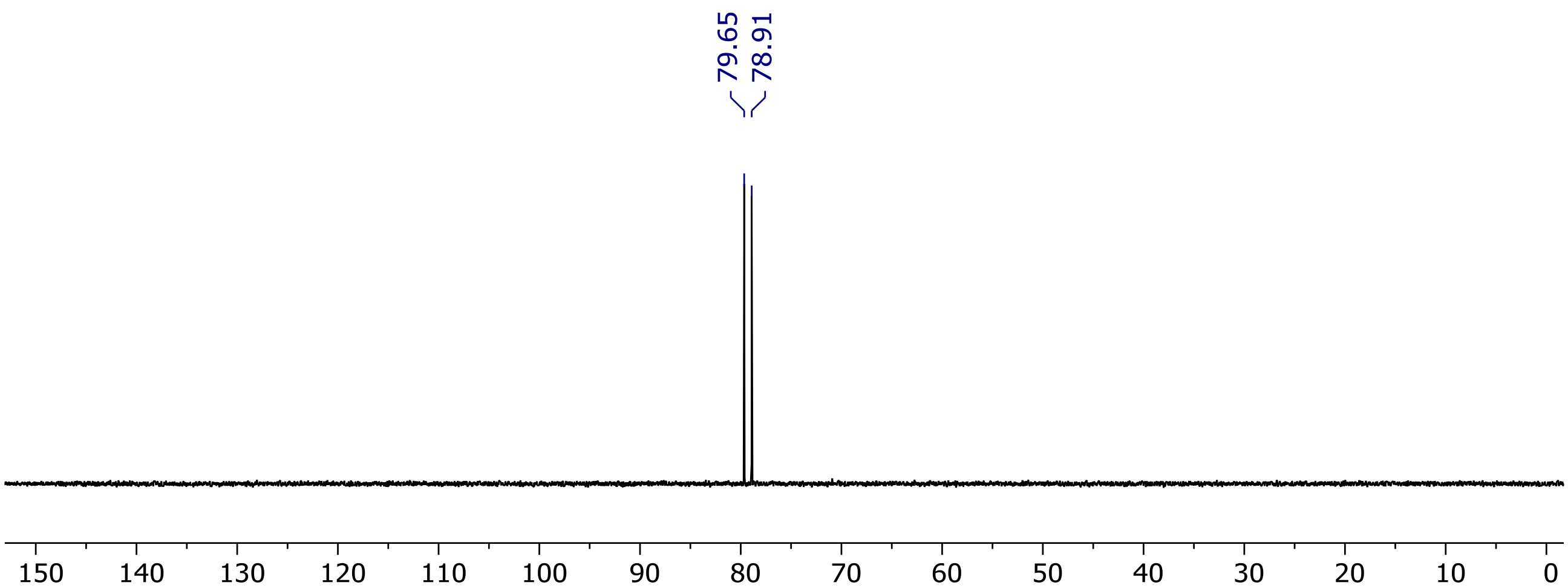

Figure S66. $^{31}P\{^{1}H\}$ NMR spectrum recorded in THF following quenching of run 1 (162 MHz).

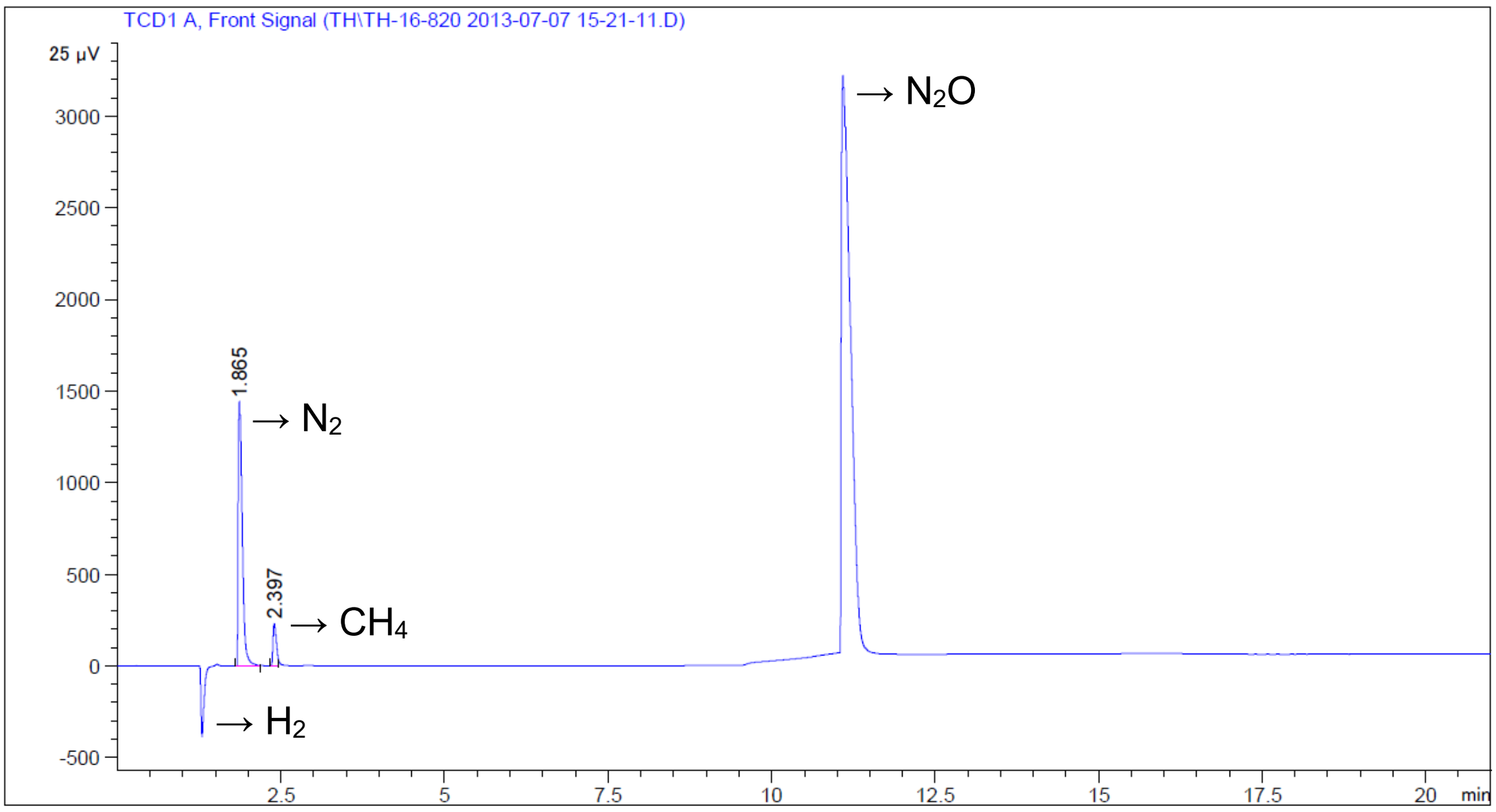

Figure S67. GC-TCD trace collected for N_2O hydrogen run 2. Calibrated N_2:CH_4 ratio = 88:12.

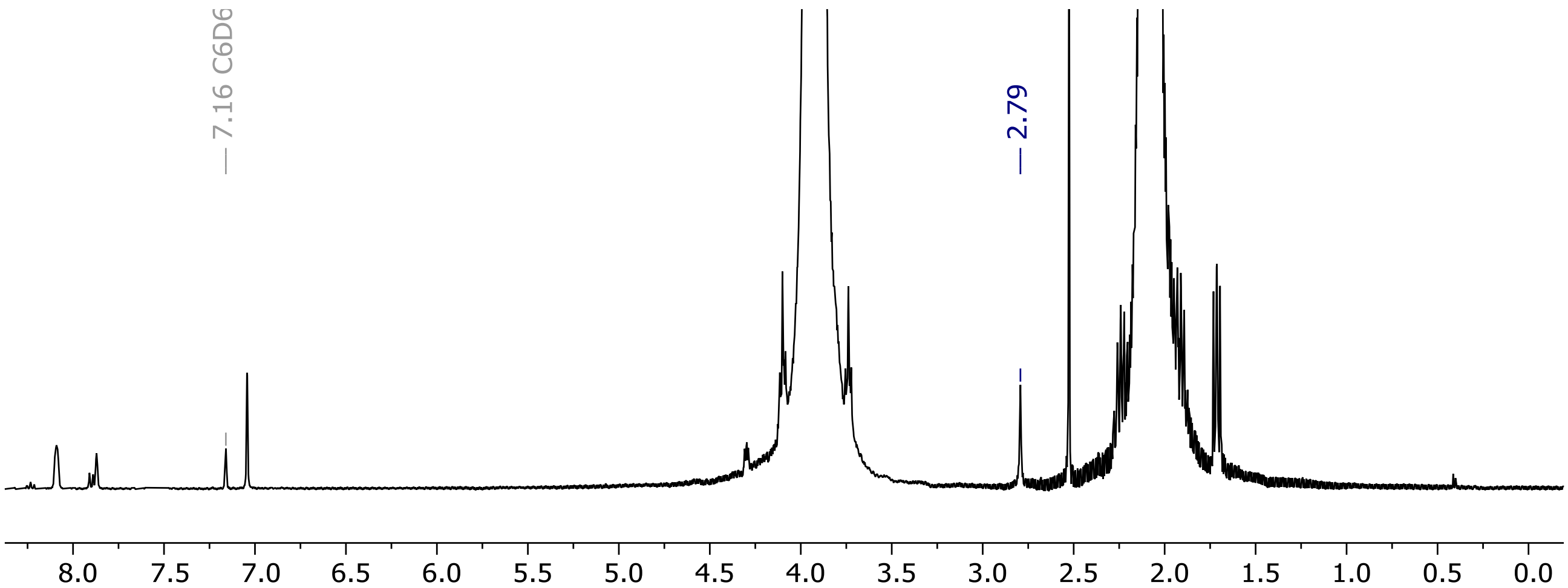

Figure S68. ^{1}H NMR spectrum recorded in THF following quenching of run 2 (400 MHz). Non-quantitative retention of H_2O during sample handling.

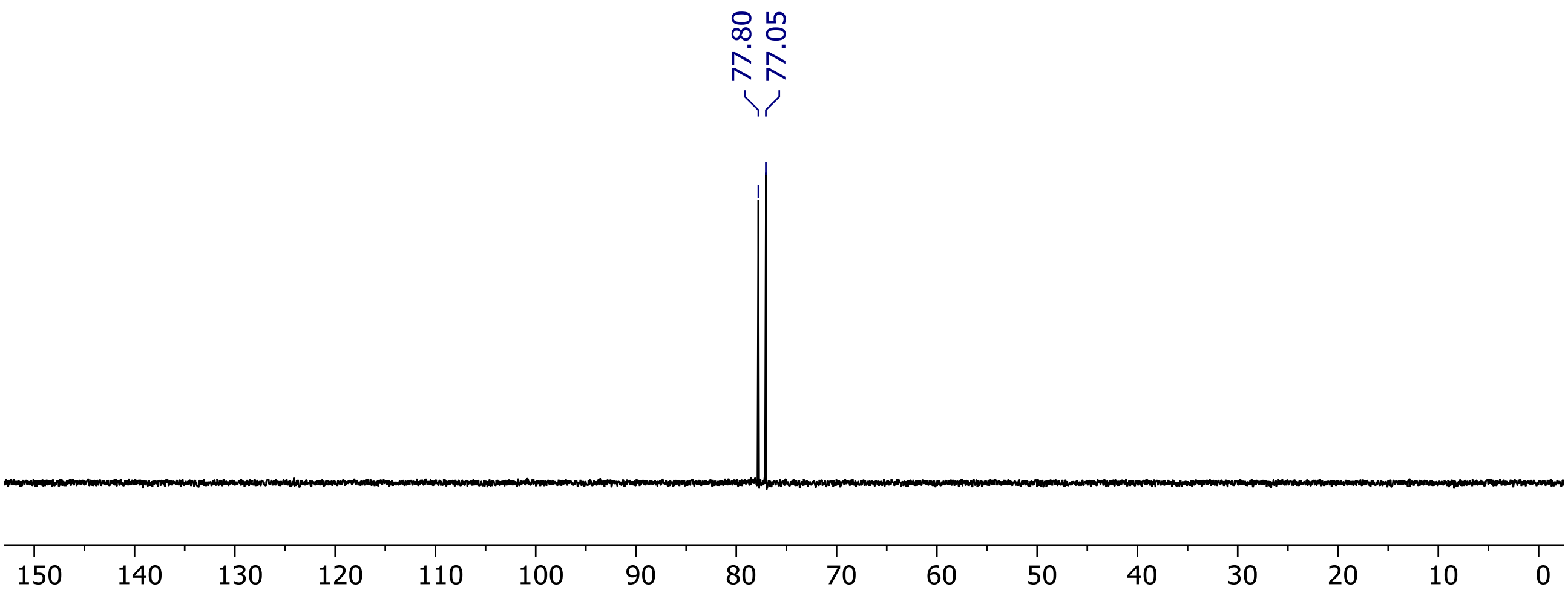

Figure S69. $^{31}P\{^{1}H\}$ NMR spectrum recorded in THF following quenching of run 2 (162 MHz).

8 Crystallographic parameters

	**4**	**1**	**2**	**2** + H_2 (10 h)	**2** + H_2 (15 h)	**2** + H_2 (20 h)
CCDC	2564408	2564403	2564404	2564405	2564406	2564407
Formula	$C_{56}H_{58}BF_{24}NP_2Rh$	$C_{55}H_{56}BF_{24}NP_2Rh$	$C_{55}H_{56}BF_{24}NOP_2Rh$	$C_{55}H_{56}BF_{24}NO_{0.22}P_2Rh$	$C_{55}H_{56}BF_{24}NO_{0.08}P_2Rh$	$C_{55}H_{56}BF_{24}NO_{0.05}P_2Rh$
M / gmol^{-1}	1376.69	1362.66	1378.66	1366.14	1363.50	1363.50
Crystal system	monoclinic	monoclinic	monoclinic	monoclinic	monoclinic	monoclinic
Space group	$P2_1$/c	$P2_1$/c	$P2_1$/c	$P2_1$/c	$P2_1$/c	$P2_1$/c
a / Å	12.58870(10)	12.57180(10)	12.60340(10)	12.57860(10)	12.58070(10)	12.58270(10)
b / Å	12.35350(10)	12.41920(10)	12.3901(2)	12.41090(10)	12.42580(10)	12.4242(2)
c / Å	39.1894(3)	39.0302(3)	39.1851(4)	39.0636(3)	39.0317(3)	39.0580(4)
α / °	90	90	90	90	90	90
β / °	96.7920(10)	96.2190(10)	96.6040(10)	96.2850(10)	96.2370(10)	96.2480(10)
γ / °	90	90	90	90	90	90
V / Å^3	6051.75(8)	6057.99(8)	6078.44(13)	6061.63(8)	6065.53(8)	6069.67(13)
Z	4	4	4	4	4	4
ρ_{calc} / gcm^{-3}	1.511	1.494	1.507	1.497	1.493	1.492
μ / mm^{-1}	3.787	3.777	3.785	3.779	3.774	3.771
F(000)	2788.0	2756.0	2788.0	2763.0	2758.0	2758.0
Crystal size / mm^3	0.197×0.118×0.077	0.217×0.175×0.090	0.181×0.163×0.088	0.194×0.145×0.092	0.168×0.096×0.087	0.181×0.091×0.061
2Θ range / °	13.004 to 148.992	13.05 to 148.992	13.002 to 149.006	13.04 to 148.994	13.044 to 149	13.038 to 149.008
Reflect collected	78565	77355	78494	79310	79265	79452
Indep reflect	12286	12347	12358	12338	12325	12359
R_{int}/R_{sigma}	0.0307/0.0210	0.0352/0.0214	0.0378/0.0257	0.0365/0.0254	0.0358/0.0255	0.0359/0.0259
Data/restr/param	12286/441/863	12347/442/857	12358/441/862	12338/446/867	12325/446/861	12359/446/861
GoF on F^2	1.029	1.097	1.041	1.025	1.013	1.017
R_1 [$I \geq 2\sigma$(I)]	0.0341	0.0381	0.0404	0.0357	0.0367	0.0379
wR_2 [all data]	0.0895	0.0910	0.1057	0.0954	0.0975	0.1021
Peak/hole / eÅ^{-3}	0.97/-0.64	0.47/-0.50	0.95/-0.85	0.88/-0.74	0.75/-0.62	0.94/-0.72

9 EPR spectroscopic parameters

Table S1. Spin Hamiltonian parameters for **4**, **1**, **2** and corresponding solvent adducts.[a]

Complex	Cont.	g_1	g_2	g_3	A_1 / MHz	A_2 / MHz	A_3 / MHz
**4**	1	3.801	1.851	1.521	298	191	181
**4**·MeTHF	1	2.496	2.336	1.971	121	131	128
**1**	1	3.246	2.351	1.825	291	195	187
**1**·MeTHF	1.3	2.446	2.336	1.975	133	139	126/40[c]
**2**	1	3.231	1.921	1.716			
**2**·MeTHF	0.35	2.601	2.276	1.961	182	126	119
**2** + H_2 (1 h) = **2**[b]	0.4	3.236	1.921	1.716			
**2** + H_2 (1 h) = **1**	0.2	3.246	2.341	1.825	291	195	187
**2** + H_2 (1 h) = **1**·Solv	0.8	2.446	2.336	1.975	133	139	126/40[c]
**2** + H_2 (5 h) = **1**	0.15	3.241	2.336	1.823	290	139	187
**2** + H_2 (5 h) = **1**·Solv	0.80	2.446	2.336	1.975	133	139	125/40[c]

[a] Values of *g* reported to ± 0.001 and, where resolved, values of *A* reported to ± 15 MHz (^{103}Rh hyperfine coupling unless otherwise stated). The **g**-frame and **A**-frame are assumed to be coincident.
[b] Corresponding solvent adduct not detected. [c] ^{1}H hyperfine coupling.

10 References

1 Pike, S. D.; Crimmin, M. R.; Chaplin, A. B. Organometallic Chemistry Using Partially Fluorinated Benzenes. *Chem. Commun.* **2017**, *53*, 3615–3633.

2 Dewick, S. H.; Hood, T. M.; Han, Y.; Huband, S.; Chaplin, A. B. Rhodium-Catalysed Hydrogenation of Nitrous Oxide. *Catal. Sci. Technol.* **2025**, *15*, 4126–4129.

3 Chávez, I.; Alvarez-Carena, A.; Molins, E.; Roig, A.; Maniukiewicz, W.; Arancibia, A.; Arancibia, V.; Brand, H.; Manríquez, J. M. Selective Oxidants for Organometallic Compounds Containing a Stabilising Anion of Highly Reactive Cations: $(3,5(CF_3)_2C_6H_3)_4B^-)Cp_2Fe^+$ and $(3,5(CF_3)_2C_6H_3)_4B^-)Cp^*{}_2Fe^+$. *J. Organomet. Chem.* **2000**, *601*, 126–132.

4 (a) Hanson, S. K.; Heinekey, D. M.; Goldberg, K. I. C−H Bond Activation by Rhodium(I) Phenoxide and Acetate Complexes: Mechanism of H−D Exchange between Arenes and Water. *Organometallics* **2008**, *27*, 1454–1463; (b) Kloek, S. M.; Heinekey, D. M.; Goldberg, K. I. C−H Bond Activation by Rhodium(I) Hydroxide and Phenoxide Complexes. *Angew. Chem. Int. Ed.* **2007**, *46*, 4736–4738.

5 Neumann, E.; Pfaltz, A. Synthesis and Characterization of Cationic Rhodium Complexes with Stable Silylenes. *Organometallics* **2005**, *24*, 2008–2011.

6 Pelczar, E. M.; Emge, T. J.; Krogh-Jespersen, K.; Goldman, A. S. Unusual Structural and Spectroscopic Features of Some PNP-Pincer Complexes of Iron. *Organometallics* **2008**, *27*, 5759–5767.

7 Gyton, M. R.; Hood, T. M.; Chaplin, A. B. A Convenient Method for the Generation of {Rh(PNP)}$^+$ and {Rh(PONOP)}$^+$ Fragments: Reversible Formation of Vinylidene Derivatives. *Dalton Trans.* **2019**, *48*, 2877–2880.

8 Pregosin, P. S. *NMR in Organometallic Chemistry*, Wiley, **2012**, pp. 251–254.

9 Stoll, S.; Schweiger, A. EasySpin, a Comprehensive Software Package for Spectral Simulation and Analysis in EPR. *J. Magn. Reson.* **2006**, *178*, 42–55.

10 CrysAlis PRO, Rigaku Oxford Diffraction, **2026**.

11 (a) Sheldrick, G. M. SHELXT – Integrated Space-Group and Crystal-Structure Determination. *Acta Cryst.* **2015**, *A71*, 3–8; (b) Sheldrick, G. M. Crystal Structure Refinement with SHELXL. *Acta Cryst.* **2015**, *C71*, 3–8.

12 Dolomanov, O. V.; Bourhis, L. J.; Gildea, R. J.; Howard, J. A. K.; Puschmann, H. OLEX2: A Complete Structure Solution, Refinement and Analysis Program. *J. Appl. Cryst.* **2009**, *42*, 339–341.

13 Lingafelter, E. C.; Zakrzewski, G. A.; Ghilardi, C. A. Structure of the Cobalt(III) and Cobalt(II) Complexes of the Cage Ligand 1,8-Bis(Fluoroboro)-2,7,9,14,15,20-Hexaoxa-3,6-10,13,16,19-Hexaaza-4,5,11,12,17,18-Hexamethylbicyclo[6.6.6]Eicosa-3,5,10,12,16,18-Hexaene. *J. Am. Chem. Soc.* **1971**, *93*, 4411–4415.

14 Hall, M. M.; Veeraraghavan, V. G.; Rubin, H.; Winchell, P. G. The Approximation of Symmetric X-ray Peaks by Pearson Type VII Distributions. *J. Appl. Crystallogr.* **1977**, *10*, 66–68.

15 Altomare, A.; Cuocci, C.; Giacovazzo, C.; Moliterni, A.; Rizzi, R.; Corriero, N.; Falcicchio, A. EXPO2013: A Kit of Tools for Phasing Crystal Structures from Powder Data. *J. Appl. Crystallogr.* **2013**, *46*, 1231–1235.

16 Gyton, M. R.; Leforestier, B.; Chaplin, A. B. Rhodium(I) Pincer Complexes of Nitrous Oxide. *Angew. Chem. Int. Ed.* **2019**, *58*, 15295–15298.

17 Chaplin, A. B.; Weller, A. S. [Rh{NC_5H_3-2,6-($CH_2P^tBu_2)_2$}(PCy_3)][$BAr^F{}_4$]: A Latent Low-Coordinate Rhodium(I) PNP Pincer Compound. *Organometallics* **2011**, *30*, 4466–4469.

18 (a) Nappen, S. T.; Gamboa-Carballo, J. J.; Tschanen, E.; Ricatto, F.; Wörle, M. D.; Thomas, A.; Trincado, M.; Grützmacher, H. Water Serving as Cocatalyst for the Highly Efficient Homogeneously Catalyzed Conversion of N_2O/H_2 Mixtures with Optimized Rhodium NHC Complexes. *Angew. Chem. Int. Ed.* **2025**, *64*, e202502616; (b) Wang, L.-Q.; Li, T.; Ma, H.-H. Explosion Behaviors of Hydrogen-Nitrous Oxide Mixtures at Reduced Initial Pressures. *Process Saf. Environ. Prot.* **2021**, *153*, 11–18; (c) Wang, L.-Q.; Ma, H.-H.; Shen, Z.-W. Explosion Characteristics of H_2/N_2O and CH_4/N_2O Diluted with N_2. *Fuel* **2020**, *260*, 116355.
